# Speciation, population structure, and demographic history of the Mojave Fringe-toed Lizard (*Uma scoparia*), a species of conservation concern

**DOI:** 10.1002/ece3.1111

**Published:** 2014-05-24

**Authors:** Andrew D Gottscho, Sharyn B Marks, W Bryan Jennings

**Affiliations:** 1 Department of Biological Sciences, Humboldt State University1 Harpst Street, Arcata, California, 95521; 2 Department of Biology, University of CaliforniaRiverside, California, 92521

**Keywords:** Coalescent simulations, conservation genetics, phylogeography, reptiles, speciation

## Abstract

The North American deserts were impacted by both Neogene plate tectonics and Quaternary climatic fluctuations, yet it remains unclear how these events influenced speciation in this region. We tested published hypotheses regarding the timing and mode of speciation, population structure, and demographic history of the Mojave Fringe-toed Lizard (*Uma scoparia*), a sand dune specialist endemic to the Mojave Desert of California and Arizona. We sampled 109 individual lizards representing 22 insular dune localities, obtained DNA sequences for 14 nuclear loci, and found that *U. scoparia* has low genetic diversity relative to the *U. notata* species complex, comparable to that of chimpanzees and southern elephant seals. Analyses of genotypes using Bayesian clustering algorithms did not identify discrete populations within *U. scoparia*. Using isolation-with-migration (IM) models and a novel coalescent-based hypothesis testing approach, we estimated that *U. scoparia* diverged from *U. notata* in the Pleistocene epoch. The likelihood ratio test and the Akaike Information Criterion consistently rejected nested speciation models that included parameters for migration and population growth of *U. scoparia*. We reject the Neogene vicariance hypothesis for the speciation of *U. scoparia* and define this species as a single evolutionarily significant unit for conservation purposes.

## Introduction

Despite their extreme climate and apparent desolation, the deserts of North America comprise one of only five global high-biodiversity wilderness areas (Mittermeier et al. 2003). Therefore, an understanding of the environmental factors influencing speciation in this region is not only of theoretical interest, but is paramount for effective biodiversity conservation (Vandergast et al. 2013). These deserts were impacted by both Neogene plate tectonics and Quaternary climatic fluctuations, yet it remains unclear how these geological events influenced the timing and mode of speciation (Wood et al. 2012). Some have argued that Pleistocene glacial cycles promoted speciation through habitat fragmentation, dispersal, and range expansion (e.g., Rovito 2010). Although ice sheets did not cover the North American deserts during the Pleistocene, pollen extracted from fossilized packrat (*Neotoma*) middens show that during the last glacial maximum (LGM) 18 kya, the Mojave Desert was dominated by a mesic coniferous woodland, confining arid-adapted flora to refugia at lower elevations and latitudes in the Sonoran and Colorado Deserts (Cole 1986). The modern desert flora later expanded northward to reach their present distributions in the Holocene epoch as recently as 6 kya (Thompson and Anderson 2000). However, the importance of glacial cycles in promoting speciation in North America has been disputed (Klicka and Zink 1997; Johnson and Cicero 2004).

Alternatively, Neogene tectonic events may have been the dominant environmental force driving speciation in this region through vicariance. During the Miocene and Pliocene, southwestern North America was transformed by the San Andreas Fault (SAF) system, an active boundary between the North American and Pacific plates, resulting in the separation of the Baja California peninsula via rifting and inundation of the Gulf of California and Salton Trough (Elders et al. 1972), the uplift of the Transverse Ranges (Meisling and Weldon 1989), and the formation of the Colorado River delta (Buising 1990). Divergence dates estimated with mtDNA of multiple desert taxa, including arachnids, amphibians, reptiles, and mammals, support this latter hypothesis (Wood et al. 2012). However, gene divergence may pre-date population divergence, particularly if ancestral populations were large (Edwards and Beerli 2000). To resolve this controversy, the next step is to accurately and precisely estimate speciation times by analyzing multilocus data with coalescent models that account for gene flow and incomplete lineage sorting (Knowles and Maddison 2002).

### Study system: fringe-toed lizards

**Figure 1:**
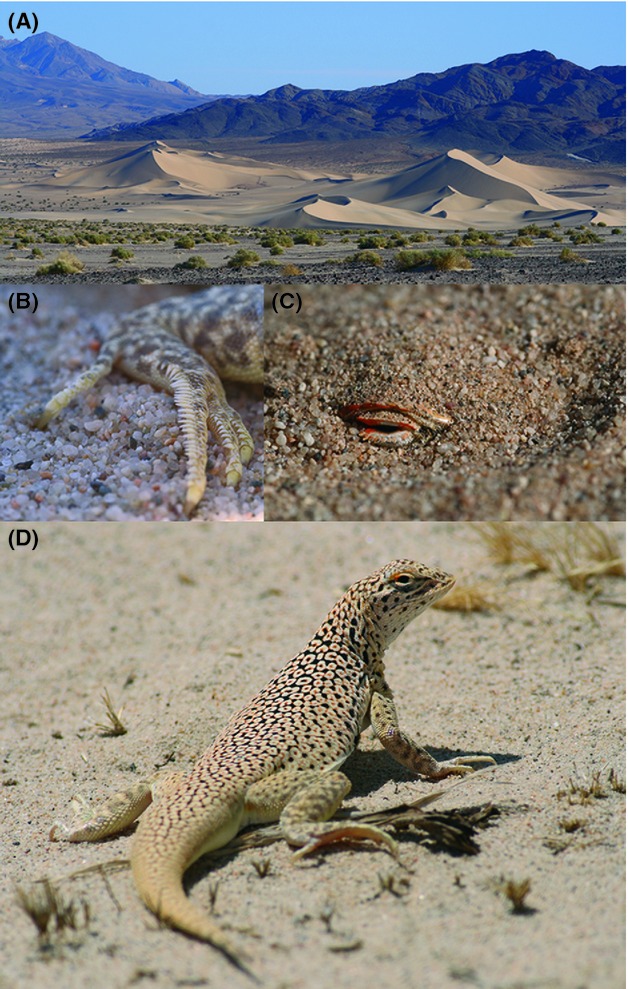
The Mojave Fringe-toed Lizard*, Uma scoparia*, is restricted to sand dunes in the Mojave Desert of California and Arizona. (A) Ibex Dunes, Death Valley National Park, the northernmost locality where this species occurs, (B) fringed toes increase traction and locomotion efficiency on loose sand, (C) a shovel-shaped snout facilities burial, and (D) an ocellated pattern increases crypsis in exposed habitats. Photographs courtesy of Cameron Rognan.

**Figure 2:**
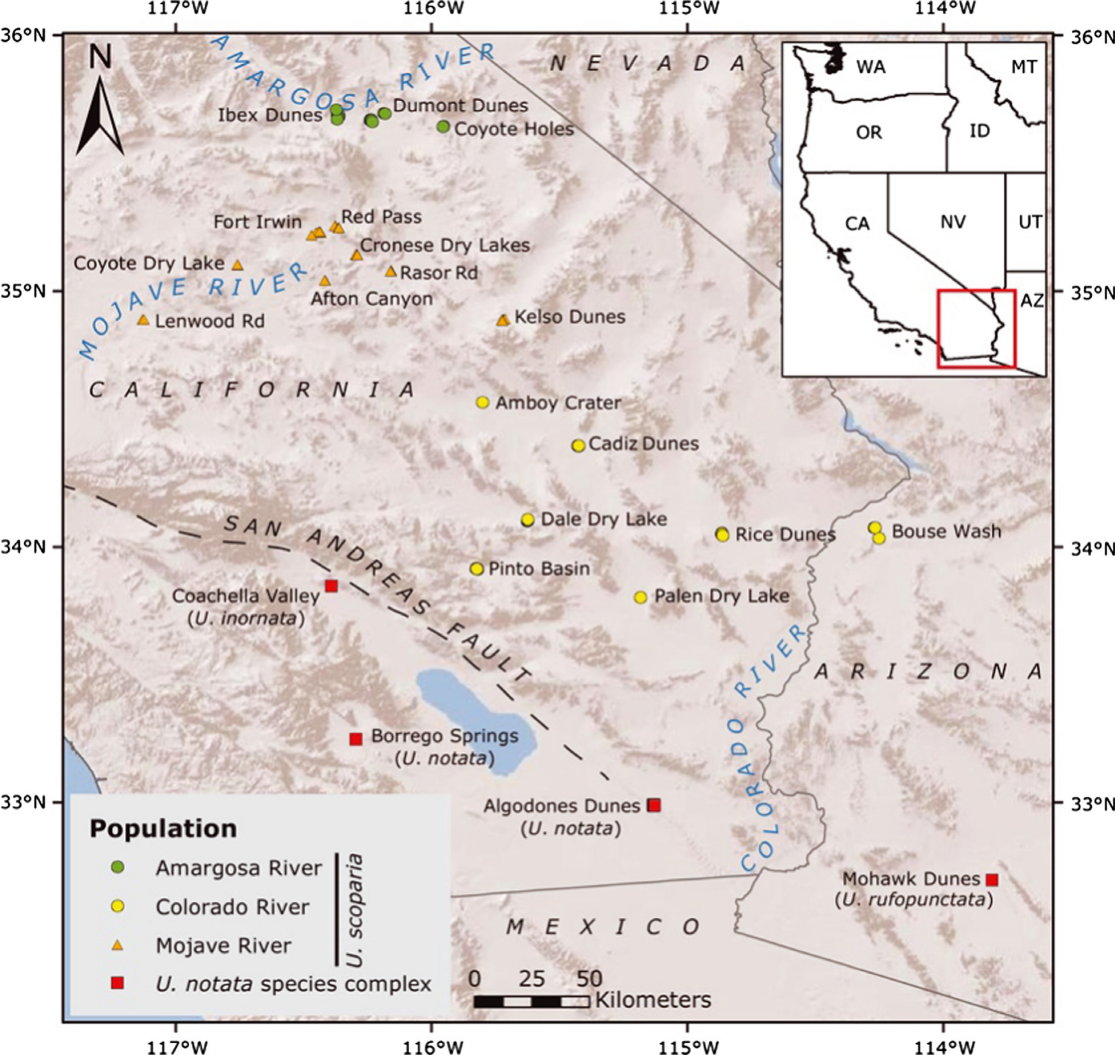
Sample localities used in this study. Green circles represent the Amargosa population, orange triangles represent the Mojave population, yellow circles represent the Colorado population, and red squares represent the *U. notata* species complex.

Fringe-toed lizards (genus *Uma*) are restricted to desert sand dunes and possess several specialized adaptations that facilitate efficient locomotion in loose sand, particularly their namesake toe fringes (Fig. 1). Sand dunes are insular and mobile, are able to migrate tens of meters downwind per year, and are tightly linked to Pleistocene glacial cycles. Increased precipitation during glacial maxima restricted dunes to narrow, continuous belts along streams and lakeshores (Enzel et al. 2003), but during interglacial periods, particularly since the LGM, evaporation exposed alluvium to the wind and promoted the growth and fragmentation of dunes (Lancaster and Tchakerian 2003; Muhs et al. 2003). For these reasons, and because different *Uma* species occur on either side of the mountains associated with the SAF (Fig. 2), fringe-toed lizards are well suited to test hypotheses regarding the interactions between Pleistocene climate change, Neogene plate tectonics, and speciation.

At least four morphologically and genetically diagnosable lineages of *Uma* occur in the Mojave, Colorado, and Sonoran deserts, although their taxonomy has been unstable (Norris 1958; Adest 1977; Trepanier and Murphy 2001; Schulte and de Queiroz 2008). Here, we focus on *U. scoparia*, the species from the Mojave Desert of California and western Arizona, and its southern sister lineage, the “*U. notata* species complex,” which is composed of three closely allied species: *U. notata* in the lower Colorado Desert of California and northeastern Baja California, *U. inornata* in the Coachella Valley of California, and *U. rufopunctata* in southwestern Arizona and northwestern Sonora. Because their insular habitat is sensitive to anthropogenic disturbances (Hedtke et al. 2007), *U. scoparia* and *U. notata* are listed as “Species of Special Concern” in California (Jennings and Hayes 1994), while *U. inornata* is currently listed as a federally threatened species (Trepanier and Murphy 2001).

Given the specialization of *Uma* for dynamic, isolated dune habitats, the historical biogeography of these lizards has generated much interest. Norris (1958) hypothesized that *U. scoparia* originated during the late Pliocene or early Pleistocene when a fraction of the ancestral *U. notata* complex dispersed northward along the Colorado River gorge into the southern Mojave Desert. After speciation, he hypothesized that glacial maxima confined *U. scoparia* to the lower, warmer southeastern portion of its range near the Colorado River before it colonized northwestern localities in the Mojave and Amargosa River drainages after the LGM. Using allozyme data, Adest (1977) estimated that *U. scoparia* diverged from *U. notata* in the late Pleistocene just before the LGM (∼0.054 mya) and that *U. notata* had heterozygosity approximately three times higher than that of *U. scoparia*, a finding consistent with Norris's peripatric speciation hypothesis. Alternatively, using mtDNA data, Murphy et al. (2006) hypothesized that *U. scoparia* speciated in the late Miocene via a vicariance event associated with the formation of the Lower Colorado River. Moreover, these authors found distinct mtDNA haplotypes in the northern Amargosa River and Red Pass populations near Death Valley and concluded that each of these populations constitutes “distinct population segments,” a legal term similar to “evolutionarily significant units” (ESUs), under the U.S. Endangered Species Act (Moritz 1994). This “Amargosa refuge” hypothesis predicts that northern populations diverged from southern populations of *U. scoparia* approximately 0.5 mya.

Here, we analyzed a multilocus dataset with coalescent models, simulations, and Bayesian clustering algorithms to investigate the speciation, population structure, and demographic history of *U. scoparia*. First, we tested hypotheses regarding the timing and mode of speciation. The Neogene vicariance hypothesis predicts an older divergence time, low levels of gene flow across the Colorado River, and similar levels of genetic diversity in both descendent species, while the dispersal hypotheses predict a Pleistocene divergence time, no gene flow subsequent to dispersal, and low genetic diversity in *U. scoparia* relative to the *U. notata* complex. Second, we evaluated hypotheses regarding post-speciation demographic history. The northern range expansion hypothesis predicts little to no population structure and recent demographic expansion within northern populations of *U. scoparia*, while the Amargosa refuge hypothesis predicts distinct ESUs within northern populations.

## Materials and Methods

### Genetic samples

Tissue samples were collected during 2008 and 2009 throughout the range of *U. scoparia* and from the *U. notata* complex in California and Arizona (Fig. 2; Table S1). Lizards were live-captured with a noose and tail tips were preserved in 95% ethanol. We used a GPS unit to record location and elevation before photographing and releasing each lizard. One sample (1743CAP) of *U. inornata* (a threatened species) was provided by the Royal Ontario Museum, Toronto, Canada. In total, we included samples of 93 *U. scoparia*, 14 *U. notata*, 1 *U. inornata*, and 1 *U*. *rufopunctata*, representing 22 isolated dune localities. Genomic DNA was extracted with DNeasy Blood and Tissue Kits (Qiagen, Valencia, CA).

### Molecular data

Due to the lack of suitable nuclear markers for *Uma*, we first attempted to discover novel anonymous loci following the protocol developed by Jennings and Edwards (2005). We developed a small-insert genomic library from an individual *U. scoparia* (BDH061, San Diego Natural History Museum, California), which allowed us to design PCR primers for four new anonymous loci (Uma03, Uma05, Uma06, and Uma08). A GenBank BLAST search was conducted to determine whether any locus was homologous with known genomic regions. We initially attempted to sequence these four loci for all 109 lizards in our study; however, preliminary analyses revealed low genetic diversity among all sampled *U. scoparia*. Therefore, given limited financial resources, we decided to maximize informative data by collecting more loci at the expense of individual sampling, so we sequenced ten more loci for a subset of individuals (44) representing all sampled dune localities. These include six anonymous loci (Sun07, Sun08, Sun10, Sun12, Sun18, and Sun28) developed for the phrynosomatid lizard *Sceloporus undulatus* (Rosenblum et al. 2007) and four exons (BDNF, PNN, R35, and RAG-1) that have been used in studies of other phrynosomatids (Leaché 2009). Primer sequences and annealing temperatures are shown in Table S2. PCR products were sequenced using an ABI3730 sequencer (Applied Biosystems, Foster City, CA). We used CodonCode Aligner v3.5.2 (CodonCode Inc., Dedham, MA) to resolve heterozygous indels, call heterozygous SNPs, and create sequence alignments.

### Haplotype inference and intralocus recombination

We used PHASE v2.1 (Stephens et al. 2001) with SeqPHASE (Flot 2010) to infer the most probable haplotypes (probability cutoff 0.50) from our directly sequenced PCR products. We searched for evidence of recombination using RDP3 (Martin et al. 2010).

### Summary statistics

We used DNAsp v5.10 (Librado and Rozas 2009) to calculate summary statistics for both datasets, including nucleotide diversity (*π*), the number of segregating sites (S), and the number of fixed differences (F_NS_) between *U. scoparia* and *U. notata*, as well as between the Amargosa and Mojave populations within *U. scoparia*. To compare *π* among species and populations, we estimated the mean *π* values across all loci and used an unpaired two-tailed t-test (Balakrishnan and Edwards 2009). Tajima's D (Tajima 1989) was calculated to ensure that the loci met the neutrality assumption of the IM model.

### Gene trees

In order to assess lineage sorting, we constructed gene trees using maximum-likelihood criteria with RAxML v7.3.0 (Stamatakis 2006) implemented in RAxML GUI v1.0 (Silvestro and Michalak 2012). We elected to use the GTR+G+I mutation model due to the large proportion of invariant sites in our dataset (see Results). We selected the best tree from 100 runs and plotted bootstrap values based on 1,000 replicates. Gene trees were rooted using the midpoint method. We also constructed gene trees using MrBayes v3.2.2 (Ronquist et al. 2012) to determine whether different methods (maximum-likelihood vs. Bayesian) of gene tree estimation would alter our conclusions. We determined the appropriate substitution model using the Akaike Information Criterion (AIC) implemented in jModelTest v2.1.4 (Guindon and Gascuel 2003; Darriba et al. 2012). For each locus, we ran two independent analyses of 10 million generations each with four chains, sampling the tree space every 10,000 generations. Convergence was assessed using Tracer v1.5 (Rambaut and Drummond 2009) to ensure that ESS values were >200 for each run. A 50% majority rule consensus tree representing both runs was rooted using the midpoint method.

### Population structure

We used Structurama v2.0 (Huelsenbeck et al. 2011) and Geneland v4.0.3 (Guillot et al. 2005) to detect population structure and to assess the panmictic population assumption of IM. Because these algorithms may be biased by missing data, we excluded 2 loci and 67 individuals for which we had the least complete sampling, resulting in a final genotype matrix of 42 individuals (31 *U. scoparia*, 9 *U. notata*, 1 *U. inornata*, and 1 *U. rufopunctata*) at 12 loci. By excluding individual lizards and loci with the most missing data, we increased the completeness of the genotype matrix from 40.3% to 81.5% while still representing all of the geographic localities sampled in this study.

We analyzed three versions of the genotype matrix using Structurama: 1) the full matrix with all species (12 loci × 42 individuals); 2) *U. scoparia* and *U. notata* only (excluding *U. inornata* and *U. rufopunctata*); and 3) *U. scoparia* only. We set the prior number of populations to a random variable (Dirichlet Process Prior) characterized by a gamma distribution and used the no-admixture model. We ran each analysis three separate times with 5 chains (temperature 0.2) for 100 million MCMC cycles, discarding the first 10% of cycles as burn-in. For the Geneland analyses, we set the prior range of number of populations between 1 and 8, with an uncorrelated allele frequency and no-admixture model, because when the number of populations is unknown, the correlated allele frequency model tends to overinflate the number of populations (Guillot 2008). As in the Structurama analyses, we ran six analyses per matrix as described previously (all species included, *U. scoparia* and *U. notata* only, *U. scoparia* only), three analyses utilizing the spatial model (which explicitly incorporates GPS coordinates) and three with the nonspatial model (which ignores spatial data), for a total of 18 analyses. We ran the Markov chain for 20 million steps, sampling every 1,000 steps, discarding the first 10% of steps as burn-in. To assess convergence, we ensured that the posterior probability density trace plots showed no trends, and verified that the results of the three independent runs were consistent.

### Isolation-with-migration models

The program IM (Hey and Nielsen 2004; Hey 2005) was used to estimate effective population sizes (*N*_*notata*_*, N*_*scoparia*_*, N*_ancestor_), population divergence time (*T*), the splitting parameter (*s*), and migration rates (*m*_*1*_, *m*_*2*_) between *U. scoparia* and the *U. notata* complex. Because the Structurama and Geneland analyses found population structure within the *U. notata* complex, we analyzed two treatments of our data to determine the sensitivity of our results to violations of this assumption. Treatment 1 included all species, grouping *U. notata*, *U. inornata*, and *U. rufopunctata* together as a single population, while treatment 2 excluded all sequences of *U. inornata* and *U. rufopunctata*, thereby satisfying the two-population assumption. Several preliminary runs were conducted to optimize priors. The final simulations were carried out with an HKY mutation model (Hasegawa et al. 1985), a geometric heating scheme (g1 = 0.85 and g2 = 0.95), 15 chains, and a chain length of 2 million steps after a burn-in of 1 million steps. To assess convergence, three separate runs were conducted per treatment with different random number seeds. Effective sample size (ESS) values were monitored to ensure proper mixing of the Markov chain.

To convert raw parameter estimates into demographic quantities, we used a fossil-calibrated Bayesian phylogeny of iguanian lizards (Townsend et al. 2011) to estimate mutation rates for the following four loci: 2.2 × 10^−9^ substitutions/site/year for BDNF, 2.19 × 10^−9^ for RAG-1, 2.23 × 10^−9^ for PNN, and 4.25 × 10^−9^ for R35. Because the mutation rates for our anonymous loci are unknown and fossils of *Uma* are lacking, we assumed a mutation rate of 2.56 × 10^−9^ substitutions/site/year for our anonymous loci based on published rates of anonymous loci in birds (Lee and Edwards 2008; see Discussion for an assessment of the impact of mutation rate uncertainty on our conclusions). The geometric mean of the per-locus mutation rates (*μ*) was calculated and then used to compute the divergence time by using *T* = *t*/*μ*. To calculate effective population size (*N*), we used *N* = *θ*/(4G*μ*), where the generation time (G) is 2 years (Mayhew 1966). To estimate the population migration rate, we used 2*Nm* = *θ* * m/2. To calculate the number of founders of *U. scoparia*, we calculated (1−*s*) * *θ*_*ancestor*_.

We used the likelihood ratio test (LRT) and the Akaike Information Criterion (AIC) to test nested demographic models in IMa (Hey and Nielsen 2007), for example, to determine whether simpler models that excluded migration or *θ* parameters were a better fit to the data. The LRT uses the chi-squared test to reject nested models above a certain threshold (*P* < 0.05), but provides no ranking of the best model (Hey and Nielsen 2007), while the AIC allows for ranking of the full and nested models based on the number of parameters (k) and the log(P) value reported from IMa (Carstens et al. 2009, 2010). For these analyses, we used our full dataset (treatment 1) with the same priors as in our IM analyses. We ran the Markov chain twice with different random seeds for 2 million steps, sampling genealogies every 100 steps. After checking for convergence as described above, we randomly subsampled 39,000 genealogies for the LRT. We also used IMa to test for divergence between the Amargosa River and Mojave River populations of *U. scoparia*, using an identical chain length and heating scheme as described earlier, but with adjusted prior distributions for *θ*, *T*, and *m*.

### Hypothesis testing using coalescent simulations

**Figure 3:**
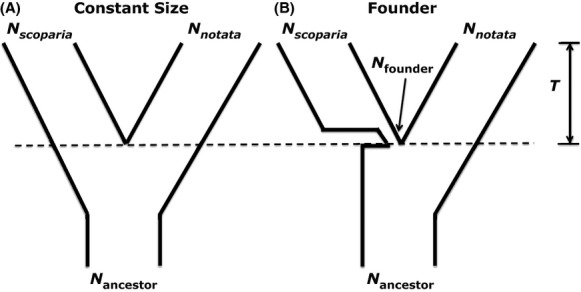
Speciation models used in hypothesis testing. Both models contain effective population sizes (*N*_*scoparia*_, *N*_*notata*_, *N*_ancestor_) and population divergence time (*T*) parameters. The founder model contains an additional parameter to characterize the effective founding population size, *N*_founder_.

We tested six a priori speciation models for *U. scoparia*, plus two models suggested by our IM results. Values for each of the model's demographic parameters were supplied by the results of our IM analyses under treatment 1 (see Results). All eight models contain the demographic parameters *N*_*scoparia*_, *N*_*notata*_, *N*_ancestor_, and *T* (Fig. 3). The first four models do not include bottlenecks or founder events (constant size). These models are Model 1a (divergence 0.054 Ma; Adest 1977), Model 2a (divergence ∼1.0 Ma; this study), Model 3a (divergence 2 Ma; Norris 1958), and Model 4a (divergence 6 Ma; Murphy et al. 2006). Additionally, we created four variants of the previous models by incorporating a founder population size (*N*_founders_) of 241 individuals (Table 5) for *U. scoparia* (Models 1b–4b). The amount of time in which the founder population exists at such a low population size is difficult to determine, so we arbitrarily chose an interval defined as 5% of the overall divergence time.

For each model, we generated a null distribution of 1,000 gene trees using Mesquite v2.75 (Maddison and Maddison 2005). Simulated trees contained 70 *U. scoparia* and 15 *U. notata* alleles, roughly corresponding to the average numbers in our observed data (see Results). Next, we simulated a 500-bp sequence matrix for each gene tree under the HKY model. We identified optimal scaling factors by finding the value that yields average pairwise sequence divergences in our simulated sequences resembling those observed in our actual data (Maddison and Knowles 2006). PAUP* (Swofford 2003) was used to reconstruct each simulated gene tree using parsimony. We then used Mesquite to calculate Slatkin's *s* statistic, a measure of degree of lineage sorting assuming no postdivergence gene flow, for all observed and simulated trees (Slatkin and Maddison 1989; Carstens et al. 2005). We developed a simple test statistic, the percentage sorted trees (PST) that are reciprocally monophyletic or monophyletic in one haplotype clade with respect to the species tree (*s* = 1). We tested models by comparing the observed and expected PST values using a chi-square test. A model was rejected if *P* < 0.05. Although we used Slatkin's *s* to quantify lineage sorting, PST can also be calculated using other metrics of lineage sorting such as the genealogical sorting index (Cummings et al. 2008).

To evaluate the robustness of the PST test, we performed power analyses under the six a priori models. First, we used Mesquite to generate 100 replicate datasets each of which contained 14 gene trees (i.e., “pseudo-observed” gene trees). We then used Mesquite to generate a distribution of *s*-values obtained from 1,000 independent gene trees, simulated under each of the six a priori speciation models, to create a null distribution for each model. For each null distribution, we performed 100 separate PST tests by comparing our simulated 14-loci datasets so that we could calculate the percentage of PST tests that had statistically significant differences between null and pseudo-observed *s*-distributions. The percentage of tests that had *P*-values less than the 0.05 significance level represents the statistical power of that particular test (i.e., probability of not obtaining false-negative results or type II error).

### Extended Bayesian Skyline Plot

To test for population bottlenecks within *U. scoparia* that may be associated with Pleistocene climatic cycles, we used the Extended Bayesian Skyline Plot (EBSP) implemented in BEAST v1.7.5 (Heled and Drummond 2008). Beauti v1.7.5 (Drummond and Rambaut 2007) was used to prepare the input XML file according to the author's recommendations on the BEAST website. We analyzed all available sequences for *U. scoparia*. We ran the Markov chain for 100 million steps, discarding the first 20 million as burn-in. We inspected parameter traces with Tracer v1.5 (Rambaut and Drummond 2009) to assess stationarity and to check for high effective sample sizes (ESSs). The same mutation rates and substitution models from our IM analyses were used to convert the output of this program from units of substitutions into years and individuals.

## Results

### Summary statistics

Our data show that *U. scoparia* has significantly less genetic diversity than the *U. notata* complex by a variety of measures. Before phasing, the highest heterozygous SNP frequency was found in the *U. notata* complex (0.33%), whereas *U. scoparia* populations exhibited lower SNP frequencies ranging from 0.04% to 0.09% (Table 1). Only eight heterozygous indels were detected: five in the *U. notata* complex and three in the southernmost (Colorado River) population of *U. scoparia*. In our phased data (Table 2), the *U. notata* complex had more total unique haplotypes than *U. scoparia* (111 vs. 61) despite having fewer total sampled sequences than *U. scoparia* (246 vs. 916). The mean nucleotide diversity (*π*) of the *U. notata* complex (*π* = 0.468% ± 0.380%) was 3.7 times higher than that of *U. scoparia* (*π* = 0.126% ± 0.148%), and this result was statistically significant as determined by a two-tailed t-test (*P* = 0.006). Most nucleotide diversity within the *U. notata* complex was found in the Algodones Dunes samples (*π* = 0.440% ± 0.368%), despite the fact that samples from this locality were collected in a cluster <2 km^2^. This difference was striking because our sampling was biased toward *U. scoparia*, both in terms of geographic coverage and numbers of individuals sequenced. By contrast, the difference between the Amargosa (*π* = 0.109% ± 0.195%) and Mojave (*π* = 0.094% ± 0.096%) drainages was not significant (*P* = 0.79), nor was there a significant difference between the Mojave and Colorado (*π* = 0.147% ± 0.187%) drainages (*P* = 0.37). No recombination was detected using RDP3. With two exceptions, none of the results of Tajima's D test were significant, indicating that 12 loci met the assumption of neutrality and were consistent with long-term population stability. Although we detected 14 fixed differences between the *U. notata* complex and *U. scoparia*, we did not detect any fixed differences between the Amargosa and Mojave populations.

**Table 1:** Analysis of heterozygous single nucleotide polymorphisms (SNPs) and indels in directly sequenced PCR products (before phasing the haplotypes) by population

Population/Species	Total SNPs	Total Indels	Total BP	SNP Frequency (%)	Indel Frequency (%)
*U. notata* complex	200	5	60,836	0.33	0.0082
*U. scoparia*	144	3	212,114	0.07	0.0014
*U. scoparia* (Colorado)	74	3	81,578	0.09	0.0037
*U. scoparia* (Mojave)	32	0	73,369	0.04	0.0000
*U. scoparia* (Amargosa)	38	0	57,167	0.07	0.0000

**Table 2:** Characteristics of our phased dataset

Locus	L	S	D_*notata*_	D_*scoparia*_	*π*_total_ (%)	*π*_*notata*_ (%)	*π*_Algodones_ (%)	*π*_*scoparia*_ (%)	*π*_Colorado_ (%)	*π*_Mojave_ (%)	*π*_Amargosa_ (%)	k_*notata*_	k_*scoparia*_	n_*notata*_	n_*scoparia*_	F_NS_
BDNF	628	4	−0.098	–	0.159	0.173	0.152	0.000	0.000	0.000	0.000	4	1	20	56	0
RAG-1	705	10	−**1.868**	1.430	0.208	0.057	0.081	0.088	0.103	0.072	0.024	4	5	20	54	2
PNN	600	13	−0.887	−0.804	0.181	0.343	0.393	0.121	0.146	0.039	0.108	6	6	12	54	0
R35	577	13	0.873	0.092	0.387	0.565	0.504	0.155	0.206	0.099	0.000	13	4	18	60	0
Sun07	546	11	−1.640	−1.452	0.211	0.212	0.134	0.013	0.033	0.000	0.000	8	3	20	56	2
Sun08	619	7	–	−0.532	0.193	0.000	0.000	0.069	0.059	0.163	0.054	1	3	2	22	5
Sun10	651	22	−0.589	0.922	0.455	0.665	0.716	0.157	0.127	0.072	0.183	14	6	20	54	1
Sun12	419	9	0.050	−0.137	0.192	0.564	0.524	0.047	0.000	0.056	0.088	12	2	18	56	0
Sun18	379	7	0.022	−0.448	0.630	0.375	0.352	0.062	–	0.080	0.000	3	2	10	16	2
Sun28	571	14	0.252	−1.356	0.484	0.586	0.530	0.154	0.267	0.065	0.077	13	9	20	54	0
Uma03	523	6	−0.329	−0.911	0.131	0.241	0.148	0.007	0.016	0.000	0.000	5	2	20	108	0
Uma05	298	11	1.062	−1.220	0.768	0.774	0.745	0.252	0.225	0.302	0.137	9	5	18	42	0
Uma06	146	11	1.739	−0.132	1.237	1.514	1.428	0.579	0.695	0.289	0.756	8	6	20	142	2
Uma08	384	19	−1.326	−**1.789**	0.219	0.482	0.456	0.065	0.030	0.074	0.100	11	7	28	142	0
Total	7046	157	–	–	–	–	–	–	–	–	–	111	61	246	916	14
Mean	503.29	11.21	–	–	0.390	0.468	0.440	0.126	0.147	0.094	0.109	7.93	4.36	17.57	65.43	1.00
Std Dev	156.80	4.90	–	–	0.312	0.380	0.365	0.148	0.187	0.096	0.195	4.21	2.31	6.09	38.47	1.47

L is the length of the locus in bp, S is the number of segregating sites, D is Tajima's D statistic (statistically significant results are bolded), *π* is nucleotide diversity, k is the number of unique haplotypes, n is the number of sequences per species per locus, and F_NS_ is the number of fixed differences between the *U. notata* complex and *U. scoparia*.

### Gene trees

Our maximum-likelihood gene trees with bootstrap values and Slatkin's *s*-values for all fourteen loci are shown in File S1. Seven loci (50%) have a Slatkin's *s*-value of 1: five loci show reciprocal monophyly between *U. scoparia* and the *U. notata* complex, and two show a monophyletic *U. scoparia* nested within the *U. notata* complex. Two loci have no sharing of alleles but no monophyly, and the remaining five loci have shared alleles between the two species. Our Bayesian 50% majority rule consensus trees with posterior probability values and Slatkin's *s*-values are shown in File S2. These trees were nearly identical to our maximum-likelihood trees, as six loci (43%) had a Slatkin's *s*-value of 1.

### Population structure

For the full dataset, Structurama consistently found the highest support (0.76 posterior probability) for a three-population model (Table 3): one population consisting of *U. scoparia*, one consisting of *U. notata*, and one consisting of *U. inornata + U. rufopunctata*. Excluding *U. inornata* and *U. rufopunctata*, we found the highest support (0.85 posterior probability) for a two-population model, corresponding to *U. scoparia* and *U. notata*. Excluding *U. notata* (*U. scoparia* only), we found the highest support (0.84 posterior probability) for a one-population model.

**Table 3:** Structurama results. Posterior probabilities for each treatment are shown and MML is the mean marginal likelihood

No. of Populations	1	2	3	4	5	6	MML
All Species	0.00	0.00	**0.76**	0.23	0.01	0.00	−115.95
Excluding *U. inornata/U. rufopunctata*	0.00	**0.85**	0.14	0.01	0.00	0.00	−138.59667
*U. scopari*a only	**0.84**	0.15	0.00	0.00	0.00	0.00	−312.41

The most probable number of populations for each treatment is boldfaced.

Geneland results are shown in Table 4. The nonspatial model found strong support (1.0 posterior probability) for only two populations, one consisting of the *U. notata* complex and one consisting of *U. scoparia,* regardless of how many species were included. However, the spatial model was sensitive to how many populations were included in the input matrix. Including all species, the spatial analyses found weak support (0.45 posterior probability) for a three-population model, grouping *U. scoparia* and *U. inornata* as separate clusters and *U. notata* and *U. rufopunctata* combined as the third cluster (Fig. S1). The 50% posterior probability contour of population assignment for *U. scoparia* closely parallels the SAF (compare Fig. S1 to Fig. 2). Excluding *U. inornata* and *U. rufopunctata*, the spatial model found the highest support (0.40 posterior probability) for a two-population model consisting of *U. scoparia* and *U. notata* (Fig. S2). Analyzing *U. scoparia* only with the spatial model revealed two clusters (0.50 posterior probability) not detected in any other analyses, consisting of a northwestern cluster in the Mojave Desert and a southeastern cluster including populations closest to the Colorado River (Fig. S3).

**Table 4:** Summary of 18 Geneland analyses. Posterior probabilities for each treatment are shown (averaged over three runs)

No. of Populations	1	2	3	4	5	6	7	8
Nonspatial, *U. scoparia* only	**1.00**	0.00	0.00	0.00	0.00	0.00	0.00	0.00
Nonspatial, *U. scoparia* and *U. notata*	0.00	**1.00**	0.00	0.00	0.00	0.00	0.00	0.00
Nonspatial, all species	0.00	**1.00**	0.00	0.00	0.00	0.00	0.00	0.00
Spatial, *U. scoparia* only	0.00	**0.50**	0.29	0.13	0.05	0.02	0.01	0.00
Spatial, *U. scoparia* and *U. notata*	0.00	**0.40**	0.35	0.16	0.06	0.02	0.01	0.00
Spatial, all species	0.00	0.16	**0.45**	0.24	0.10	0.03	0.00	0.00

The most probable number of populations is boldfaced for each treatment.

### Isolation-with-migration models

**Figure 4:**
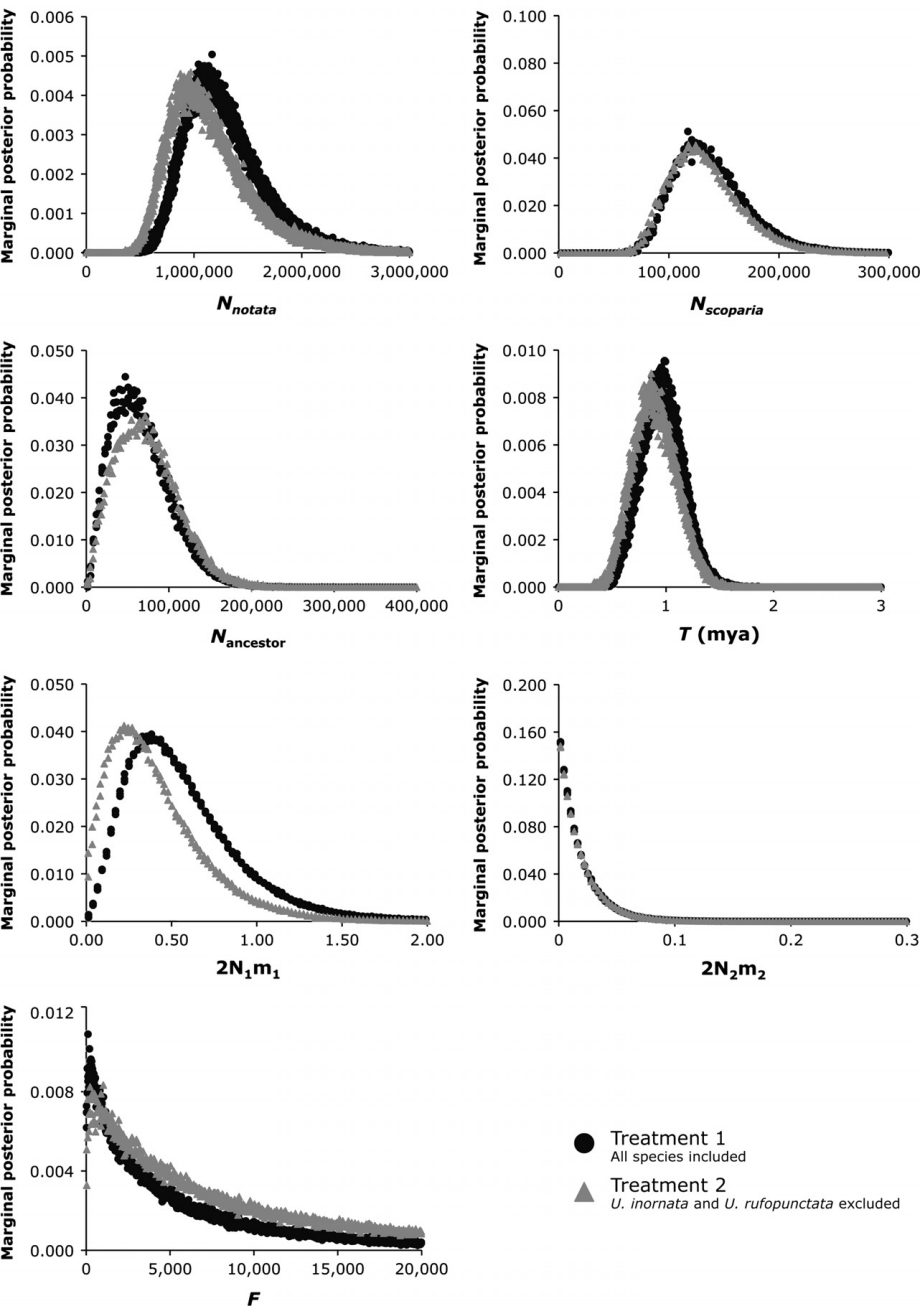
Marginal posterior probability distributions for seven parameters estimated under the IM model under two treatments. Treatment 1 includes all species, while treatment 2 excludes *U. inornata* and *U. rufopunctata*. *N* represents effective population sizes, *T* is population divergence time in millions of years, *F* is the number of founding individuals of *U. scoparia*, and 2Nm represents migrants/generation.

We conducted six IM runs over two treatments to examine the effects of violating the assumption of panmixia (Fig. 4, Table 5). As expected, *N*_*notata*_ was the most sensitive parameter to violations of the panmixia assumption, with high point estimates ranging from 967,321 to 1,090,596 individuals, with broadly overlapping posterior probability distributions. The other estimates were less sensitive to treatment: *N*_*scoparia*_ ranged from 121,955 to 122,227, and *N*_ancestor_ ranged from 43,764 to 65,817. The population divergence time (*T*) was consistent across all treatments (mid-Pleistocene), ranging from 0.88 to 0.99 mya. In both treatments, the 95% confidence intervals fell within the Pleistocene epoch. The splitting parameter was also relatively insensitive to treatment, with the estimated number of founding individuals of *U. scoparia* (*F*) ranging between 241 and 910 individuals (<1.4% of ancestral population size). In both treatments, gene flow was either low or absent (2*Nm* < 0.36).

**Table 5:** Converted demographic parameters of IM analyses for treatments 1–2 (T1–T2), including most probable estimates (high points) and 95% confidence intervals

	*N*_*notata*_	*N*_*scoparia*_	*N*_ancestor_	*T* (mya)	*F*	*2N_1_ m_1_*	*2N_2_ m_2_*
T1 High Point	1,090,596	121,955	43,764	0.99	241	0.356	0.001
T1 95% Low	732,317	85,778	12,255	0.60	25,143	0.119	0.001
T1 95% High	2,221,457	216,489	146,462	1.34	139	1.410	0.069
T2 High Point	967,321	122,227	65,817	0.88	910	0.222	0.001
T2 95% Low	601,189	81,300	14,934	0.54	40,094	0.035	0.001
T2 95% High	2,130,974	212,934	154,305	1.30	252	1.173	0.072

*N* represents effective population sizes, *T* is population divergence time in millions of years, *F* is the number of founding individuals of *U. scoparia*, and 2Nm values represent effective migration rates. All values are averages from the marginal posterior distributions from three runs with different starting seeds.

Although the results of analyzing treatment 1 with IMa were similar to those of IM (Fig. S4), excluding the splitting parameter from the model decreased estimates of *N*_*e*_. The LRT of nested demographic models in IMa rejected all models (*P* < 0.05) except for those that excluded migration parameters or set the effective population size of *U. scoparia* equal to that of the ancestral population (Table 6). The AIC was consistent with the LRT, as the nine nested models rejected by the LRT were also ranked lowest by AIC, and the highest-ranked model (*θ*_*1*_ *θ*_*2*_ *= θ*_*A*_ *m*_*1*_ *= 0 m*_*2*_ *= 0*) excluded both migration parameters and population growth for *U. scoparia*. Furthermore, all nested models rejected by the LRT were ranked lower than the full model by the AIC, while nearly all nested models not rejected by the LRT were ranked higher than the full model by the AIC, with one exception (*θ*_*1*_ *θ*_*2*_ *θ*_*A*_ *m*_*1*_ *= 0* *m*_*2*_). Thus, nested model testing did not support hypotheses of gene flow (either during or after speciation) or population growth within *U. scoparia,* although *N*_*notata*_ was significantly higher than *N*_ancestor_. The IMa comparison between the Amargosa and Mojave River drainages failed to converge on consistent posterior distributions for *T* and *N*_ancestor_, likely due to the lack of polymorphism between these closely related populations, but estimated small population sizes and high rates of gene flow between these populations (Fig. S5).

**Table 6:** Nested demographic models tested with the LRT in IMa, ranked by AIC

Model	k	log(P)	AIC	Δ*_i_*	ML	*w*_*i*_	ER	df	2LLR
*θ*_*1*_ *θ*_*2*_ *= θ*_*A*_ *m*_*1*_ *= 0 m*_*2*_ *= 0*	2	4.89	−5.79	0	1.00	0.26	n/a	3*	3.02
*θ*_*1*_ *θ*_*2*_ *= θ*_*A*_ *m*_*1*_ *= m*_*2*_	3	5.47	−4.95	0.84	0.66	0.17	1.52	2	1.86
*θ*_*1*_ *θ*_*2*_ *θ*_*A*_ *m*_*1*_ *m*_*2*_ *= 0*	4	6.40	−4.81	0.98	0.61	0.16	1.63	1*	0
*θ*_*1*_ *θ*_*2*_ *= θ*_*A*_ *m*_*1*_ *m*_*2*_	4	6.40	−4.81	0.98	0.61	0.16	1.63	1	0
*θ*_*1*_ *θ*_*2*_ *θ*_*A*_ *m*_*1*_ *= 0* *m*_*2*_ *= 0*	3	4.98	−3.96	1.82	0.40	0.10	2.49	2*	2.84
*θ*_*1*_ *θ*_*2*_ *θ*_*A*_ *m*_*1*_ *= m*_*2*_	4	5.52	−3.05	2.74	0.25	0.06	3.94	1	1.76
FULL	5	6.40	−2.81	2.98	0.23	0.06	4.43	n/a	n/a
*θ*_*1*_ *θ*_*2*_ *θ*_*A*_ *m*_*1*_ *= 0* *m*_*2*_	4	4.98	−1.96	3.83	0.15	0.04	6.78	1*	2.85
*θ*_*1*_ *= θ*_*A*_ *θ*_*2*_ *m*_*1*_ *m*_*2*_	4	−4.79	17.58	23.36	0	0	1.18E + 05	1	**22.39**
*θ*_*1*_ *= θ*_*A*_ *θ*_*2*_ *m*_*1*_ *= m*_*2*_	3	−5.94	17.88	23.66	0	0	1.38E + 05	2	**24.69**
*θ*_*1*_ *= θ*_*A*_ *θ*_*2*_ *m*_*1*_ *= 0 m*_*2*_ *= 0*	2	−9.21	22.43	28.22	0	0	1.34E + 06	3*	**31.24**
*θ*_*1*_ *= θ*_*2*_ *θ*_*A*_ *m*_*1*_ *m*_*2*_	4	−118.52	245.04	250.82	0	0	2.92E + 54	1	**249.85**
*θ*_*1*_ *= θ*_*2*_ *θ*_*A*_ *m*_*1*_ *= m*_*2*_	3	−121.38	248.76	254.55	0	0	1.88E + 55	2	**255.57**
*θ*_*1*_ *= θ*_*2*_ *= θ*_*A*_ *m*_*1*_ *m*_*2*_	3	−142.72	291.43	297.22	0	0	3.47E + 64	2	**298.24**
*θ*_*1*_ *= θ*_*2*_ *θ*_*A*_ *m*_*1*_ *= 0 m*_*2*_ *= 0*	2	−144.20	292.40	298.18	0	0	5.62E + 64	3*	**301.21**
*θ*_*1*_ *= θ*_*2*_ *= θ*_*A*_ *m*_*1*_ *= m*_*2*_	2	−148.98	301.96	307.74	0	0	6.69E + 66	3	**310.76**
*θ*_*1*_ *= θ*_*2*_ *= θ*_*A*_ *m*_*1*_ *= 0 m*_*2*_ *= 0*	1	−161.59	325.19	330.97	0	0	7.42E + 71	4*	**336.00**

*θ*_*1*_, *θ*_*2*_, and *θ*_*A*_ represent the effective population sizes for the *U. notata* complex, *U. scoparia*, and the ancestral population, respectively, while *m*_*1*_ and *m*_*2*_ represent migration rates. Shown for each model are the number of parameters (k), the logarithm of the probability for each model, the AIC score, AIC differences from best model (Δ_*i*_), model likelihood (ML), model probabilities (*w*_*i*_), evidence ratio (ER), degrees of freedom (df; an asterisk indicates that the test distribution of 2LLR is a mixture), and the likelihood ratio score (2LLR; boldfaced values indicate rejected models using a chi-squared test, *P* < 0.05). All values were calculated following Hey and Nielsen (2007) and Carstens et al. (2009, 2010).

### Hypothesis testing using coalescent simulations

**Figure 5:**
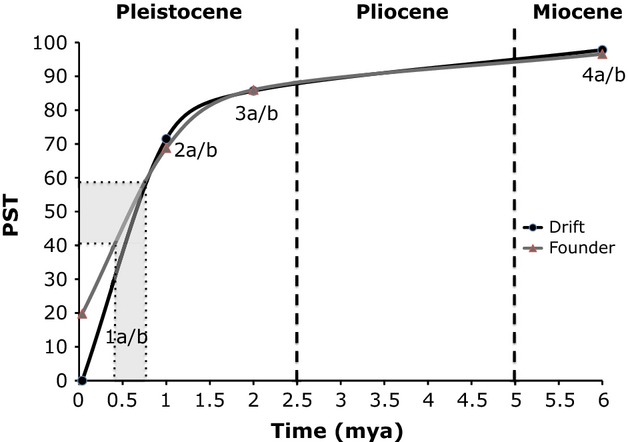
Percentage sorted trees (PST) as a function of time for all eight models tested in this study. The gray box corresponds with a PST interval of 41–59%, representing models that cannot be rejected using the chi-square test (*P* > 0.05). This interval corresponds with late Pleistocene divergence dates of 0.45–0.8 mya for founder models and 0.55–0.8 mya for drift models.

The frequency distributions of Slatkin's *s*-values obtained from our observed and simulated data are shown in Table 7. Based on our observed PST of 50% in our maximum-likelihood trees, our chi-squared test rejected all expected PST values above 59% and below 41% (*P* < 0.05). Likewise, the chi-squared test also rejected all models (*P* < 0.05) using the observed PST value (43%) calculated from the Bayesian consensus trees. Because all simulated models fell outside this distribution, comparisons between our observed PST with those generated from the eight speciation models resulted in rejection of all models, although our observed data are intermediate between models 1a/b and 2a/b. A PST interval of 41–59%, which corresponds with late Pleistocene divergence dates of 0.45–0.8 mya for founder models and 0.55–0.8 mya for drift models, cannot be rejected using the chi-square test (Fig. 5). The founder and drift models are most easily distinguished at recent divergence times with low levels of lineage sorting. Our power analyses reveal that the statistical power of these tests ranges from 47 to 100 depending on which a priori speciation null model is tested – the most recent divergence models (1a/b) showed the highest power, whereas the older models (3a/b, 4a/b) were less powerful (Table S3).

**Table 7:** Frequency distributions (percentages) of Slatkin's *s* for observed data (14 gene trees estimated using RAxML v7.3.0 and MrBayes v3.2.2) and eight simulated datasets of 1,000 trees each (Models 1a-4b)

*s*	Observed (RAxML)	Observed (MrBayes)	1a	1b	2a	2b	3a	3b	4a	4b
**1**	**50.0**	**42.9**	**0.0**	**19.8**	**71.5**	**68.8**	**85.7**	**86.0**	**97.8**	**96.6**
2	28.6	7.1	0.0	12.9	12.5	12.4	4.5	6.0	1.1	1.9
3	7.1	21.4	0.0	9.7	3.9	4.6	4.1	2.1	0.1	0.4
4	7.1	7.1	0.5	11.5	2.9	3.6	1.1	1.1	0.0	0.0
5	0.0	0.0	1.2	9.9	2.0	2.3	1.0	1.0	0.1	0.3
6	0.0	0.0	3.5	8.4	2.6	3.0	0.8	0.7	0.1	0.1
7	7.1	0.0	4.6	5.5	1.3	1.3	0.9	0.5	0.0	0.0
8	0.0	0.0	8.2	5.1	1.4	1.9	0.5	0.9	0.2	0.2
9	0.0	0.0	10.5	6.1	0.6	1.0	0.3	0.6	0.1	0.1
10	0.0	7.1	14.7	4.2	0.6	0.4	0.4	0.4	0.1	0.1
11	0.0	0.0	13.8	2.1	0.2	0.5	0.4	0.3	0.0	0.1
12	0.0	7.1	14.5	2.2	0.3	0.2	0.2	0.2	0.2	0.2
13	0.0	0.0	14.5	1.3	0.2	0.0	0.0	0.1	0.1	0.0
14	0.0	0.0	10.8	0.9	0.0	0.0	0.1	0.1	0.1	0.0
15	0.0	7.1	3.2	0.4	0.0	0.0	0.0	0.0	0.0	0.0

The first bolded row (*s* = 1) corresponds to the percentage sorted trees (PST) for each model, while *s* > 1 indicates incomplete lineage sorting. See Materials and Methods for full details on each model.

### Extended Bayesian Skyline Plot

The EBSP did not show any evidence for population size change through time for *U. scoparia* (Fig. S6). The population remains stable at 114,497 individuals from 3.23 mya to the present (95% confidence interval 63,744–170,662 individuals). Nearly identical results were obtained across multiple runs, and ESS values and parameter trend lines assessed with Tracer indicated that the Markov chain was mixing effectively. Thus, consistent with the results of nested model testing in IMa, this test failed to reject the null hypothesis of no population size change in *U. scoparia* during the Pleistocene.

## Discussion

### *Speciation of* Uma scoparia

The first goal of this study was to test alternative hypotheses regarding the timing and mode of speciation of *U. scoparia* with respect to its sister lineage, the *U. notata* complex. The Neogene vicariance hypothesis (Murphy et al. 2006) predicts that *U. scoparia* diverged from the *U. notata* complex as the result of the development of the lower Colorado River due to Miocene rifting in the Salton Trough, while the Pleistocene dispersal hypotheses (Norris 1958; Adest 1977) predict that *U. scoparia* speciated more recently after dispersing across these topographic barriers, likely in coincidence with climatic fluctuations. Our IM analyses indicate that *U. scoparia* and *U. notata* most likely diverged in the mid-Pleistocene. The 95% confidence intervals include the early Pleistocene (Norris 1958), but exclude the LGM, the late Pleistocene (Adest 1977), the Pliocene, and the Miocene (Murphy et al. 2006). Therefore, the IM model rejected all a priori hypotheses except for that of Norris (1958). This divergence estimate is robust to violations of the IM assumptions (panmixia) as tested with our two data treatments. As little is known about mutation rates of anonymous loci in phrynosomatid lizards, our divergence dates should be interpreted cautiously, yet we argue that our conclusion of Pleistocene speciation is robust to at least a twofold error in our assumed mutation rates – if the assumed rates were halved or doubled, our peak estimate of the divergence time would still fall within the Pleistocene epoch. Such a large discrepancy seems unlikely, as published rates for anonymous loci in tetrapods as distantly related as amphibians, birds, mammals, and lizards range between 2.2 × 10^−9^ and 2.6 × 10^−9^ substitutions/site/yr (Kumar and Subramanian 2002; Lee and Edwards 2008; Townsend et al. 2011; Reilly et al. 2012). Nevertheless, we expect that future comparative genomics studies of lizards will refine these estimates. Our coalescent simulations, which did not rely on a strict molecular clock, also supported a Pleistocene speciation date. Although all of the a priori speciation models we considered were rejected, our observed PST values of 50% (RAxML) and 43% (MrBayes) are both intermediate between the late and early Pleistocene divergence models (1a/b and 2a/b).

Coalescent analyses also ruled out gene flow and population growth of *U. scoparia* during speciation. All 2*Nm* values were <1, and our LRTs of nested demographic models in IMa consistently rejected models with gene flow and population size change of *U. scoparia,* or ranked them lowest under the AIC. The peak estimate of the IM splitting parameter (*s*) indicates that *U. scoparia* was founded by a small number of individuals (<1.4% of the ancestral population), consistent with the dispersal hypothesis of Norris (1958). However, although the posterior distribution of the splitting parameter largely consists of small founder population sizes, the upper end of the 95% credibility interval includes a 50:50 split of the ancestral population. Therefore, this result provides only weak support for a founder event. The findings from our simulation-based hypothesis testing were also ambiguous in this regard, as our PST statistics for constant size and founder models show that older divergence times may not allow the founder and constant size models to be distinguished. However, we also found that our PST test may be a statistically powerful method for detecting founder events for recent divergence dates when lineage sorting is low. We suspect that increasing the number of loci will improve the power of this test, although further simulations are needed to assess this.

Although our coalescent analyses were unable to detect a founder event with high confidence, geological evidence indirectly supports a dispersal-based origin of *U. scoparia*. The mountains associated with the SAF that isolate *U. scoparia* from the *U. notata* complex originated in the Miocene when rifting created the Salton Trough (Elders et al. 1972). Because this barrier is older than our inferred Pleistocene speciation date, we can rule out the Neogene vicariance scenario. Furthermore, dune habitat currently occupied by the *U. notata* complex existed as early as 5 mya, when the Colorado River began to deposit sediment in the rift of the Salton Trough (Buising 1990), whereas the habitat inhabited by *U. scoparia* is largely of Pleistocene age (Enzel et al. 2003; Lancaster and Tchakerian 2003). Thus, based on our Pleistocene divergence estimate and the known geology of this region at the time, we infer that the Colorado River gorge, being the only break in the line of mountains aligned with the SAF, was the most likely dispersal route to the Mojave Desert*,* especially during drought periods that would have exposed sand along the riverbed (Norris 1958).

### Post-speciation demographic history and population structure

The second goal of this study was to test alternative hypotheses concerning intraspecific population structure and post-speciation demographic history of *U. scoparia*. Murphy et al. (2006) found limited mitochondrial structure within northern populations of *U. scoparia*, which led the authors to postulate two distinct population segments in the Amargosa River drainage and Red Pass, despite the observation that the northern (Amargosa) and southern (Mojave/Colorado) mitochondrial haplotypes are sympatric at Red Pass. However, our Geneland and Structurama analyses did not detect geographic structure within *U. scoparia*, with the exception of one Geneland analysis of *U. scoparia* with the spatial model, which detected structure near the Colorado River. However, we discount this result as erroneous because it was not duplicated in the other five Geneland treatments, nor detected in our Structurama analyses. There were no fixed polymorphisms distinguishing the Amargosa River and Mojave River populations, and IMa comparisons failed to converge on a stable posterior probability distribution for the divergence date between these populations. What could account for this apparent incongruence between mtDNA and nDNA data? Due to its maternal inheritance and thus smaller *N*_*e*_, mtDNA will complete lineage sorting faster on average than diploid nDNA and thus is more likely to detect late Pleistocene or Holocene divergence (Zink and Barrowclough 2008). Furthermore, Murphy et al.'s hypothesized divergence time of 0.5 mya is estimated with a single gene tree, and gene divergence is thought to usually predate population divergence (Edwards and Beerli 2000). Other possible explanations for this incongruence include population bottlenecks, although our EBSP failed to find support for this hypothesis, and male-biased dispersal (Toews and Brelsford 2012). Given the lack of geographic structure observed in our data, we reject the hypothesis that the Amargosa River or Red Pass populations represent an ESU. Instead, our analyses indicate that all *U. scoparia* populations comprise a single ESU, consistent with published mtDNA data that demonstrate reciprocal monophyly between *U. scoparia* and the *U. notata* complex (Trepanier and Murphy 2001).

Alternatively, the northern range expansion hypothesis predicts that *U. scoparia* should exhibit low genetic diversity compared with the *U. notata* complex due to a bottleneck event associated with the LGM (Norris 1958). Indeed, the nucleotide diversity of *U. scoparia* is not only low compared with the *U. notata* complex, but is on par with that of mammals such as chimpanzees (Yu et al. 2003) and southern elephant seals (Slade et al. 1998) that are regarded to have low genetic diversity. However, determining the underlying process explaining this pattern proved to be elusive. We utilized the EBSP to test for bottleneck events, but this test did not detect any population size change within *U. scoparia*, nor did nested model testing with IMa. Although we failed to find direct support for a recent demographic expansion, Norris's northward range expansion hypothesis is indirectly supported by the geological history of the Mojave Desert dunes. Throughout most of the Pleistocene, any potential northern habitat in the Amargosa River was inaccessible to *Uma*, as the Mojave River terminated at prehistoric Lake Manix (Enzel et al. 2003). Between 0.013 and 0.014 mya, the natural dam containing Lake Manix burst, creating Afton Canyon (Meek 1989) and allowing the Mojave River to flow to the Amargosa River, establishing a dispersal path for *U*. *scoparia*. This explains the youth of northern Mojave River dunes and why *U. scoparia* are not found in the northern dunes of Death Valley (Norris 1958) and suggests that *U. scoparia* could not have populated the northern part of its contemporary range until the end of the Pleistocene.

## Conclusions

We analyzed fourteen nuclear loci using Bayesian clustering algorithms, nested isolation-with-migration model testing, and novel coalescent simulations to test hypotheses regarding speciation, population structure, and demographic history of *U. scoparia*. We found strong support for Pleistocene divergence without gene flow between *U. scoparia* and *U. notata*, thereby rejecting the Neogene vicariance model of speciation. Instead, the topographic features associated with the SAF must have functioned as a preexisting barrier to dispersal. Although the splitting parameter from our IM results combined with indirect geological evidence provides weak support that *U. scoparia* originated via a founder event, our simulations were unable to differentiate constant size and founder models with high confidence, likely due to the large divergence time relative to effective population size. Nonetheless, our approach offers promise for other study systems that have younger divergence time scenarios. As computational methods advance, genomic data in conjunction with coalescent-based hypothesis testing will likely enable researchers to detect founder events with confidence. Finally, of relevance to the conservation of these species, this study revealed a genetic diversity hot spot in the Algodones Dunes population of *U. notata* (which contains more than three times the genetic diversity observed across all *U. scoparia* populations) and that *U. scoparia* consists of a single ESU.

## Data Accessibility

All data necessary to replicate the analyses of this study have been archived in the Dryad Digital Repository: http://doi.org/10.5061/dryad.9qt14.

## Supporting Information

**Figure S1.** Geneland maps including all species, with individual pixels assigned to population clusters. The contour lines depict posterior probability of population assignment. The lowest posterior probabilities are in red and the highest are in white.

**Figure S2.** Geneland maps excluding *U. inornata* and *U. rufopunctata*, with individual pixels assigned to population clusters. The contour lines depict posterior probability of population assignment. The lowest posterior probabilities are in red and the highest are in white.

**Figure S3.** Geneland maps for *U. scoparia* only, with individual pixels assigned to population clusters. The contour lines depict posterior probability of population assignment. The lowest posterior probabilities are in red and the highest are in white.

**Figure S4.** Marginal posterior probability distributions for six parameters estimated under IMa, comparing *U. scoparia* to the *U. notata* complex.

**Figure S5.** Marginal posterior probability distributions for six parameters estimated under IMa, comparing the Amargosa River and Mojave River populations of *U. scoparia*.

**Figure S6.** Extended Bayesian Skyline Plot (EBSP) for *U. scoparia*, showing effective population size through time.

**File S1.** Maximum-likelihood gene trees estimated with RAxML v7.3.0 (Stamatakis 2006) for all fourteen loci with bootstrap values mapped onto nodes. *U. scoparia* are shown in black and the *U. notata* complex in red. Slatkin's *s*-values are shown for each locus.

**File S2**. Bayesian 50% majority rule consensus trees, estimated with MrBayes v3.2.2 (Ronquist et al. 2012), for all fourteen loci with posterior probabilities mapped onto nodes. *U. scoparia* are shown in black and the *U. notata* complex in red. Slatkin's *s*-values are shown for each locus.

**Table S1.** Sampling matrix with individual lizard information and GPS coordinates for all sequences used in this study. The numeral 1 denotes the presence of sequence data for that individual at that locus.

**Table S2.** PCR primers, annealing temperatures, and literature sources used in this study. PCR reactions consisted of a denaturation step (94° C for 1 min), an annealing step (1 min), and an extension step (72° C for 1 min), repeated 35 times.

**Table S3.** Results of power analyses for each of the six a priori speciation models. The program Mesquite was used to generate 100 simulated 14-locus datasets with characteristics similar to the observed data including an *U. scoparia*/*U. notata* divergence time of 0.5 Ma. These “pseudo-observed” datasets were then individually compared to each null distribution via PST tests (see File S1, S2 for details). The percentage of tests yielding false negative results (Type II error) is shown in the first column, whereas the percentage of tests in which the null hypothesis was correctly rejected (statistical power) is shown in the second column.
