# Supplementary figures and images for: Speciation, population structure, and demographic history of the Mojave Fringe-toed Lizard (Uma scoparia), a species of conservation concern
Source: Ecol Evol. 2014 May 24;4(12):2546–62. doi: 10.1002/ece3.1111 (PMC4203297; doi:10.1002/ece3.1111)

Cluster 1: *U. scoparia*

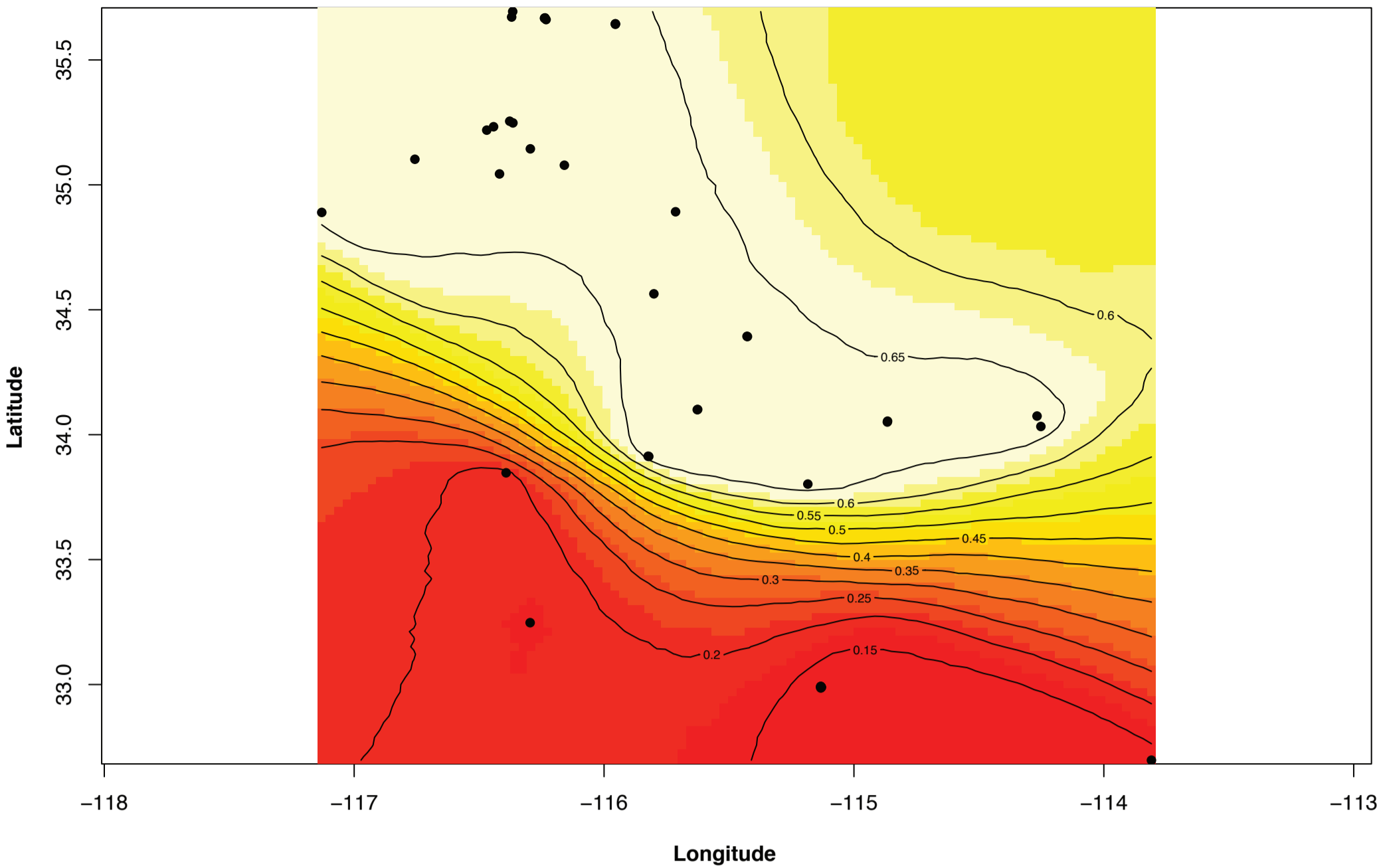

Cluster 2: *U. inornata*

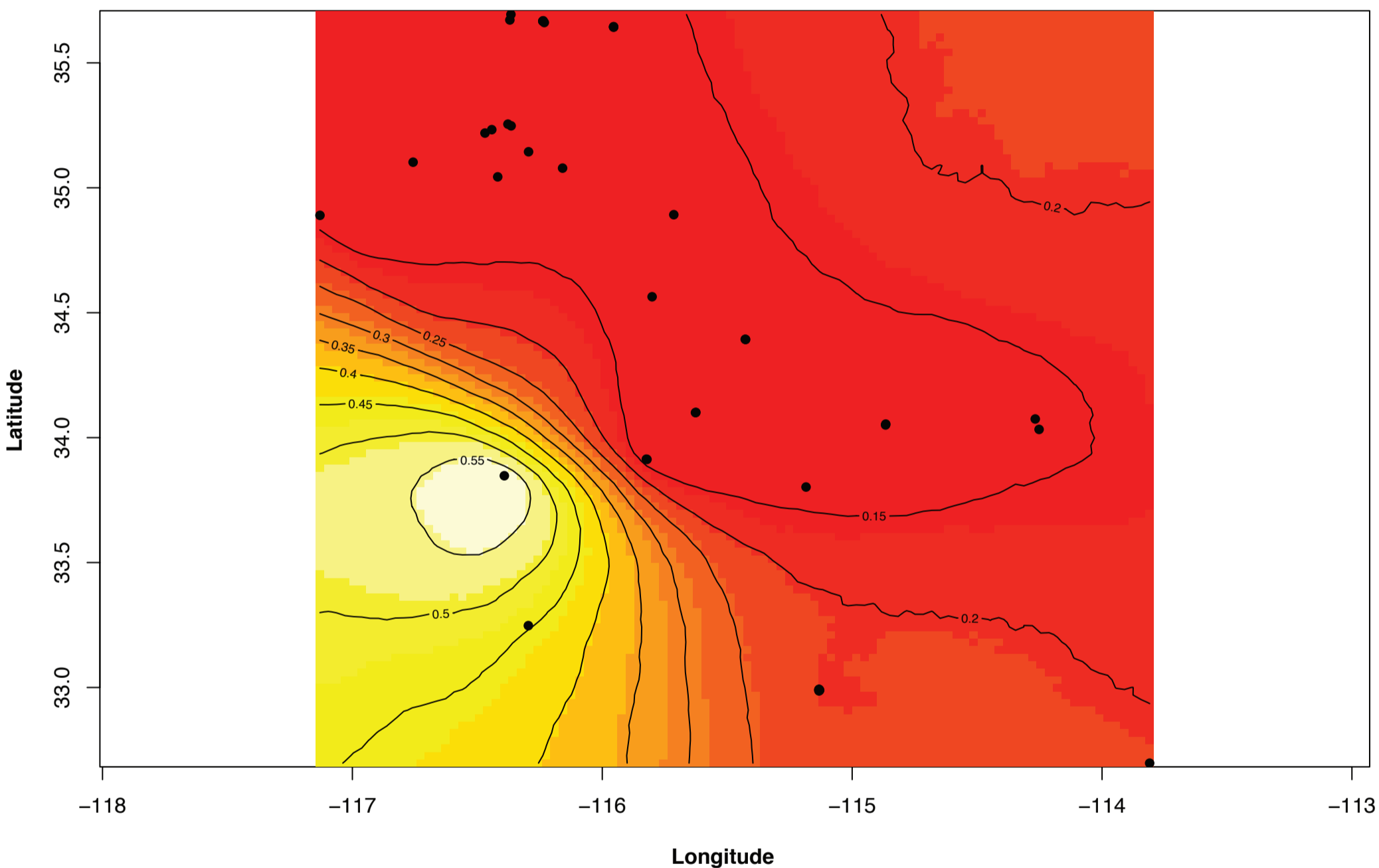

Cluster 3: *U. notata* + *U. rufopunctata*

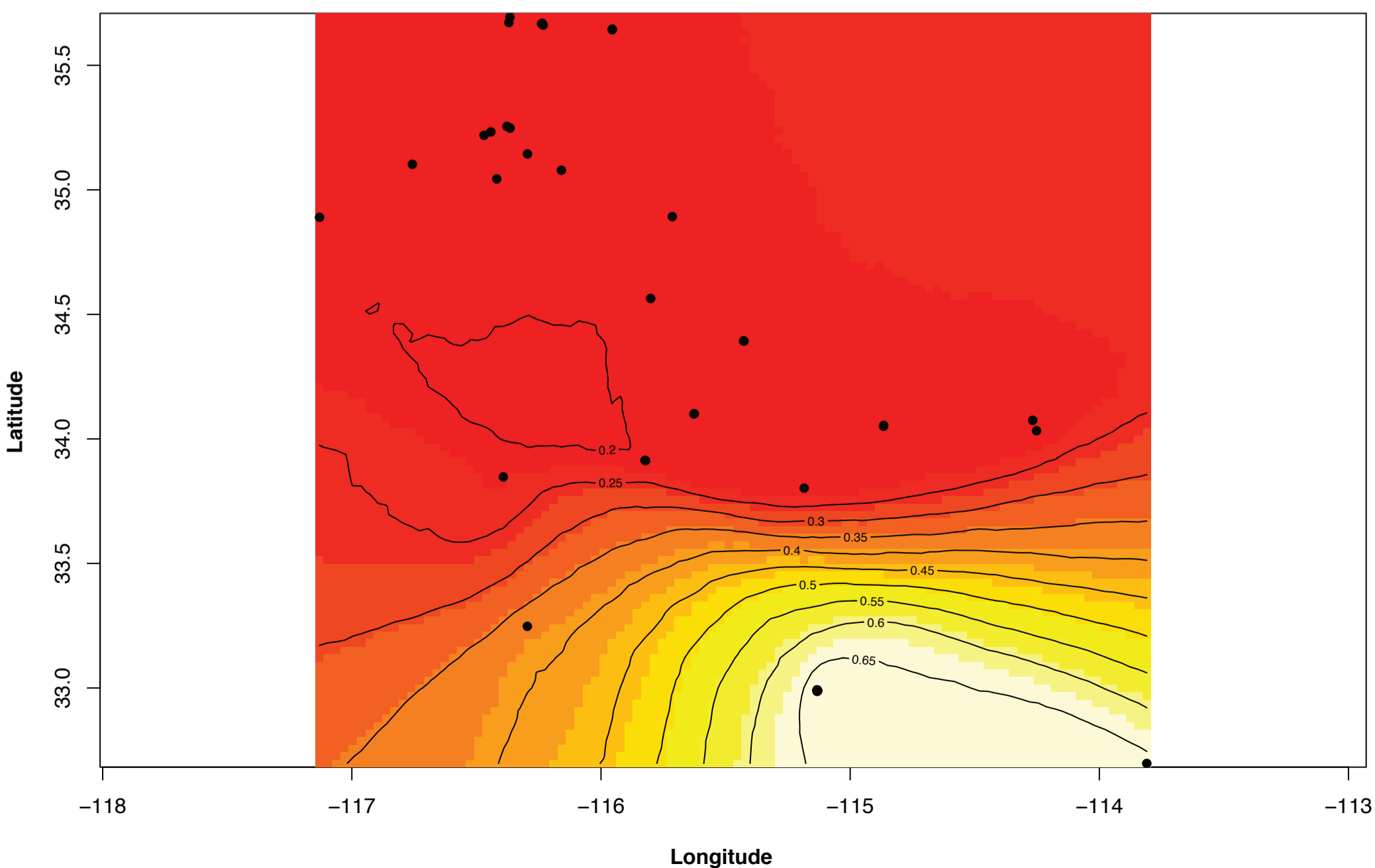

Supplement: Supplementary file 1 — Figure S1. Geneland maps including all species, with individual pixels assigned to population clusters. The contour lines depict posterior probability of population assignment. The lowest posterior probabilities are in red and the highest are in white. [file ece30004-2546-sd1.pdf]

**Cluster 1: *U. scoparia***

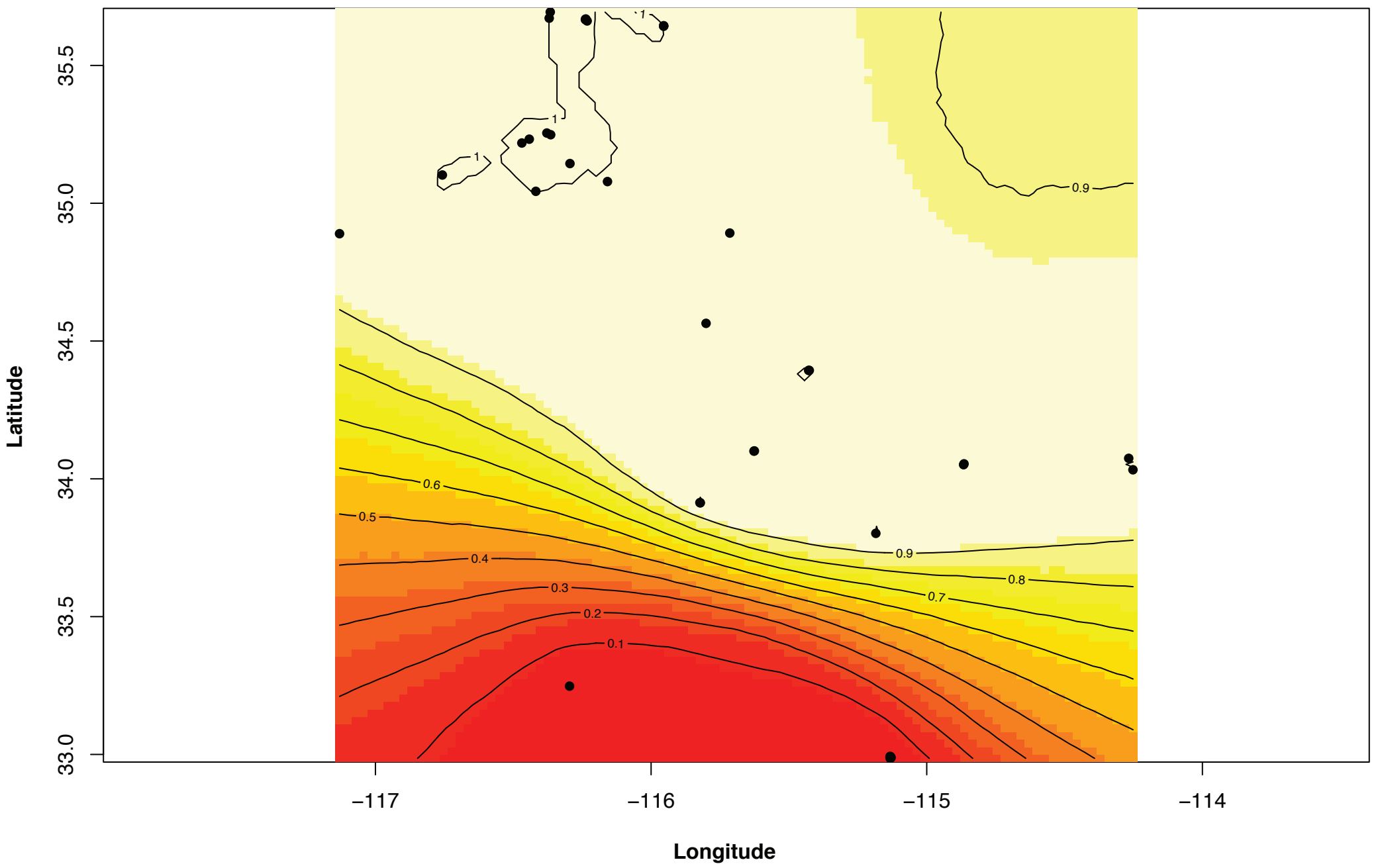

**Cluster 2: *U. notata***

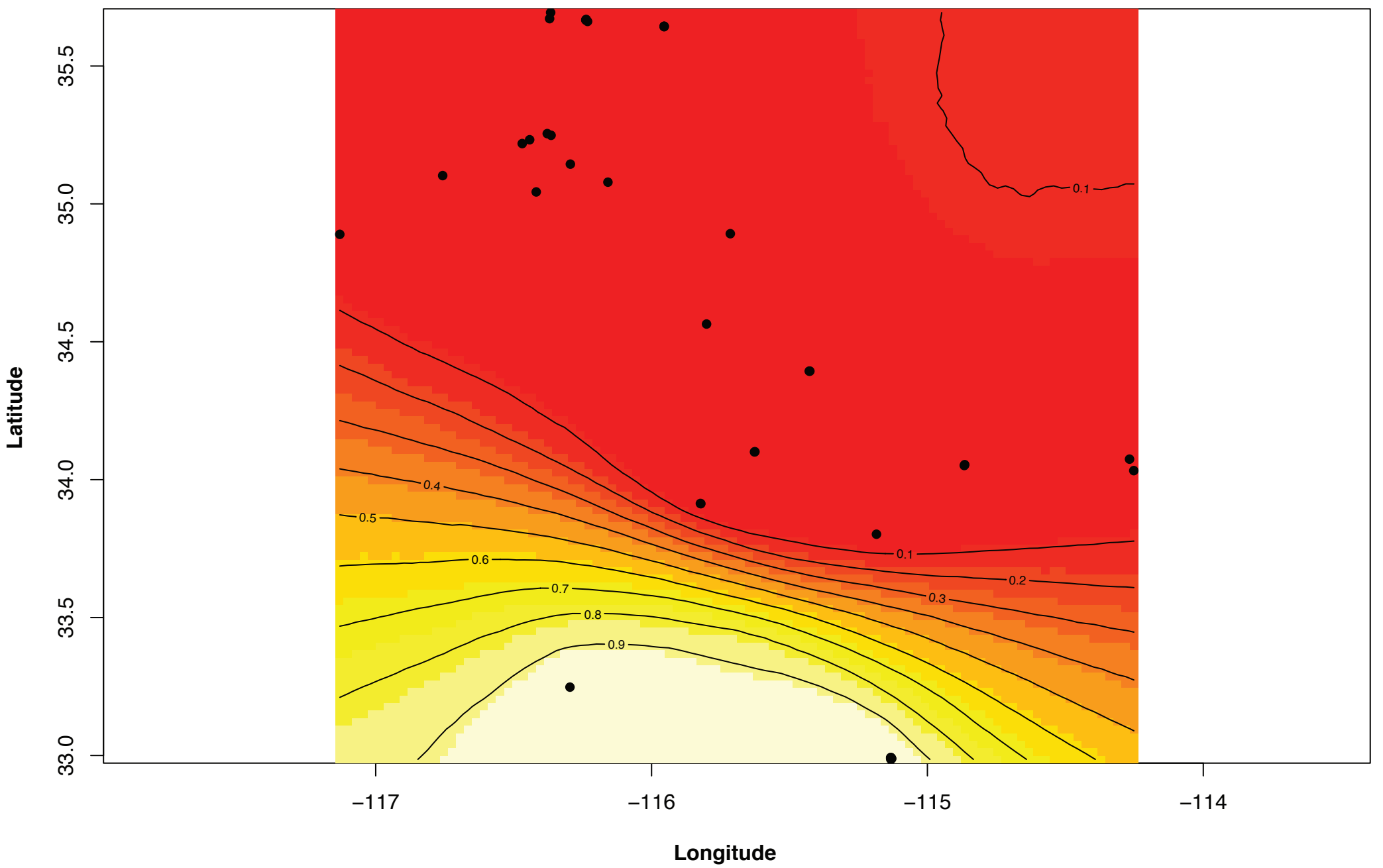

Supplement: Supplementary file 2 — Figure S2. Geneland maps excluding U. inornata and U. rufopunctata, with individual pixels assigned to population clusters. The contour lines depict posterior probability of population assignment. The lowest posterior probabilities are in red and the highest are in white. [file ece30004-2546-sd2.pdf]

Cluster 1: Northwestern *U. scoparia*

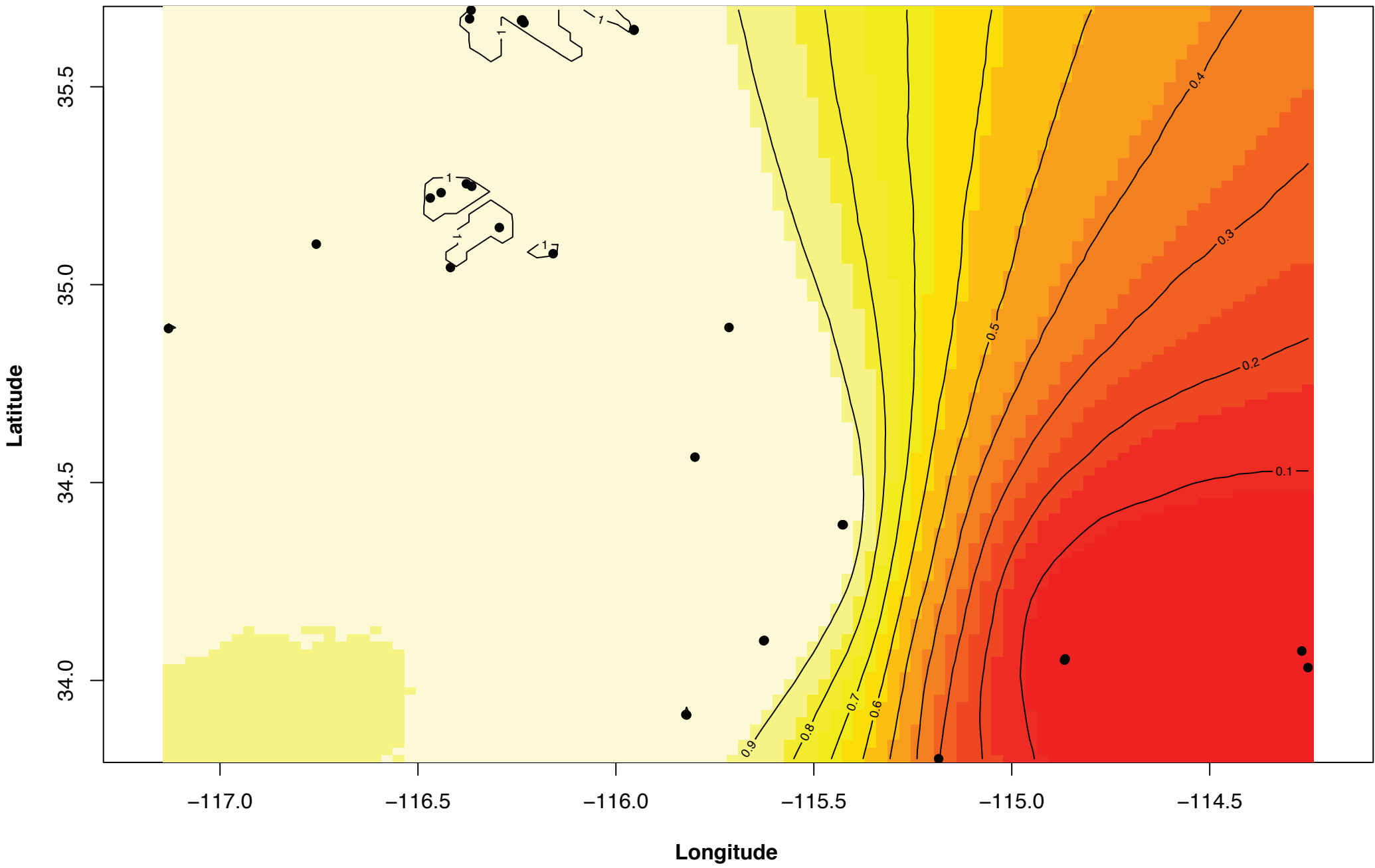

Cluster 2: Southeastern *U. scoparia*

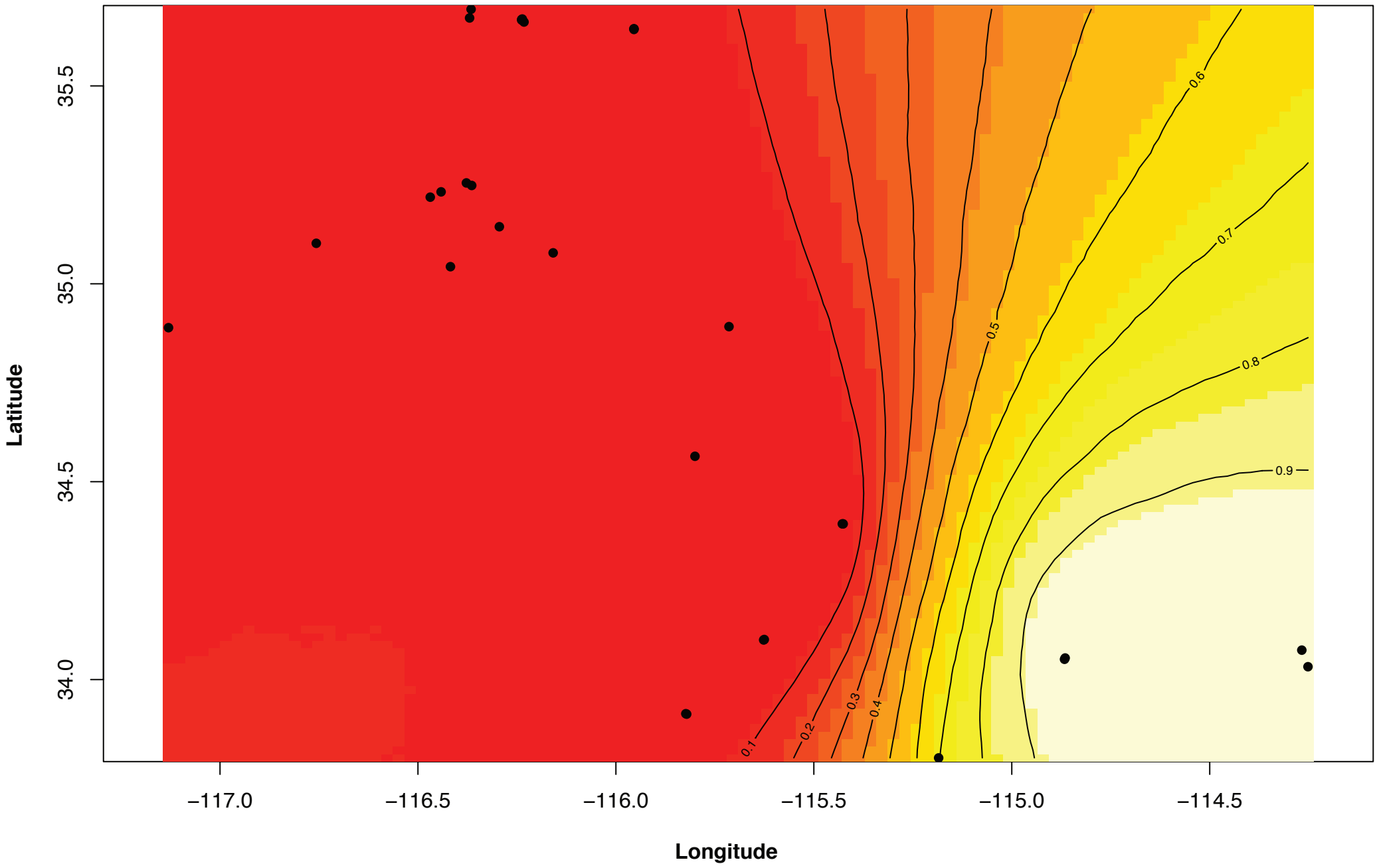

Supplement: Supplementary file 3 — Figure S3. Geneland maps for U. scoparia only, with individual pixels assigned to population clusters. The contour lines depict posterior probability of population assignment. The lowest posterior probabilities are in red and the highest are in white. [file ece30004-2546-sd3.pdf]

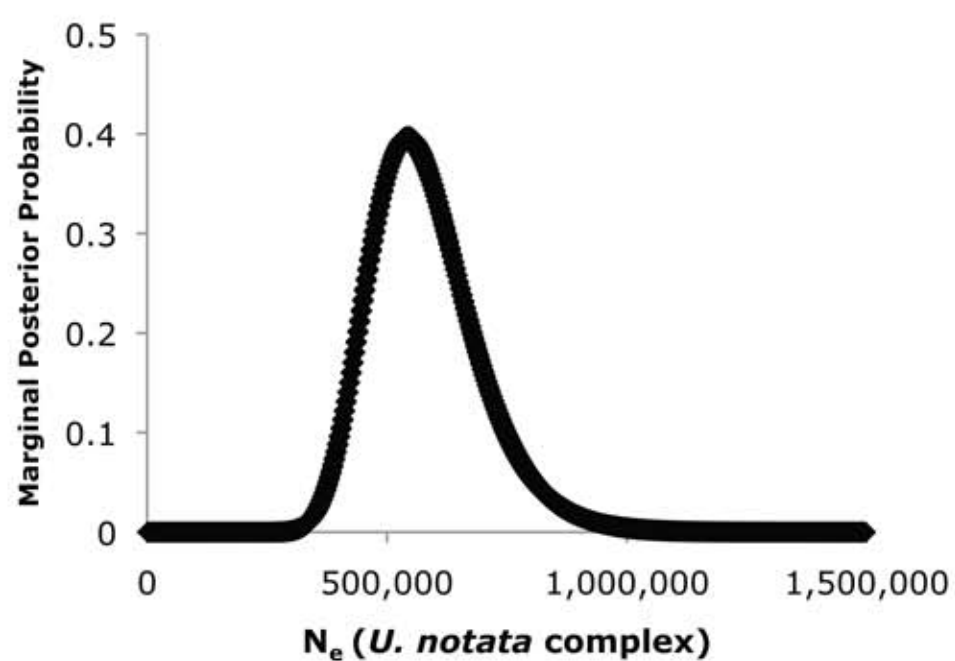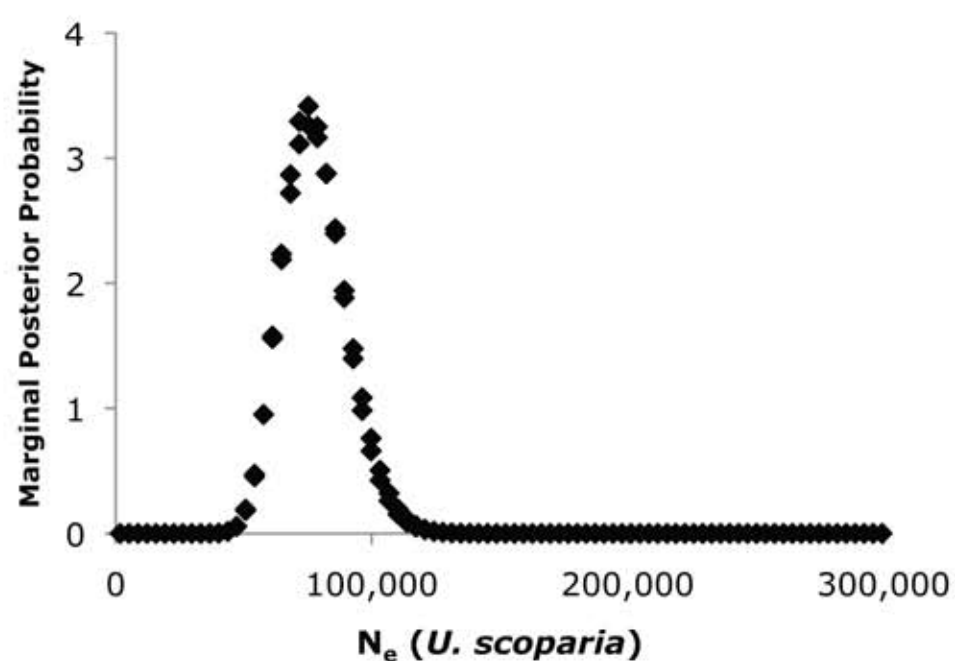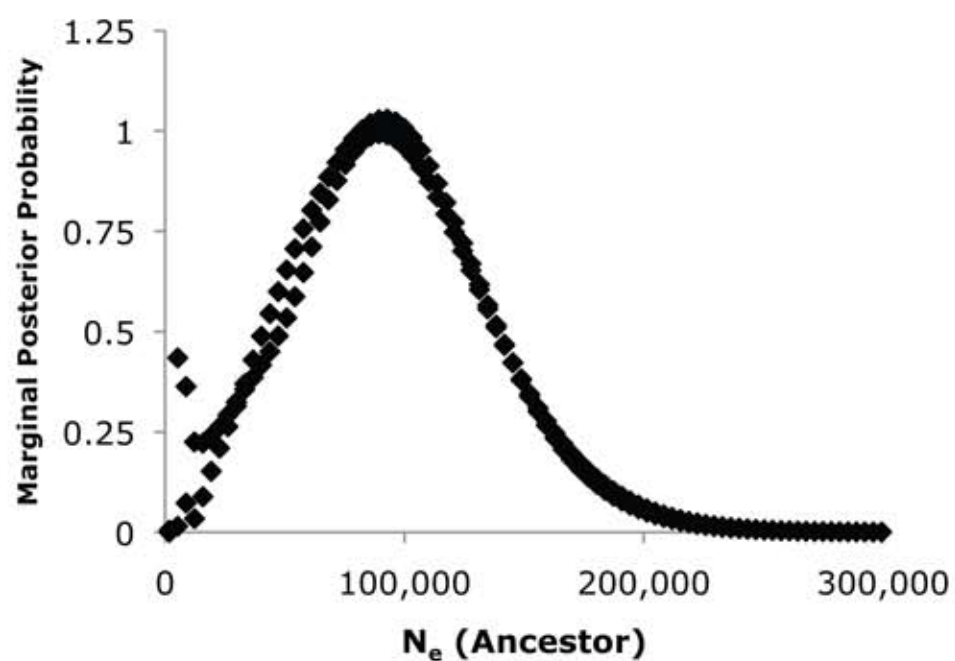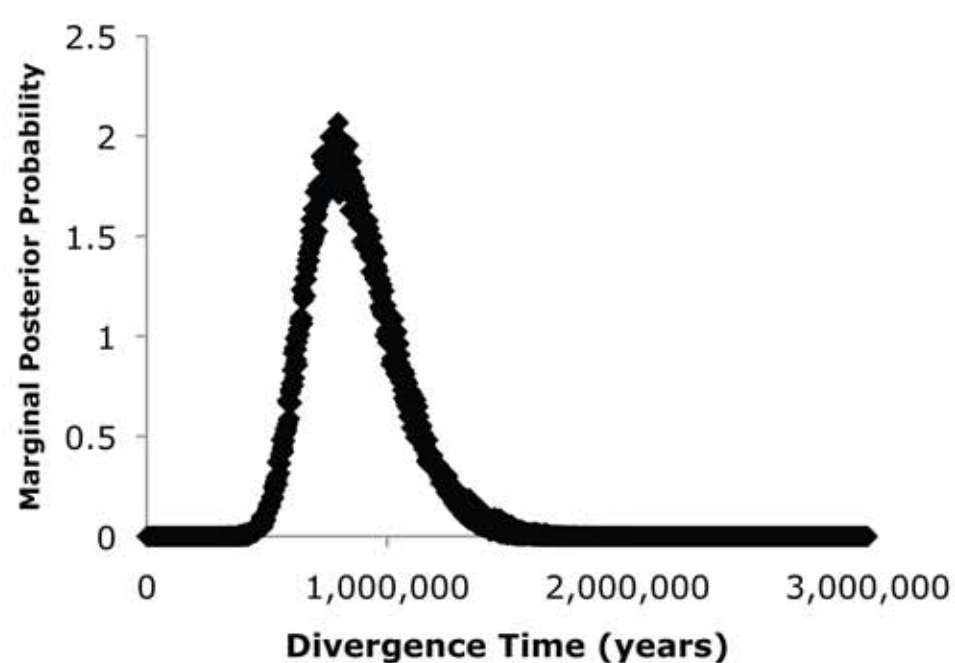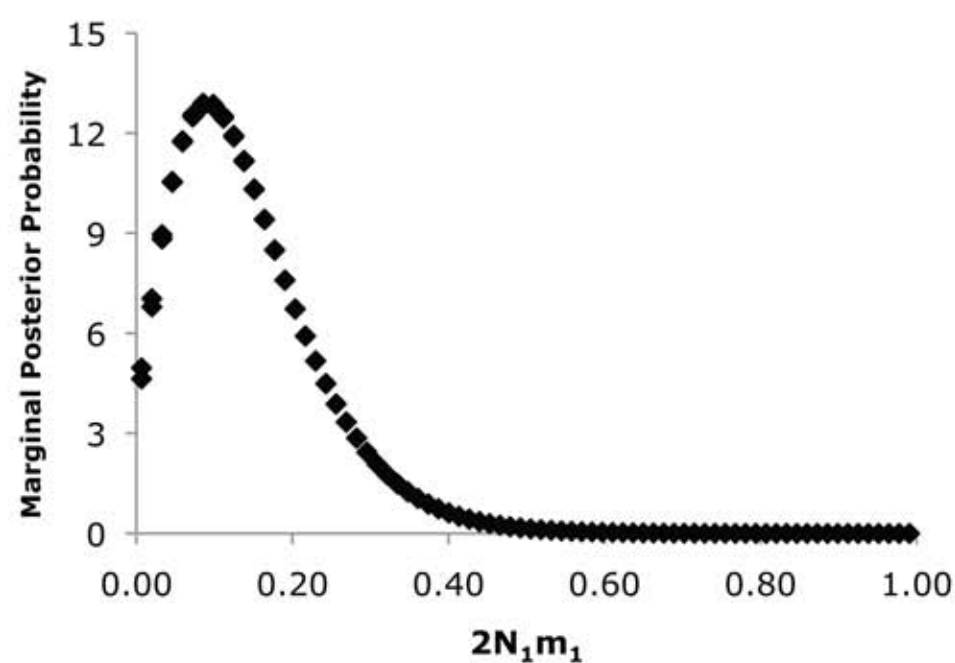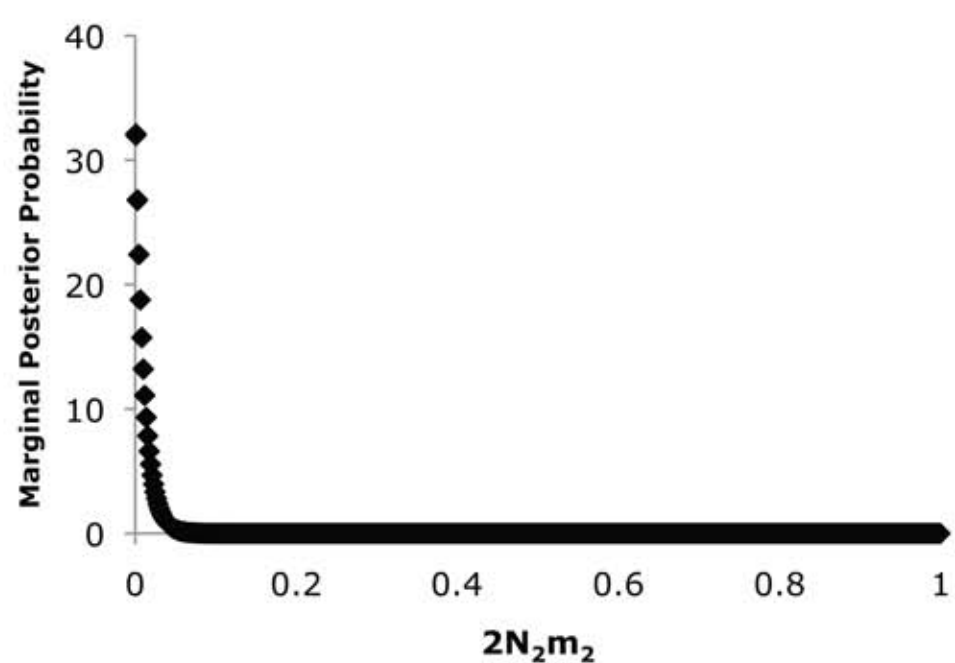

Supplement: Supplementary file 4 — Figure S4. Marginal posterior probability distributions for six parameters estimated under IMa, comparing U. scoparia to the U. notata complex. [file ece30004-2546-sd4.pdf]

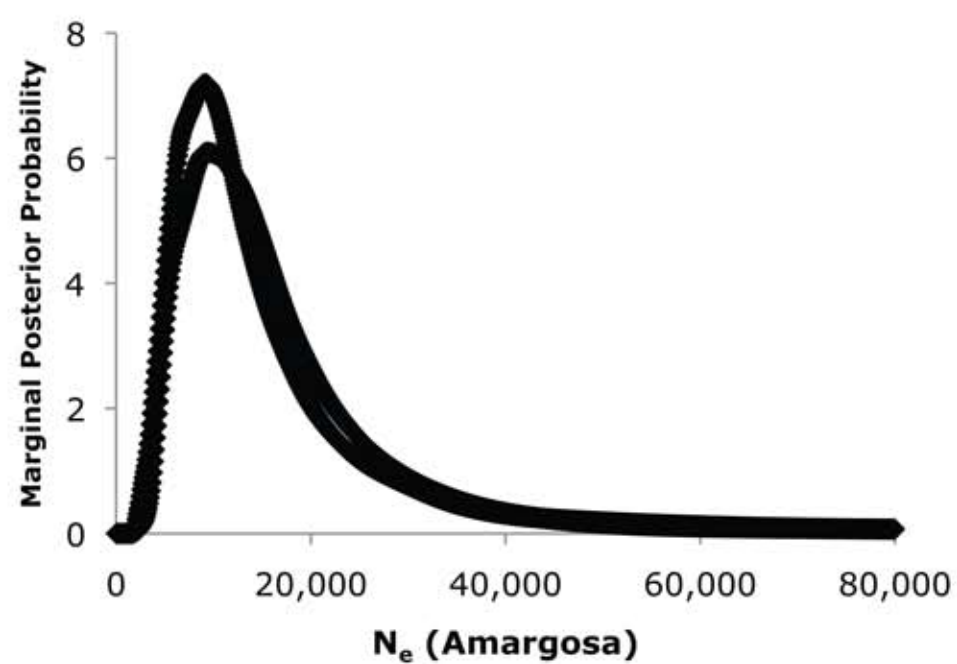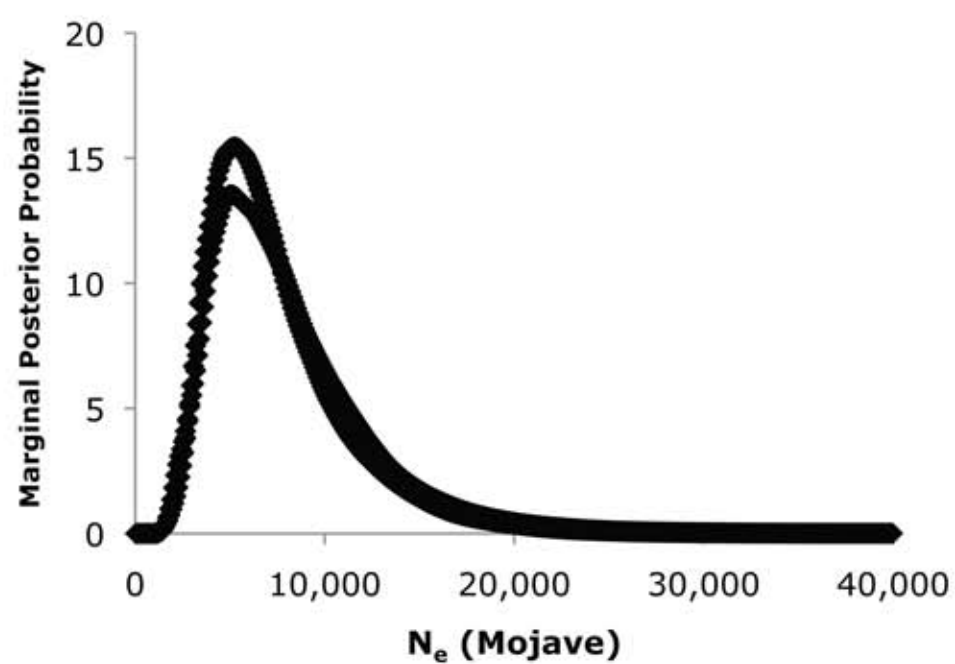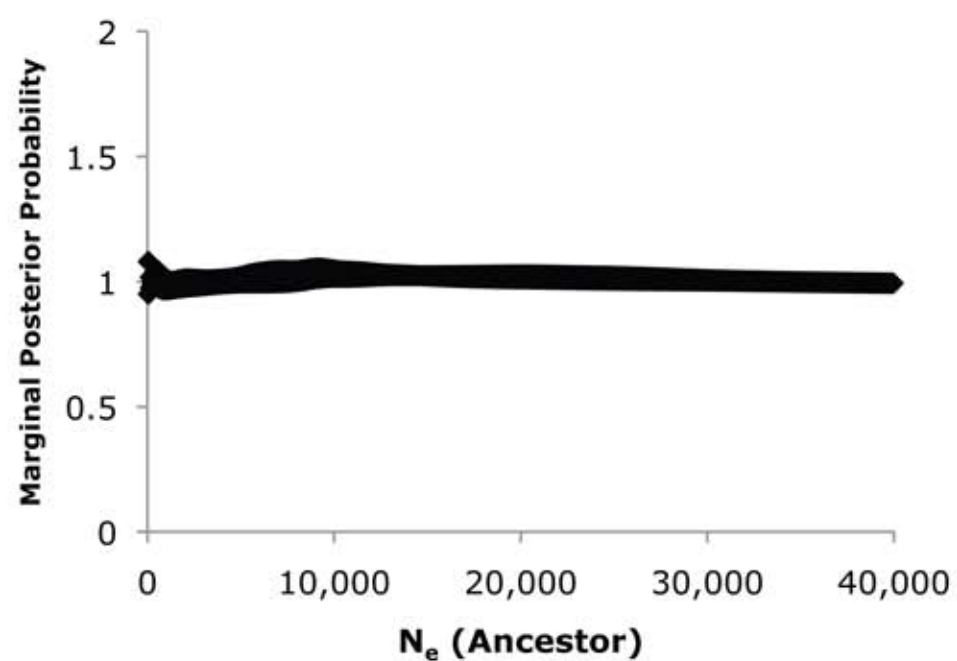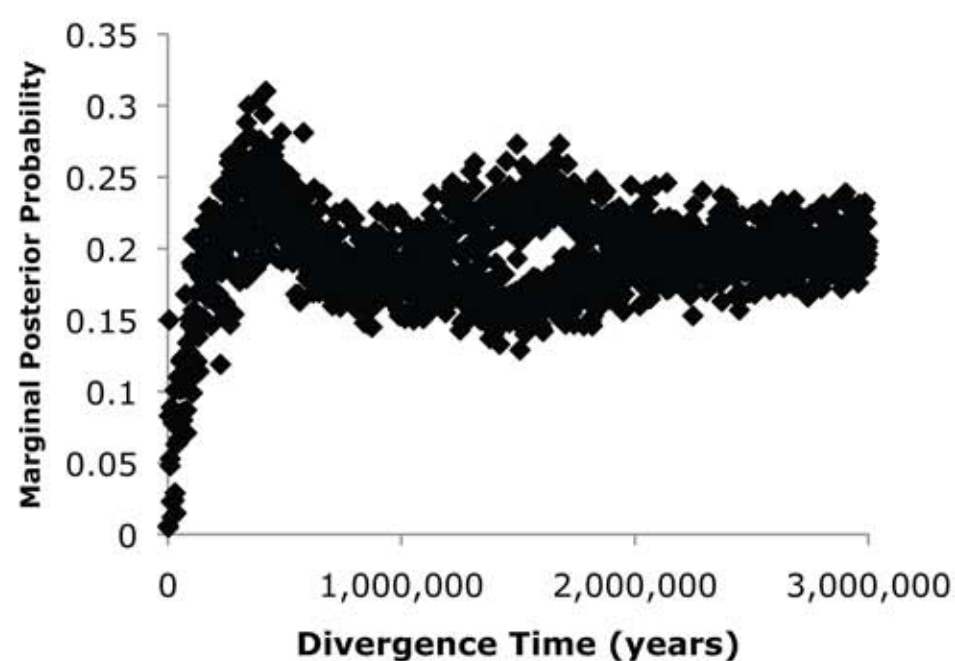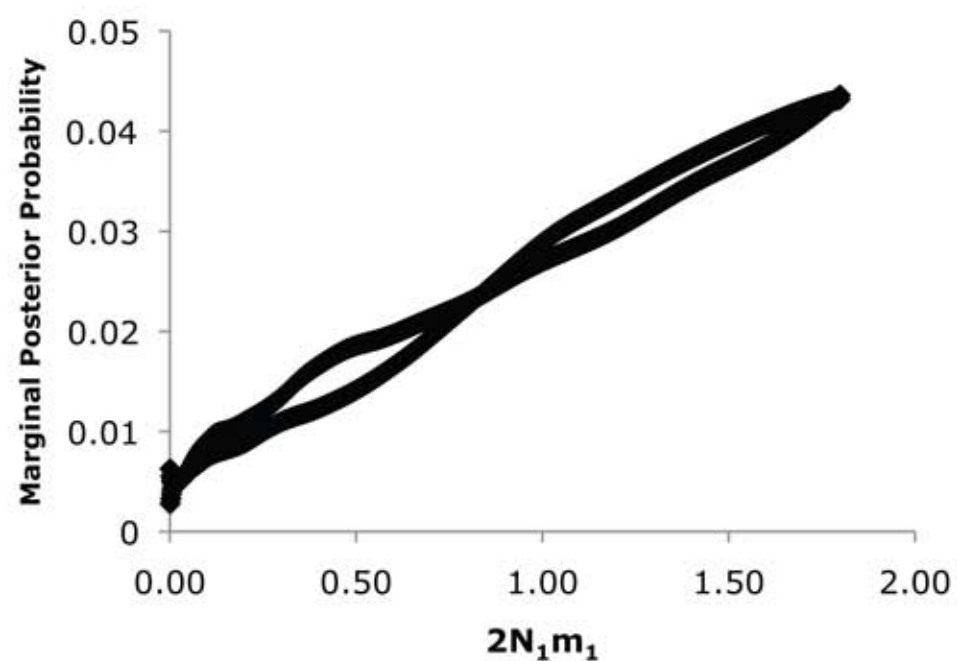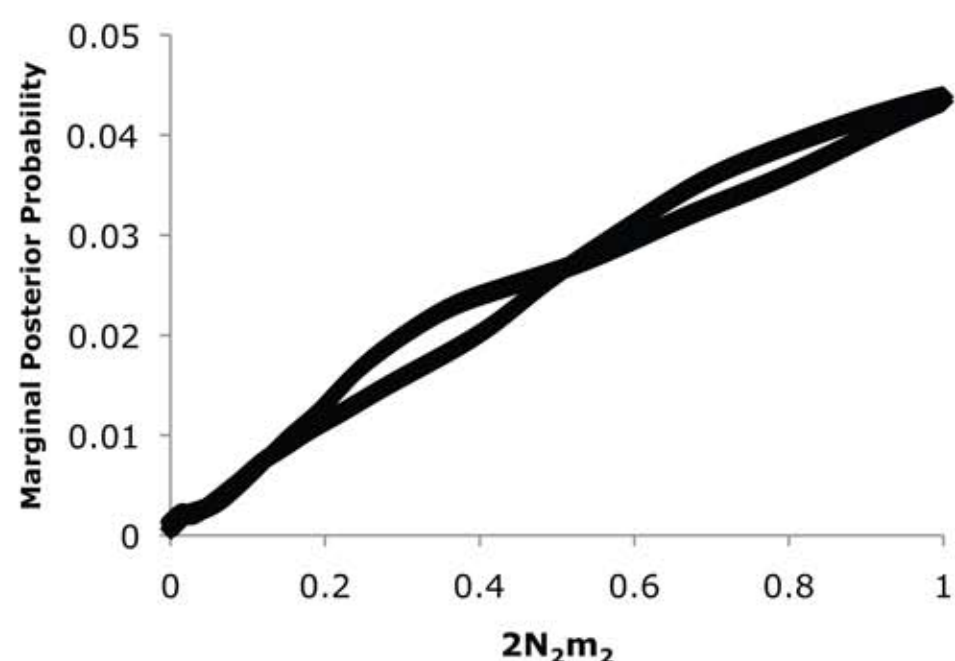

Supplement: Supplementary file 5 — Figure S5. Marginal posterior probability distributions for six parameters estimated under IMa, comparing the Amargosa River and Mojave River populations of U. scoparia. [file ece30004-2546-sd5.pdf]

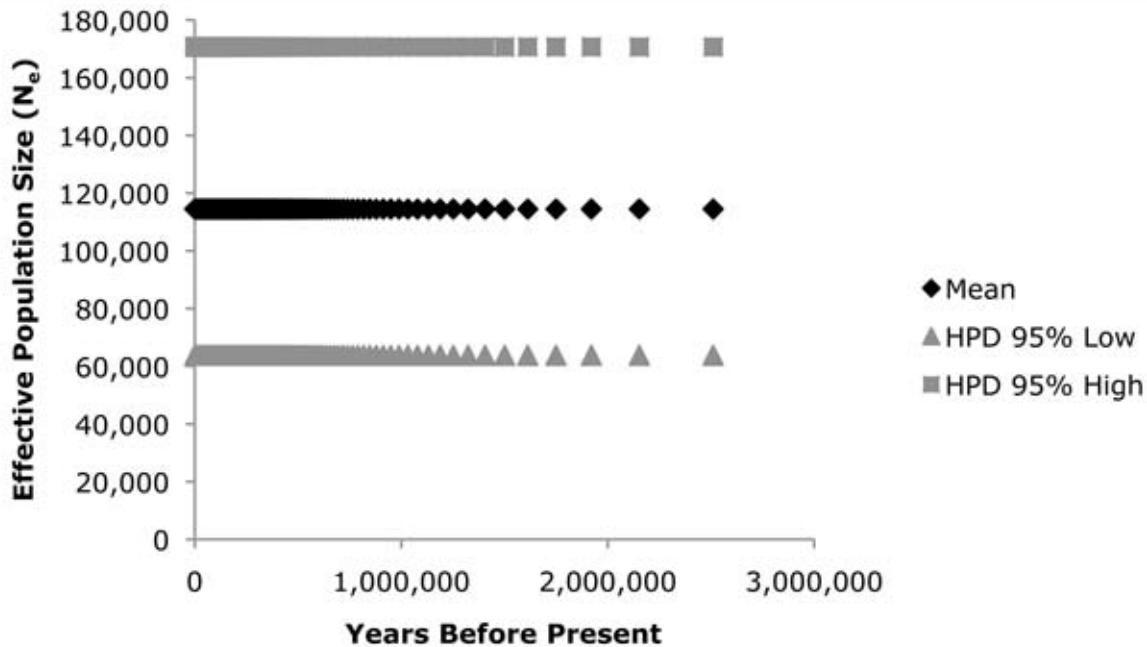

Supplement: Supplementary file 6 — Figure S6. Extended Bayesian Skyline Plot (EBSP) for U. scoparia, showing effective population size through time. [file ece30004-2546-sd6.pdf]
