# Supplementary material for: Speciation, population structure, and demographic history of the Mojave Fringe-toed Lizard (Uma scoparia), a species of conservation concern
Source: Ecol Evol. 2014 May 24;4(12):2546–62. doi: 10.1002/ece3.1111 (PMC4203297; doi:10.1002/ece3.1111)
Supplement: Supplementary file 7 — File S1. Maximum-likelihood gene trees estimated with RAxML v7.3.0 (Stamatakis 2006) for all fourteen loci with bootstrap values mapped onto nodes. U. scoparia are shown in black and the U. notata complex in red. Slatkin's s-values are shown for each locus. [file ece30004-2546-sd7.pdf]

Supplemental File 1. Maximum-likelihood gene trees estimated with RAxML v7.3.0 (Stamatakis 2006) for all fourteen loci with bootstrap values mapped onto nodes. *U. scoparia* are shown in black and the *U. notata* complex in red. Slatkin's *s* values are shown for each locus.

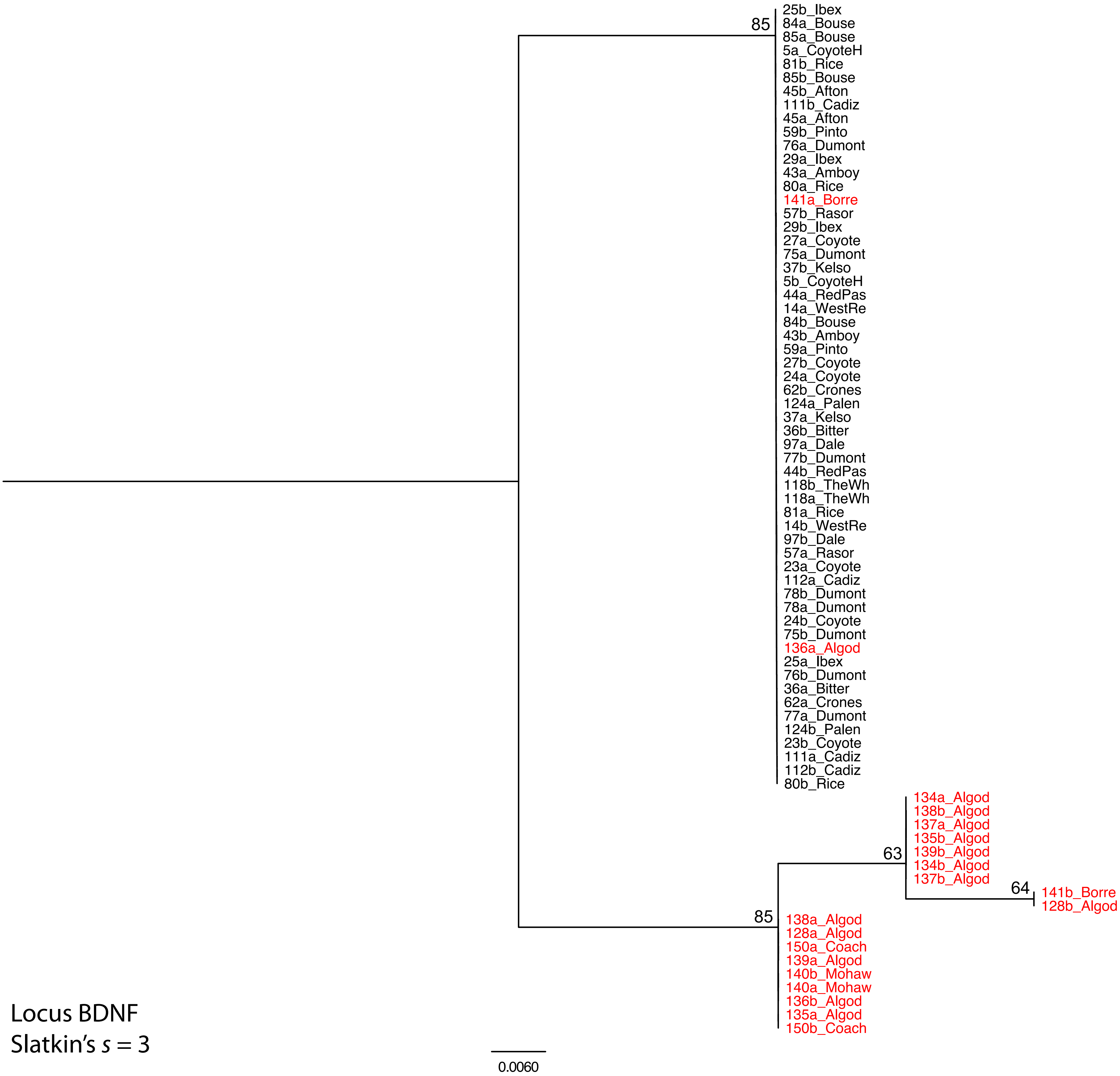
85
25b_Ibex
84a_Bouse
85a_Bouse
5a_CoyoteH
81b_Rice
85b_Bouse
45b_Afton
111b_Cadiz
45a_Afton
59b_Pinto
76a_Dumont
29a_Ibex
43a_Amboy
80a_Rice
141a_Borre
57b_Rasor
29b_Ibex
27a_Coyote
75a_Dumont
37b_Kelso
5b_CoyoteH
44a_RedPas
14a_WestRe
84b_Bouse
43b_Amboy
59a_Pinto
27b_Coyote
24a_Coyote
62b_Crones
124a_Palen
37a_Kelso
36b_Bitter
97a_Dale
77b_Dumont
44b_RedPas
118b_TheWh
118a_TheWh
81a_Rice
14b_WestRe
97b_Dale
57a_Rasor
23a_Coyote
112a_Cadiz
78b_Dumont
78a_Dumont
24b_Coyote
75b_Dumont
136a_Algod
25a_Ibex
76b_Dumont
36a_Bitter
62a_Crones
77a_Dumont
124b_Palen
23b_Coyote
111a_Cadiz
112b_Cadiz
80b_Rice
134a_Algod
138b_Algod
137a_Algod
135b_Algod
139b_Algod
134b_Algod
137b_Algod
63
64
141b_Borre
128b_Algod
85
138a_Algod
128a_Algod
150a_Coach
139a_Algod
140b_Mohaw
140a_Mohaw
136b_Algod
135a_Algod
150b_Coach
Locus BDNF
Slatkin's s = 3
0.0060

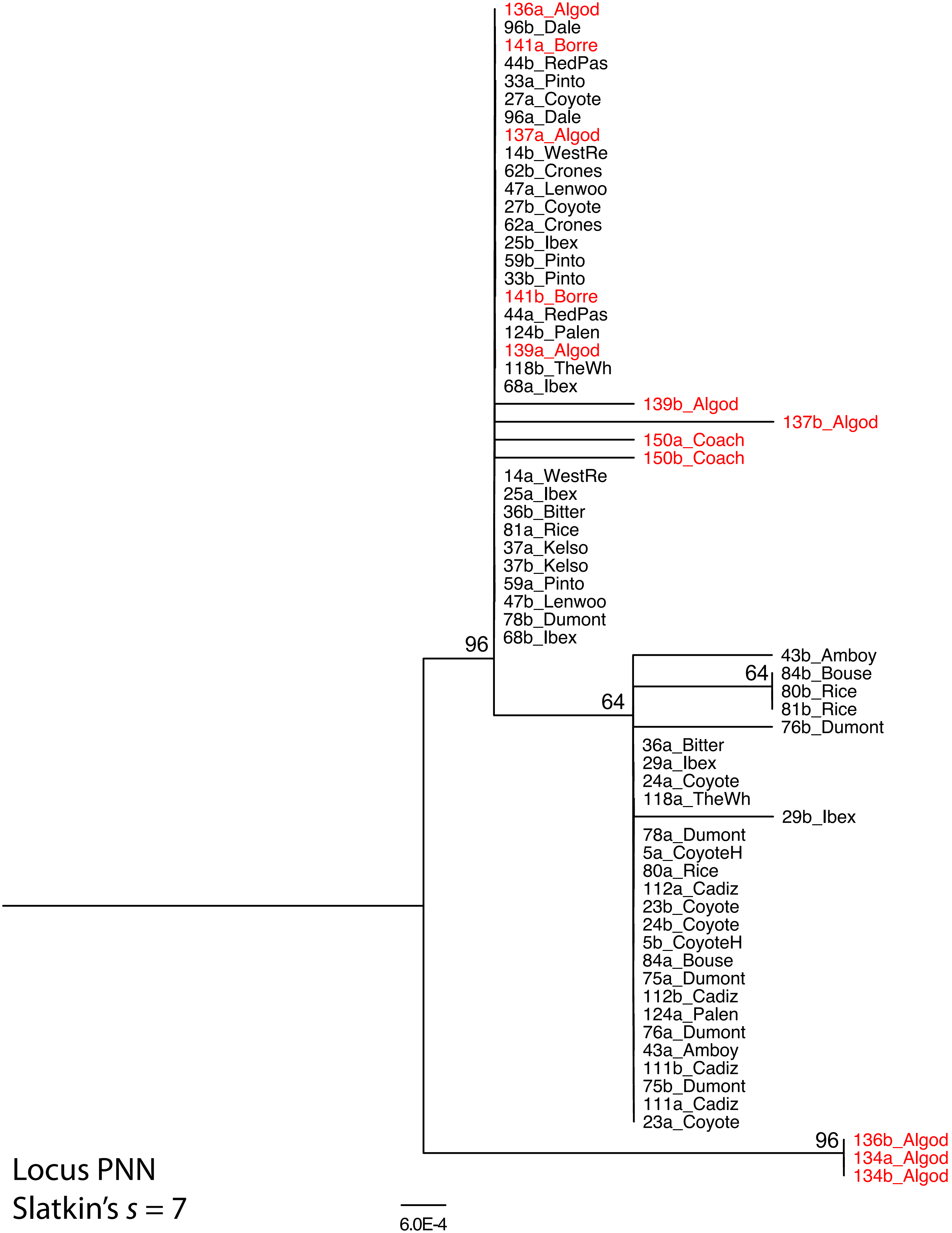
136a_Algod
96b_Dale
141a_Borre
44b_RedPas
33a_Pinto
27a_Coyote
96a_Dale
137a_Algod
14b_WestRe
62b_Crones
47a_Lenwoo
27b_Coyote
62a_Crones
25b_Ibex
59b_Pinto
33b_Pinto
141b_Borre
44a_RedPas
124b_Palen
139a_Algod
118b_TheWh
68a_Ibex
139b_Algod
137b_Algod
150a_Coach
150b_Coach
14a_WestRe
25a_Ibex
36b_Bitter
81a_Rice
37a_Kelso
37b_Kelso
59a_Pinto
47b_Lenwoo
78b_Dumont
68b_Ibex
96
43b_Amboy
64
84b_Bouse
80b_Rice
81b_Rice
64
76b_Dumont
36a_Bitter
29a_Ibex
24a_Coyote
118a_TheWh
29b_Ibex
78a_Dumont
5a_CoyoteH
80a_Rice
112a_Cadiz
23b_Coyote
24b_Coyote
5b_CoyoteH
84a_Bouse
75a_Dumont
112b_Cadiz
124a_Palen
76a_Dumont
43a_Amboy
111b_Cadiz
75b_Dumont
111a_Cadiz
23a_Coyote
96
136b_Algod
134a_Algod
134b_Algod
Locus PNN
Slatkin's s = 7
6.0E-4

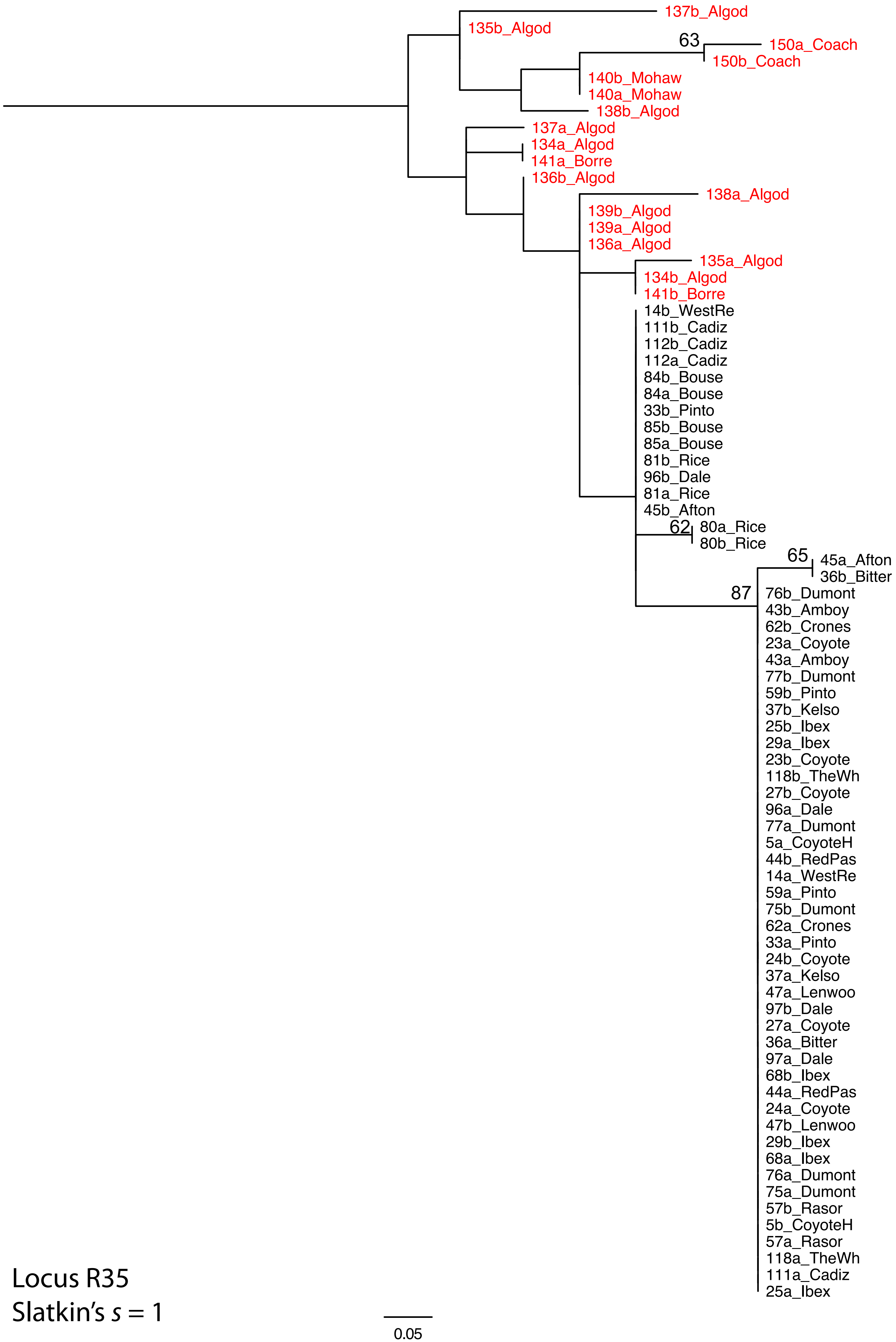
137b_Algod
135b_Algod
63
150a_Coach
150b_Coach
140b_Mohaw
140a_Mohaw
138b_Algod
137a_Algod
134a_Algod
141a_Borre
136b_Algod
138a_Algod
139b_Algod
139a_Algod
136a_Algod
135a_Algod
134b_Algod
141b_Borre
14b_WestRe
111b_Cadiz
112b_Cadiz
112a_Cadiz
84b_Bouse
84a_Bouse
33b_Pinto
85b_Bouse
85a_Bouse
81b_Rice
96b_Dale
81a_Rice
45b_Afton
62
80a_Rice
80b_Rice
65
45a_Afton
36b_Bitter
87
76b_Dumont
43b_Amboy
62b_Crones
23a_Coyote
43a_Amboy
77b_Dumont
59b_Pinto
37b_Kelso
25b_Ibex
29a_Ibex
23b_Coyote
118b_TheWh
27b_Coyote
96a_Dale
77a_Dumont
5a_CoyoteH
44b_RedPas
14a_WestRe
59a_Pinto
75b_Dumont
62a_Crones
33a_Pinto
24b_Coyote
37a_Kelso
47a_Lenwoo
97b_Dale
27a_Coyote
36a_Bitter
97a_Dale
68b_Ibex
44a_RedPas
24a_Coyote
47b_Lenwoo
29b_Ibex
68a_Ibex
76a_Dumont
75a_Dumont
57b_Rasor
5b_CoyoteH
57a_Rasor
118a_TheWh
111a_Cadiz
25a_Ibex
Locus R35
Slatkin's s = 1
0.05

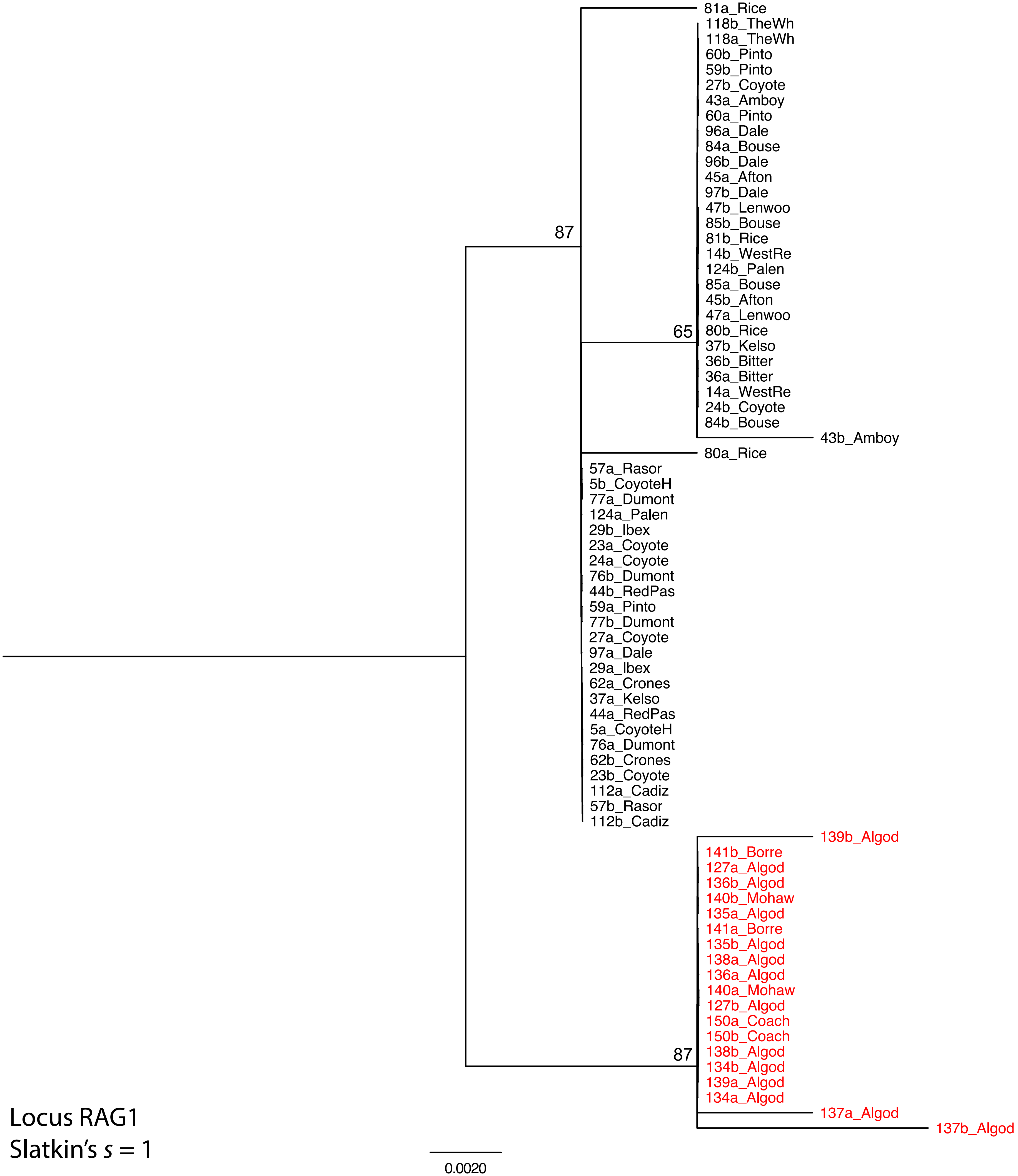
81a_Rice
118b_TheWh
118a_TheWh
60b_Pinto
59b_Pinto
27b_Coyote
43a_Amboy
60a_Pinto
96a_Dale
84a_Bouse
96b_Dale
45a_Afton
97b_Dale
47b_Lenwoo
85b_Bouse
81b_Rice
14b_WestRe
124b_Palen
85a_Bouse
45b_Afton
47a_Lenwoo
80b_Rice
37b_Kelso
36b_Bitter
36a_Bitter
14a_WestRe
24b_Coyote
84b_Bouse
43b_Amboy
80a_Rice
57a_Rasor
5b_CoyoteH
77a_Dumont
124a_Palen
29b_Ibex
23a_Coyote
24a_Coyote
76b_Dumont
44b_RedPas
59a_Pinto
77b_Dumont
27a_Coyote
97a_Dale
29a_Ibex
62a_Crones
37a_Kelso
44a_RedPas
5a_CoyoteH
76a_Dumont
62b_Crones
23b_Coyote
112a_Cadiz
57b_Rasor
112b_Cadiz
139b_Algod
141b_Borre
127a_Algod
136b_Algod
140b_Mohaw
135a_Algod
141a_Borre
135b_Algod
138a_Algod
136a_Algod
140a_Mohaw
127b_Algod
150a_Coach
150b_Coach
138b_Algod
134b_Algod
139a_Algod
134a_Algod
137a_Algod
137b_Algod
87
65
87
Locus RAG1
Slatkin's s = 1
0.0020

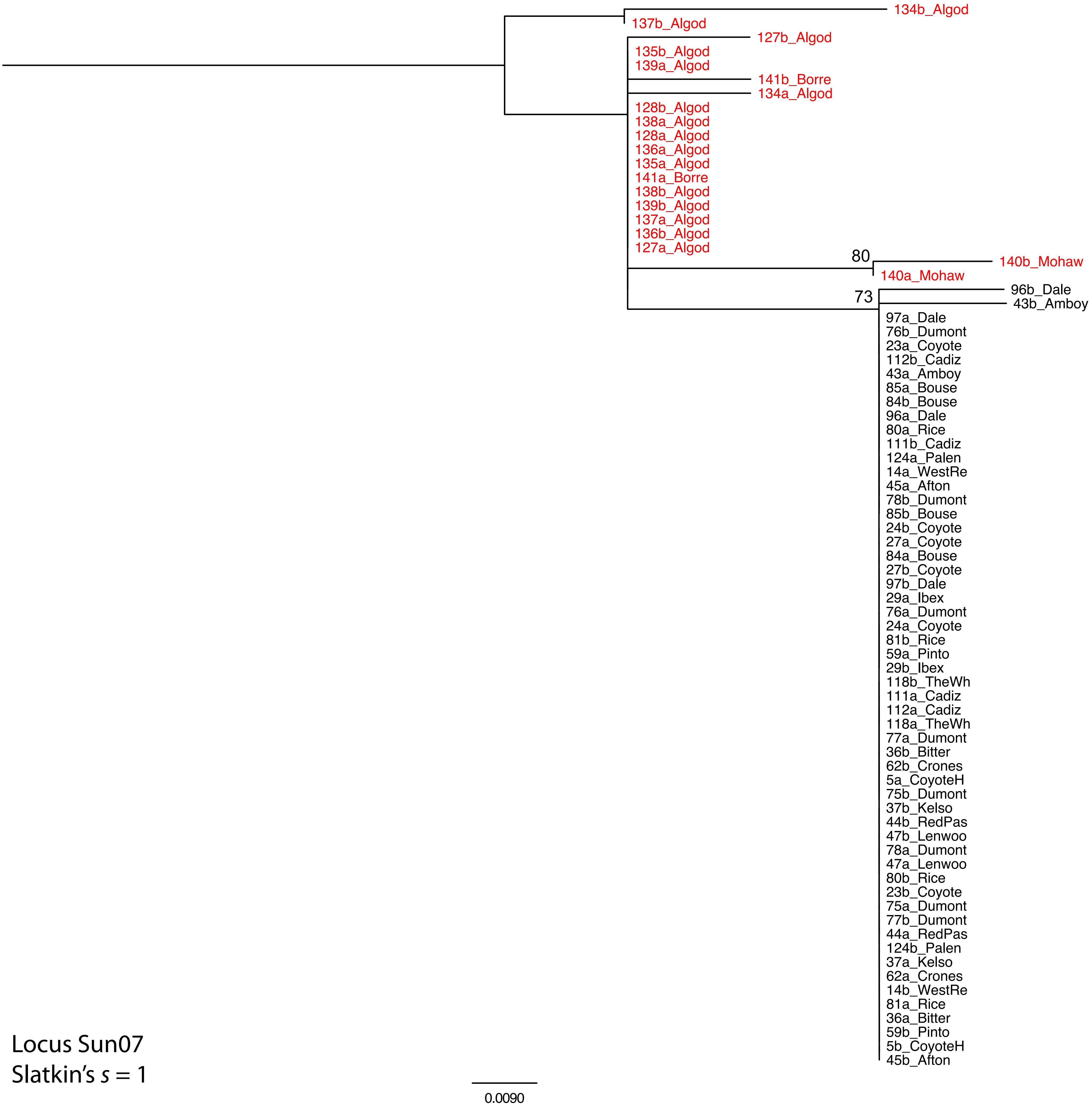

Locus Sun07

Slatkin's *s* = 1

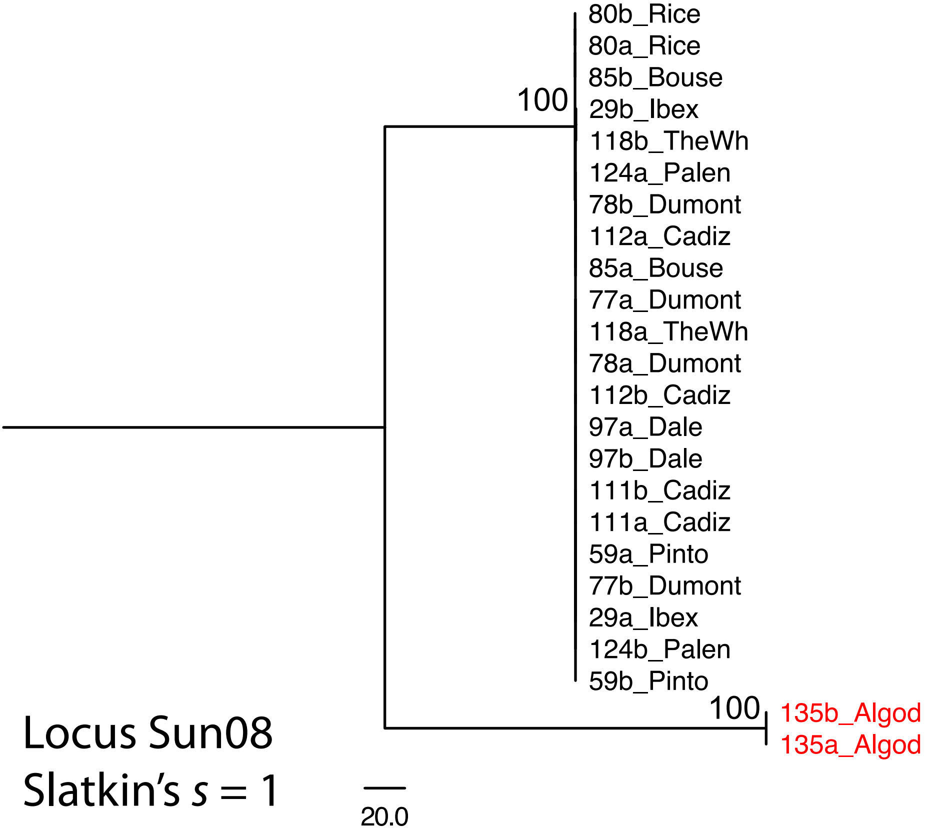

Locus Sun08

Slatkin's *s* = 1

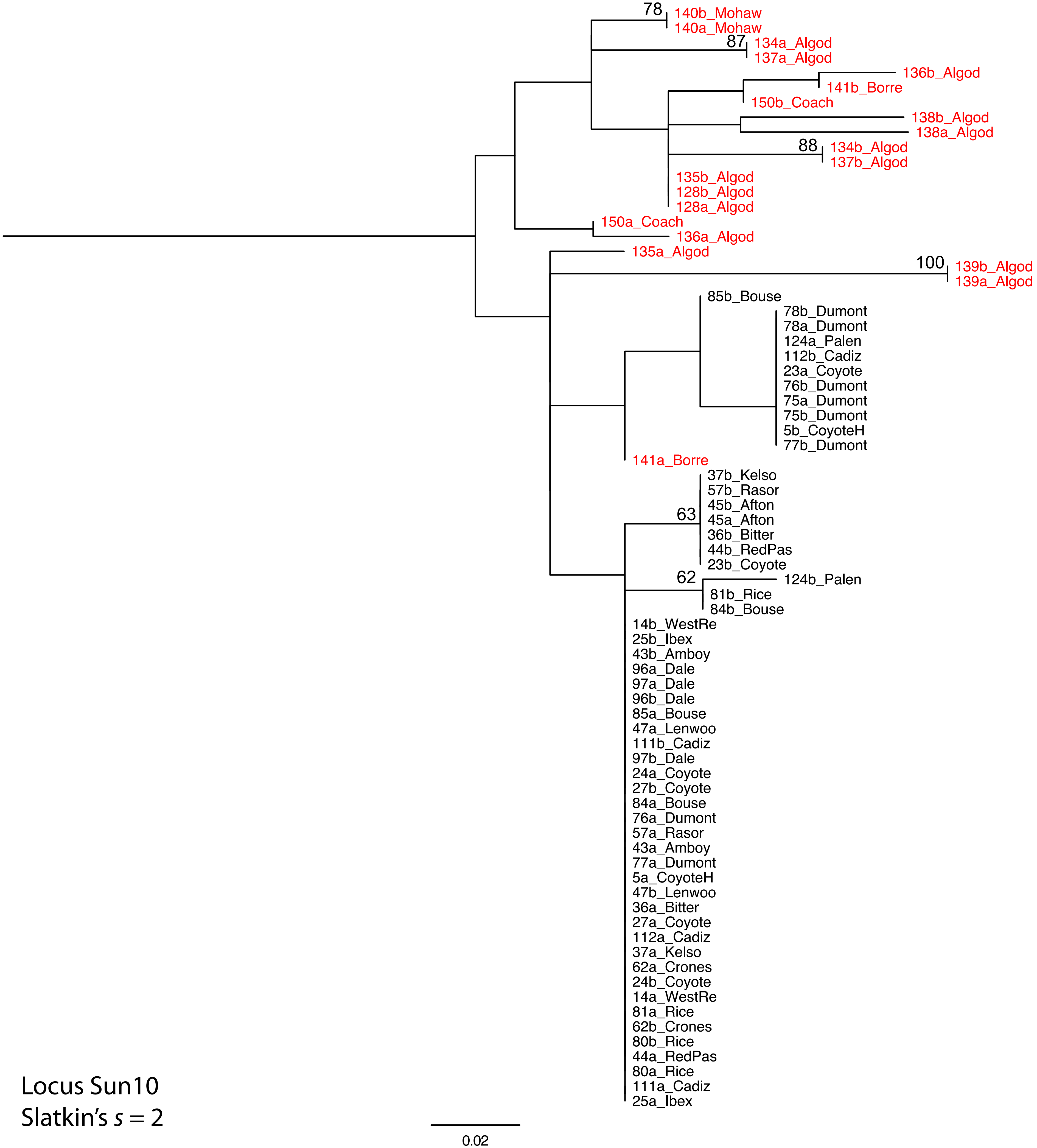

Locus Sun10
Slatkin's *s* = 2

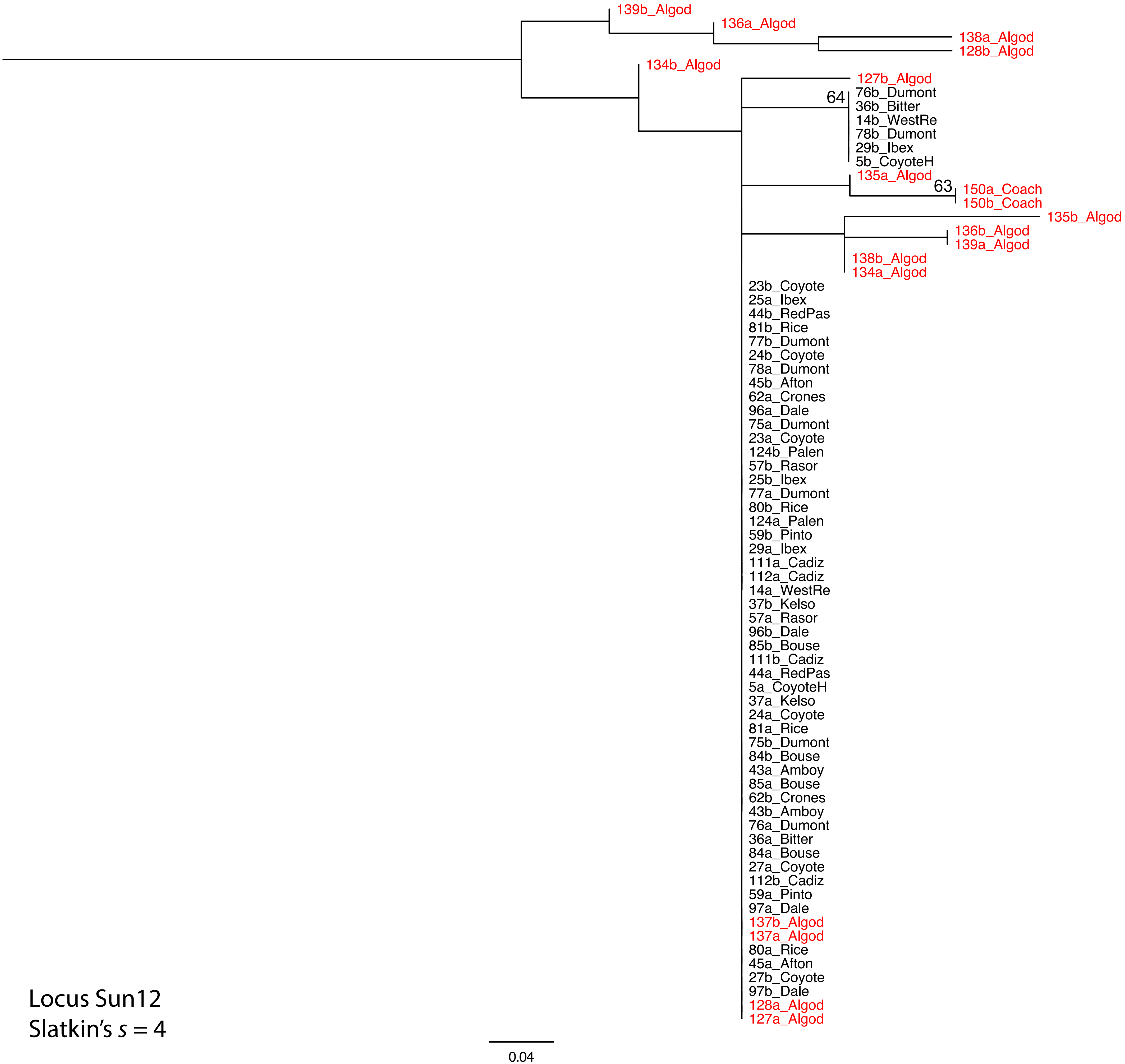

Locus Sun12
Slatkin's *s* = 4

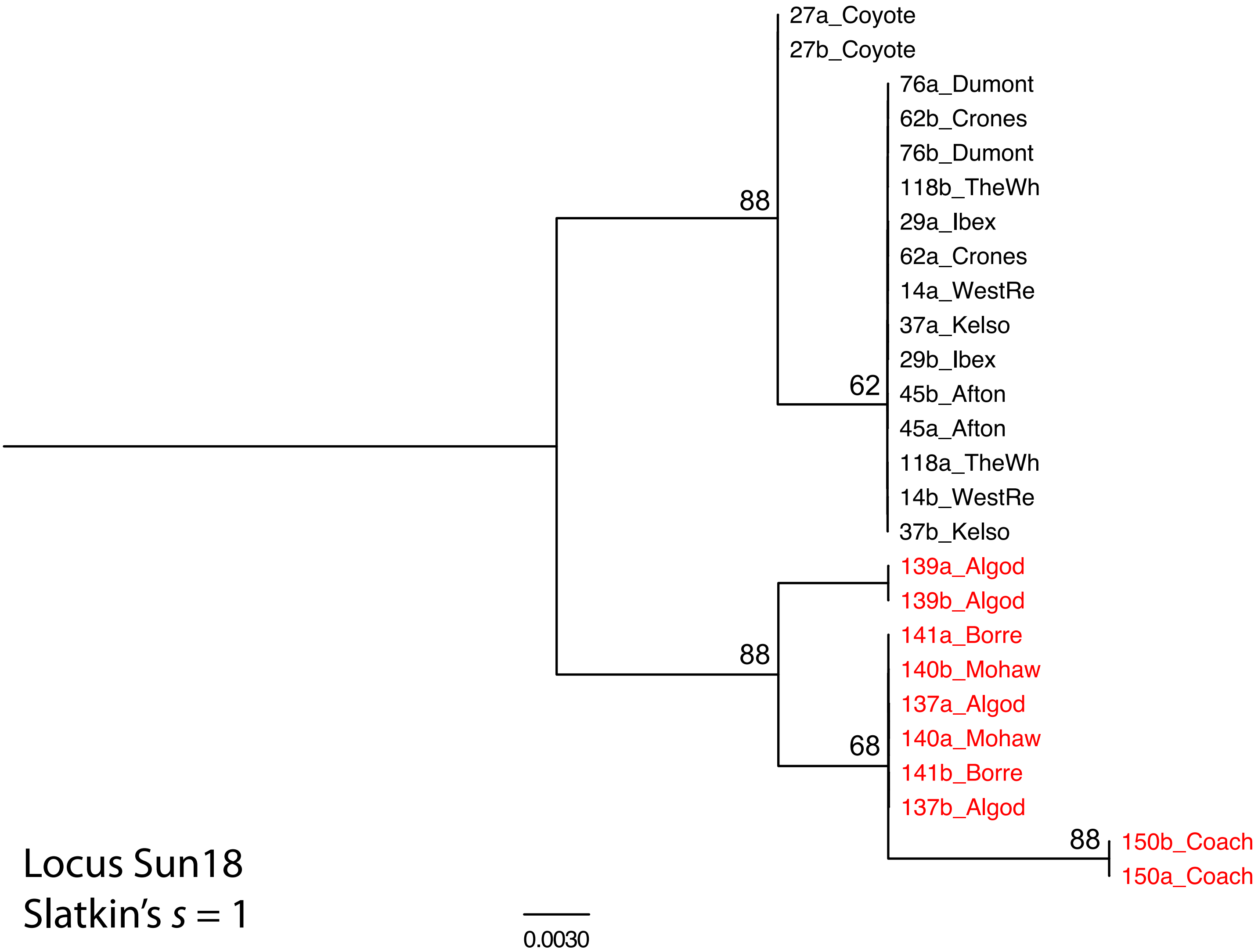
27a_Coyote
27b_Coyote
76a_Dumont
62b_Crones
76b_Dumont
118b_TheWh
29a_Ibex
62a_Crones
14a_WestRe
37a_Kelso
29b_Ibex
45b_Afton
45a_Afton
118a_TheWh
14b_WestRe
37b_Kelso
139a_Algod
139b_Algod
141a_Borre
140b_Mohaw
137a_Algod
140a_Mohaw
141b_Borre
137b_Algod
150b_Coach
150a_Coach
88
62
88
68
88
0.0030
Locus Sun18
Slatkin's *s* = 1

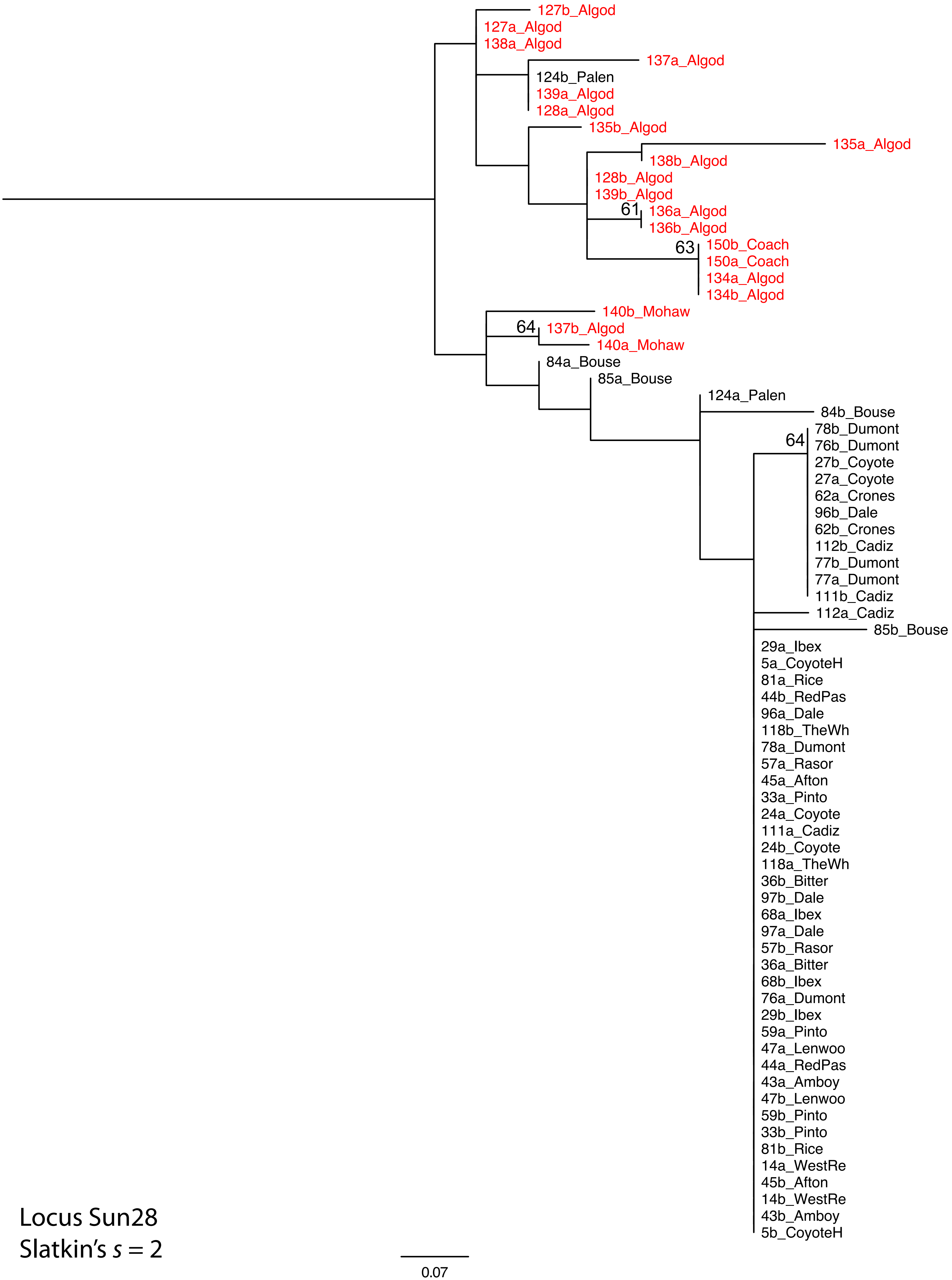
127b_Algod
127a_Algod
138a_Algod
137a_Algod
124b_Palen
139a_Algod
128a_Algod
135b_Algod
135a_Algod
138b_Algod
128b_Algod
139b_Algod
61
136a_Algod
136b_Algod
63
150b_Coach
150a_Coach
134a_Algod
134b_Algod
140b_Mohaw
64
137b_Algod
140a_Mohaw
84a_Bouse
85a_Bouse
124a_Palen
84b_Bouse
64
78b_Dumont
76b_Dumont
27b_Coyote
27a_Coyote
62a_Crones
96b_Dale
62b_Crones
112b_Cadiz
77b_Dumont
77a_Dumont
111b_Cadiz
112a_Cadiz
85b_Bouse
29a_Ibex
5a_CoyoteH
81a_Rice
44b_RedPas
96a_Dale
118b_TheWh
78a_Dumont
57a_Rasor
45a_Afton
33a_Pinto
24a_Coyote
111a_Cadiz
24b_Coyote
118a_TheWh
36b_Bitter
97b_Dale
68a_Ibex
97a_Dale
57b_Rasor
36a_Bitter
68b_Ibex
76a_Dumont
29b_Ibex
59a_Pinto
47a_Lenwoo
44a_RedPas
43a_Amboy
47b_Lenwoo
59b_Pinto
33b_Pinto
81b_Rice
14a_WestRe
45b_Afton
14b_WestRe
43b_Amboy
5b_CoyoteH
Locus Sun28
Slatkin's s = 2
0.07

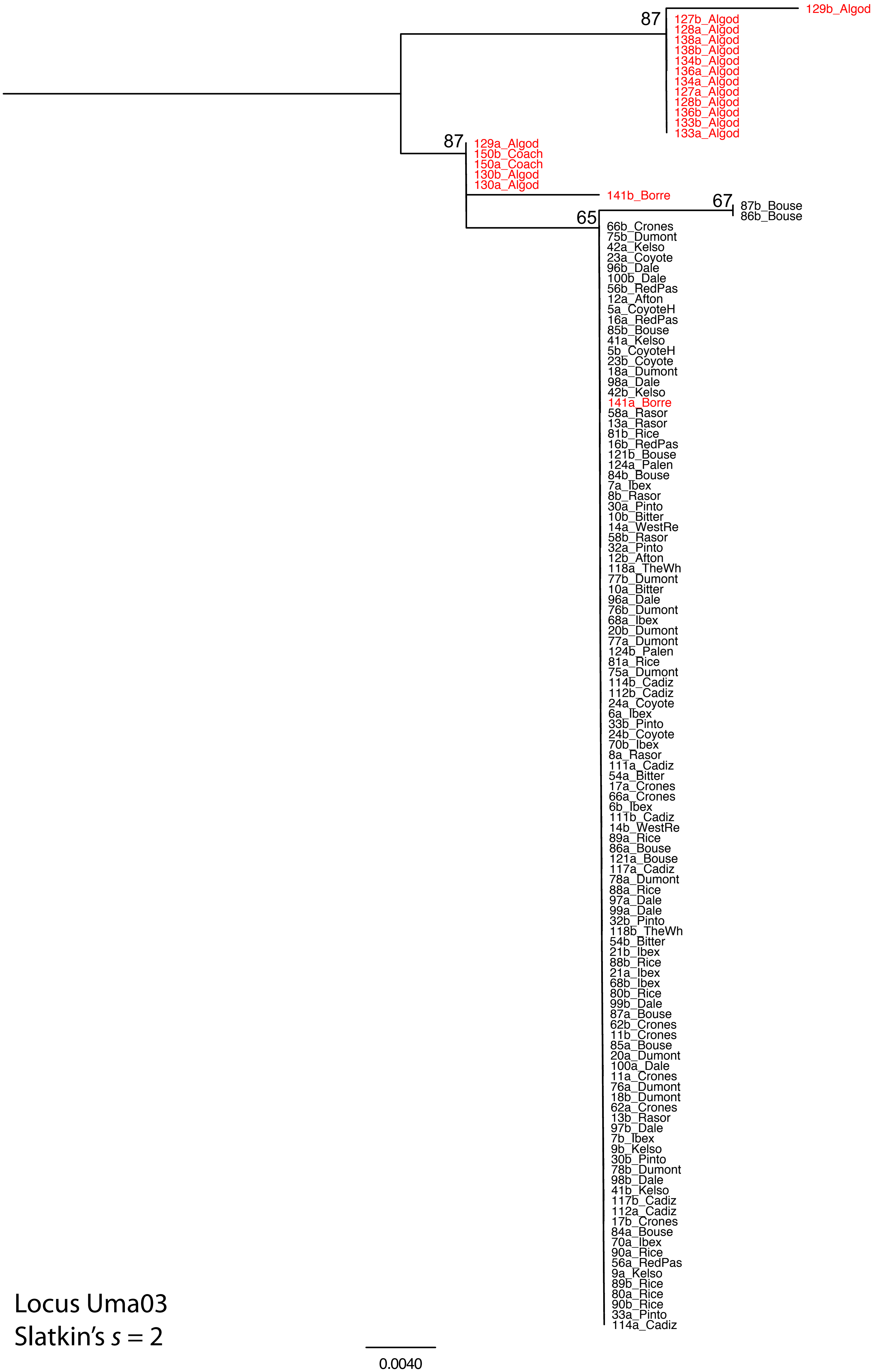

129b_Algod
87
127b_Algod
128a_Algod
138a_Algod
138b_Algod
134b_Algod
136a_Algod
134a_Algod
127a_Algod
128b_Algod
136b_Algod
133b_Algod
133a_Algod
87
129a_Algod
150b_Coach
150a_Coach
130b_Algod
130a_Algod
141b_Borre
67
87b_Bouse
86b_Bouse
65
66b_Crones
75b_Dumont
42a_Kelso
23a_Coyote
96b_Dale
100b_Dale
56b_RedPas
12a_Afton
5a_CoyoteH
16a_RedPas
85b_Bouse
41a_Kelso
5b_CoyoteH
23b_Coyote
18a_Dumont
98a_Dale
42b_Kelso
141a_Borre
58a_Rasor
13a_Rasor
81b_Rice
16b_RedPas
121b_Bouse
124a_Palen
84b_Bouse
7a_Ibex
8b_Rasor
30a_Pinto
10b_Bitter
14a_WestRe
58b_Rasor
32a_Pinto
12b_Afton
118a_TheWh
77b_Dumont
10a_Bitter
96a_Dale
76b_Dumont
68a_Ibex
20b_Dumont
77a_Dumont
124b_Palen
81a_Rice
75a_Dumont
114b_Cadiz
112b_Cadiz
24a_Coyote
6a_Ibex
33b_Pinto
24b_Coyote
70b_Ibex
8a_Rasor
111a_Cadiz
54a_Bitter
17a_Crones
66a_Crones
6b_Ibex
111b_Cadiz
14b_WestRe
89a_Rice
86a_Bouse
121a_Bouse
117a_Cadiz
78a_Dumont
88a_Rice
97a_Dale
99a_Dale
32b_Pinto
118b_TheWh
54b_Bitter
21b_Ibex
88b_Rice
21a_Ibex
68b_Ibex
80b_Rice
99b_Dale
87a_Bouse
62b_Crones
11b_Crones
85a_Bouse
20a_Dumont
100a_Dale
11a_Crones
76a_Dumont
18b_Dumont
62a_Crones
13b_Rasor
97b_Dale
7b_Ibex
9b_Kelso
30b_Pinto
78b_Dumont
98b_Dale
41b_Kelso
117b_Cadiz
112a_Cadiz
17b_Crones
84a_Bouse
70a_Ibex
90a_Rice
56a_RedPas
9a_Kelso
89b_Rice
80a_Rice
90b_Rice
33a_Pinto
114a_Cadiz
Locus Uma03
Slatkin's s = 2
0.0040

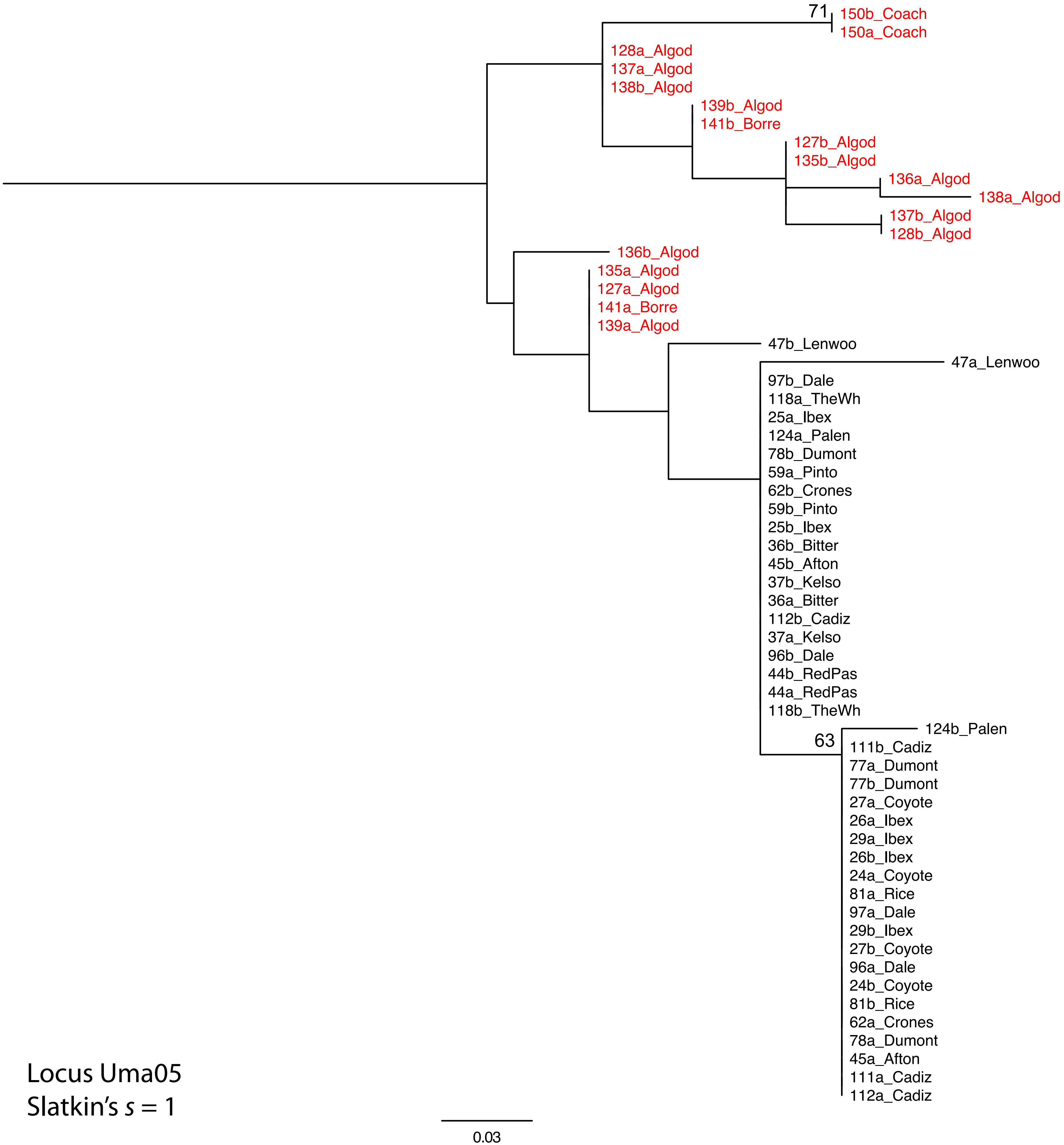
71
150b_Coach
150a_Coach
128a_Algod
137a_Algod
138b_Algod
139b_Algod
141b_Borre
127b_Algod
135b_Algod
136a_Algod
138a_Algod
137b_Algod
128b_Algod
136b_Algod
135a_Algod
127a_Algod
141a_Borre
139a_Algod
47b_Lenwoo
47a_Lenwoo
97b_Dale
118a_TheWh
25a_Ibex
124a_Palen
78b_Dumont
59a_Pinto
62b_Crones
59b_Pinto
25b_Ibex
36b_Bitter
45b_Afton
37b_Kelso
36a_Bitter
112b_Cadiz
37a_Kelso
96b_Dale
44b_RedPas
44a_RedPas
118b_TheWh
124b_Palen
63
111b_Cadiz
77a_Dumont
77b_Dumont
27a_Coyote
26a_Ibex
29a_Ibex
26b_Ibex
24a_Coyote
81a_Rice
97a_Dale
29b_Ibex
27b_Coyote
96a_Dale
24b_Coyote
81b_Rice
62a_Crones
78a_Dumont
45a_Afton
111a_Cadiz
112a_Cadiz
Locus Uma05
Slatkin's s = 1
0.03

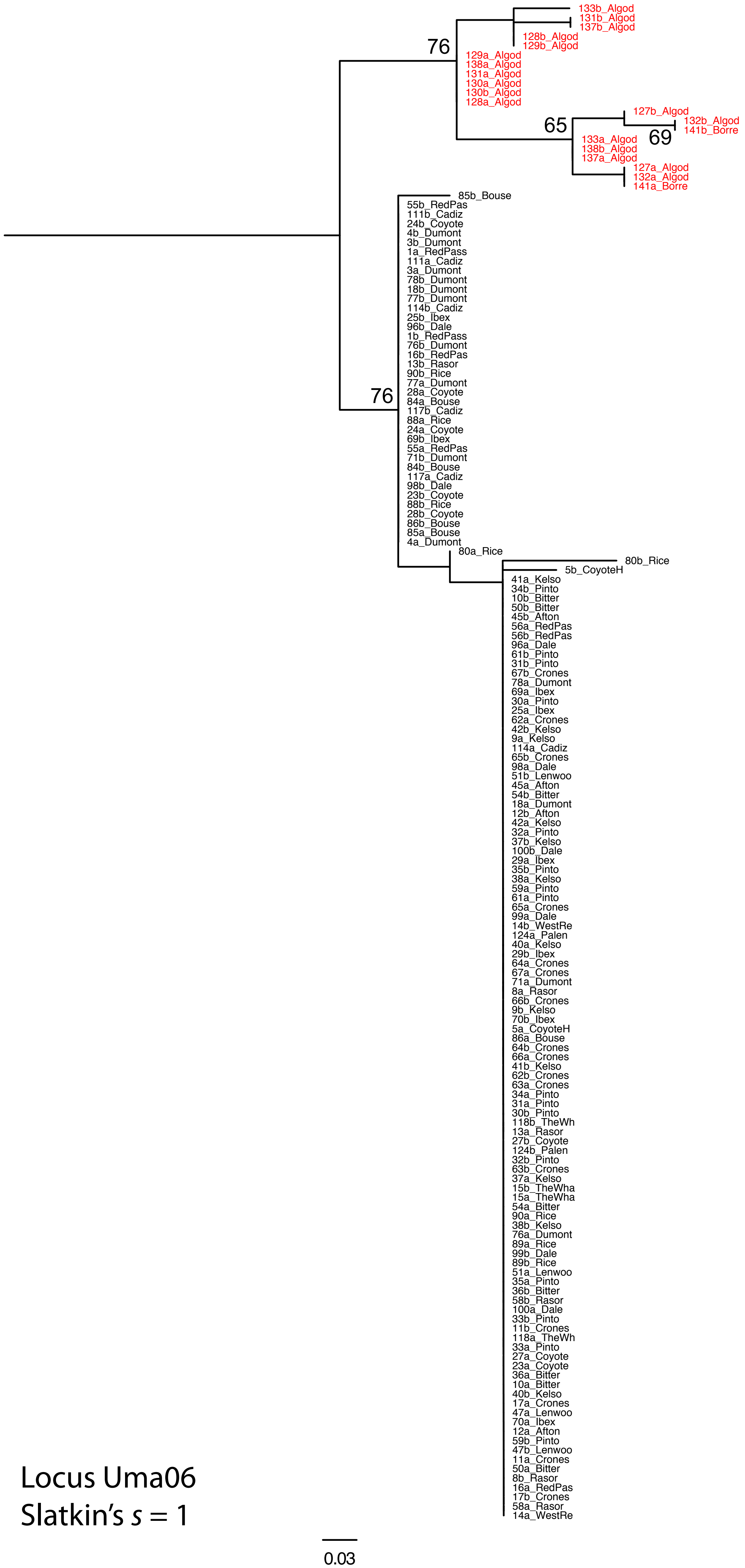
133b_Algod
131b_Algod
137b_Algod
128b_Algod
129b_Algod
76
129a_Algod
138a_Algod
131a_Algod
130a_Algod
130b_Algod
128a_Algod
127b_Algod
65
132b_Algod
141b_Borre
133a_Algod
138b_Algod
137a_Algod
69
127a_Algod
132a_Algod
141a_Borre
85b_Bouse
55b_RedPas
111b_Cadiz
24b_Coyote
4b_Dumont
3b_Dumont
1a_RedPass
111a_Cadiz
3a_Dumont
78b_Dumont
18b_Dumont
77b_Dumont
114b_Cadiz
25b_Ibex
96b_Dale
1b_RedPass
76b_Dumont
16b_RedPas
13b_Rasor
90b_Rice
77a_Dumont
76
28a_Coyote
84a_Bouse
117b_Cadiz
88a_Rice
24a_Coyote
69b_Ibex
55a_RedPas
71b_Dumont
84b_Bouse
117a_Cadiz
98b_Dale
23b_Coyote
88b_Rice
28b_Coyote
86b_Bouse
85a_Bouse
4a_Dumont
80a_Rice
80b_Rice
5b_CoyoteH
41a_Kelso
34b_Pinto
10b_Bitter
50b_Bitter
45b_Afton
56a_RedPas
56b_RedPas
96a_Dale
61b_Pinto
31b_Pinto
67b_Crones
78a_Dumont
69a_Ibex
30a_Pinto
25a_Ibex
62a_Crones
42b_Kelso
9a_Kelso
114a_Cadiz
65b_Crones
98a_Dale
51b_Lenwoo
45a_Afton
54b_Bitter
18a_Dumont
12b_Afton
42a_Kelso
32a_Pinto
37b_Kelso
100b_Dale
29a_Ibex
35b_Pinto
38a_Kelso
59a_Pinto
61a_Pinto
65a_Crones
99a_Dale
14b_WestRe
124a_Palen
40a_Kelso
29b_Ibex
64a_Crones
67a_Crones
71a_Dumont
8a_Rasor
66b_Crones
9b_Kelso
70b_Ibex
5a_CoyoteH
86a_Bouse
64b_Crones
66a_Crones
41b_Kelso
62b_Crones
63a_Crones
34a_Pinto
31a_Pinto
30b_Pinto
118b_TheWh
13a_Rasor
27b_Coyote
124b_Palen
32b_Pinto
63b_Crones
37a_Kelso
15b_TheWha
15a_TheWha
54a_Bitter
90a_Rice
38b_Kelso
76a_Dumont
89a_Rice
99b_Dale
89b_Rice
51a_Lenwoo
35a_Pinto
36b_Bitter
58b_Rasor
100a_Dale
33b_Pinto
11b_Crones
118a_TheWh
33a_Pinto
27a_Coyote
23a_Coyote
36a_Bitter
10a_Bitter
40b_Kelso
17a_Crones
47a_Lenwoo
70a_Ibex
12a_Afton
59b_Pinto
47b_Lenwoo
11a_Crones
50a_Bitter
8b_Rasor
16a_RedPas
17b_Crones
58a_Rasor
14a_WestRe
Locus Uma06
Slatkin's *s* = 1
0.03

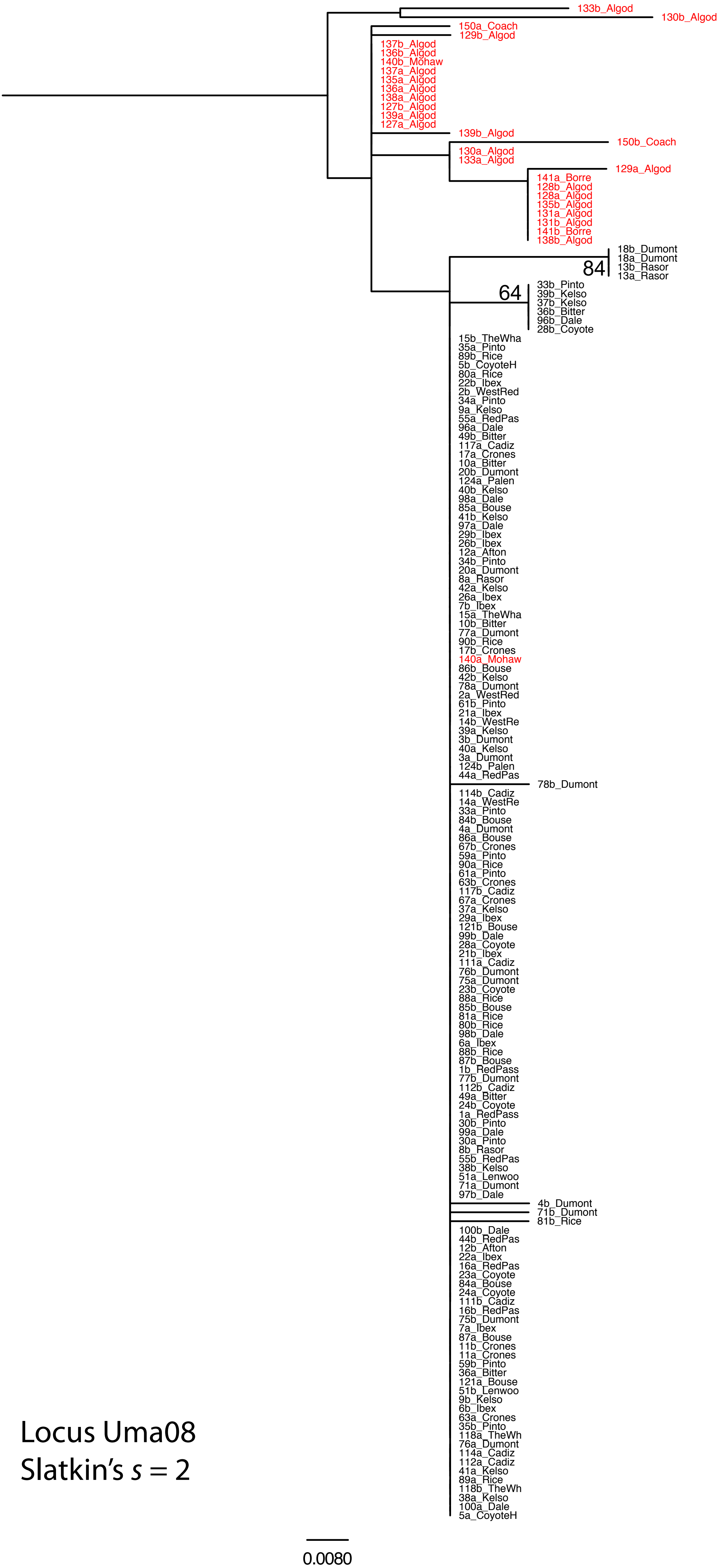

133b_Algod
130b_Algod
150a_Coach
129b_Algod
137b_Algod
136b_Algod
140b_Mohaw
137a_Algod
135a_Algod
136a_Algod
138a_Algod
127b_Algod
139a_Algod
127a_Algod
139b_Algod
150b_Coach
130a_Algod
133a_Algod
129a_Algod
141a_Borre
128b_Algod
128a_Algod
135b_Algod
131a_Algod
131b_Algod
141b_Borre
138b_Algod
18b_Dumont
18a_Dumont
13b_Rasor
13a_Rasor
84
64
33b_Pinto
39b_Kelso
37b_Kelso
36b_Bitter
96b_Dale
28b_Coyote
15b_TheWha
35a_Pinto
89b_Rice
5b_CoyoteH
80a_Rice
22b_Ibex
2b_WestRed
34a_Pinto
9a_Kelso
55a_RedPas
96a_Dale
49b_Bitter
117a_Cadiz
17a_Crones
10a_Bitter
20b_Dumont
124a_Palen
40b_Kelso
98a_Dale
85a_Bouse
41b_Kelso
97a_Dale
29b_Ibex
26b_Ibex
12a_Afton
34b_Pinto
20a_Dumont
8a_Rasor
42a_Kelso
26a_Ibex
7b_Ibex
15a_TheWha
10b_Bitter
77a_Dumont
90b_Rice
17b_Crones
140a_Mohaw
86b_Bouse
42b_Kelso
78a_Dumont
2a_WestRed
61b_Pinto
21a_Ibex
14b_WestRe
39a_Kelso
3b_Dumont
40a_Kelso
3a_Dumont
124b_Palen
44a_RedPas
78b_Dumont
114b_Cadiz
14a_WestRe
33a_Pinto
84b_Bouse
4a_Dumont
86a_Bouse
67b_Crones
59a_Pinto
90a_Rice
61a_Pinto
63b_Crones
117b_Cadiz
67a_Crones
37a_Kelso
29a_Ibex
121b_Bouse
99b_Dale
28a_Coyote
21b_Ibex
111a_Cadiz
76b_Dumont
75a_Dumont
23b_Coyote
88a_Rice
85b_Bouse
81a_Rice
80b_Rice
98b_Dale
6a_Ibex
88b_Rice
87b_Bouse
1b_RedPass
77b_Dumont
112b_Cadiz
49a_Bitter
24b_Coyote
1a_RedPass
30b_Pinto
99a_Dale
30a_Pinto
8b_Rasor
55b_RedPas
38b_Kelso
51a_Lenwoo
71a_Dumont
97b_Dale
4b_Dumont
71b_Dumont
81b_Rice
100b_Dale
44b_RedPas
12b_Afton
22a_Ibex
16a_RedPas
23a_Coyote
84a_Bouse
24a_Coyote
111b_Cadiz
16b_RedPas
75b_Dumont
7a_Ibex
87a_Bouse
11b_Crones
11a_Crones
59b_Pinto
36a_Bitter
121a_Bouse
51b_Lenwoo
9b_Kelso
6b_Ibex
63a_Crones
35b_Pinto
118a_TheWh
76a_Dumont
114a_Cadiz
112a_Cadiz
41a_Kelso
89a_Rice
118b_TheWh
38a_Kelso
100a_Dale
5a_CoyoteH
Locus Uma08
Slatkin's s = 2
0.0080
