# Supplementary material for: Speciation, population structure, and demographic history of the Mojave Fringe-toed Lizard (Uma scoparia), a species of conservation concern
Source: Ecol Evol. 2014 May 24;4(12):2546–62. doi: 10.1002/ece3.1111 (PMC4203297; doi:10.1002/ece3.1111)
Supplement: Supplementary file 8 — File S2. Bayesian 50% majority rule consensus trees, estimated with MrBayes v3.2.2 (Ronquist et al. 2012), for all fourteen loci with posterior probabilities mapped onto nodes. U. scoparia are shown in black and the U. notata complex in red. Slatkin's s-values are shown for each locus. [file ece30004-2546-sd8.pdf]

Supplemental File 2. Bayesian 50% majority rule consensus trees, estimated with MrBayes v3.2.2 (Ronquist *et al*. 2012), for all fourteen loci with posterior probabilities mapped onto nodes. *U. scoparia* are shown in black and the *U. notata* complex in red. Slatkin's *s* values are shown for each locus.

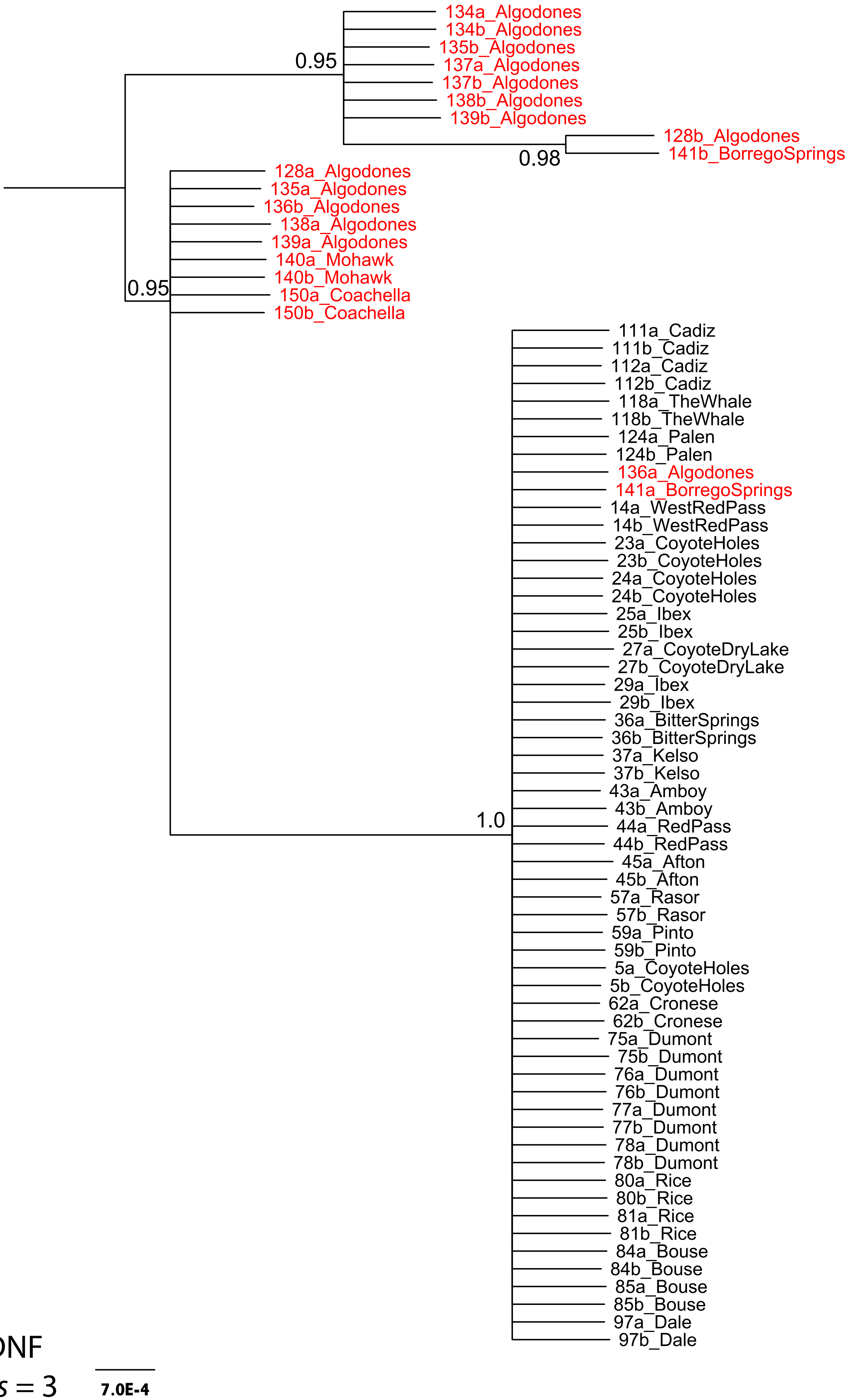

Locus BDNF

Slatkin's *s* = 3

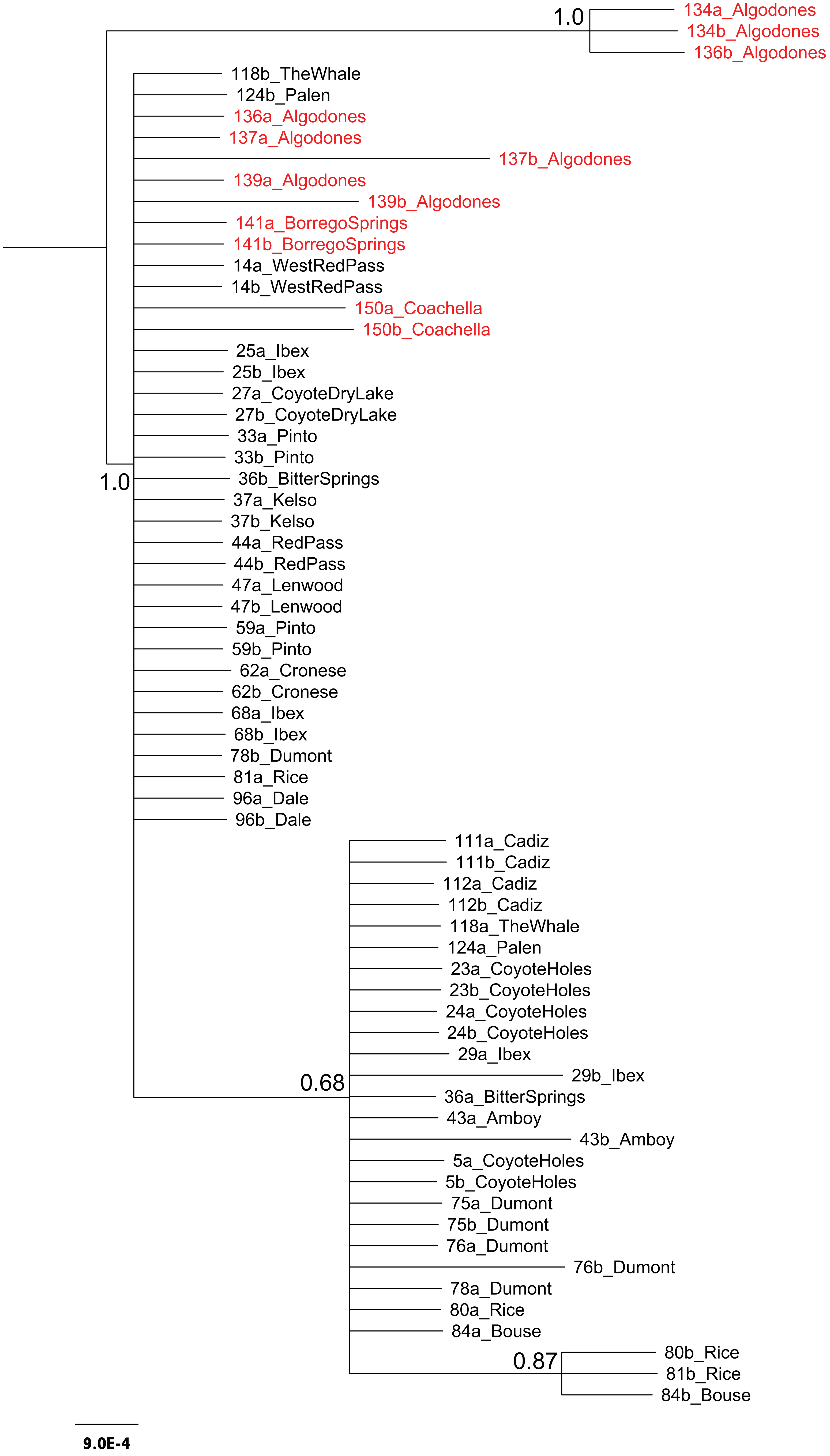

Locus PNN

Slatkin's $s = 10$

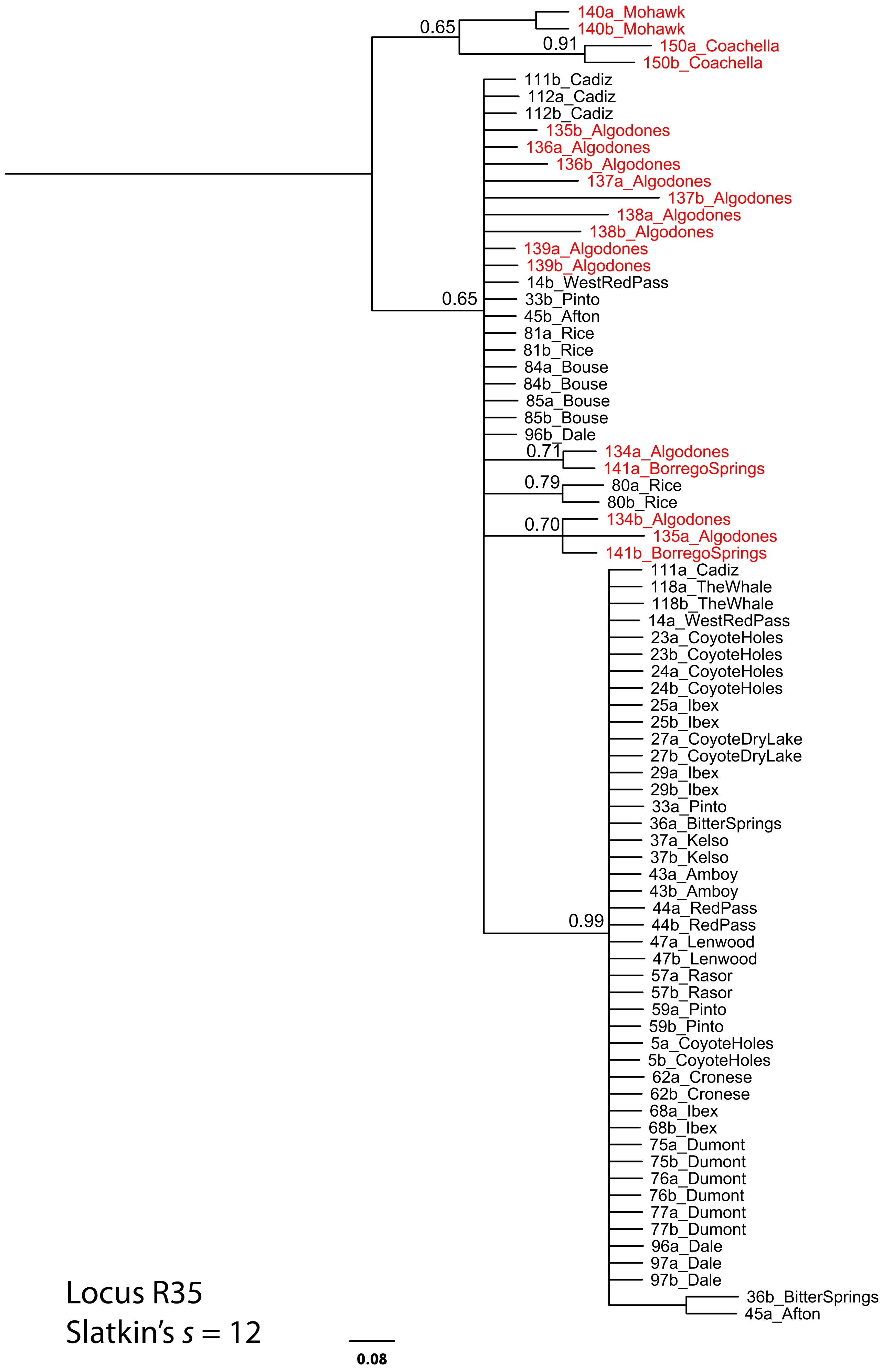

0.65
140a_Mohawk
140b_Mohawk
0.91
150a_Coachella
150b_Coachella
111b_Cadiz
112a_Cadiz
112b_Cadiz
135b_Algodones
136a_Algodones
136b_Algodones
137a_Algodones
137b_Algodones
138a_Algodones
138b_Algodones
139a_Algodones
139b_Algodones
14b_WestRedPass
0.65
33b_Pinto
45b_Afton
81a_Rice
81b_Rice
84a_Bouse
84b_Bouse
85a_Bouse
85b_Bouse
96b_Dale
0.71
134a_Algodones
141a_BorregoSprings
0.79
80a_Rice
80b_Rice
0.70
134b_Algodones
135a_Algodones
141b_BorregoSprings
111a_Cadiz
118a_TheWhale
118b_TheWhale
14a_WestRedPass
23a_CoyoteHoles
23b_CoyoteHoles
24a_CoyoteHoles
24b_CoyoteHoles
25a_Ibex
25b_Ibex
27a_CoyoteDryLake
27b_CoyoteDryLake
29a_Ibex
29b_Ibex
33a_Pinto
36a_BitterSprings
37a_Kelso
37b_Kelso
43a_Amboy
43b_Amboy
44a_RedPass
0.99
44b_RedPass
47a_Lenwood
47b_Lenwood
57a_Rasor
57b_Rasor
59a_Pinto
59b_Pinto
5a_CoyoteHoles
5b_CoyoteHoles
62a_Cronese
62b_Cronese
68a_Ibex
68b_Ibex
75a_Dumont
75b_Dumont
76a_Dumont
76b_Dumont
77a_Dumont
77b_Dumont
96a_Dale
97a_Dale
97b_Dale
36b_BitterSprings
45a_Afton
Locus R35
Slatkin's s = 12
0.08

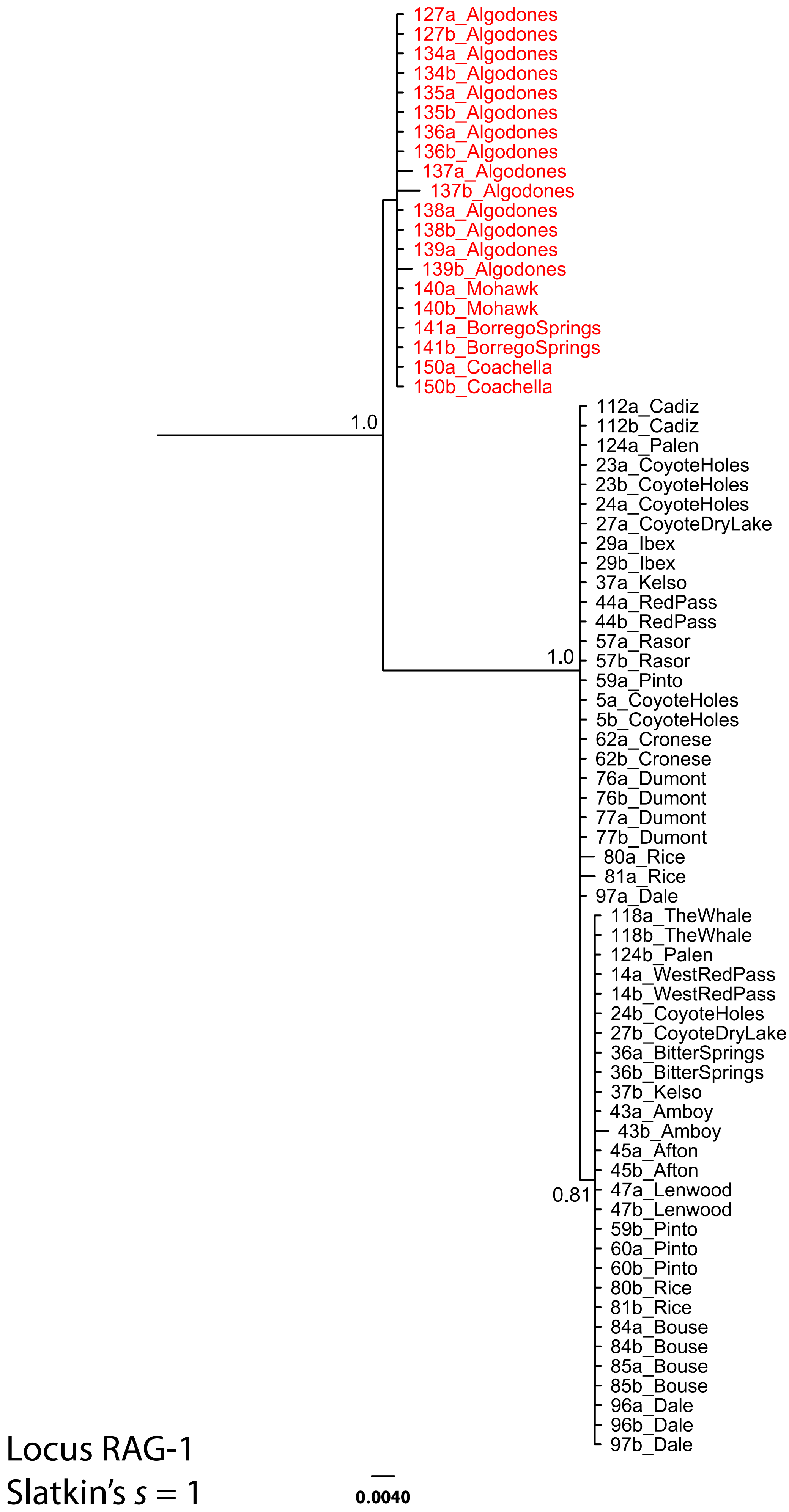
127a_Algodones
127b_Algodones
134a_Algodones
134b_Algodones
135a_Algodones
135b_Algodones
136a_Algodones
136b_Algodones
137a_Algodones
137b_Algodones
138a_Algodones
138b_Algodones
139a_Algodones
139b_Algodones
140a_Mohawk
140b_Mohawk
141a_BorregoSprings
141b_BorregoSprings
150a_Coachella
150b_Coachella
1.0
112a_Cadiz
112b_Cadiz
124a_Palen
23a_CoyoteHoles
23b_CoyoteHoles
24a_CoyoteHoles
27a_CoyoteDryLake
29a_Ibex
29b_Ibex
37a_Kelso
44a_RedPass
44b_RedPass
57a_Rasor
57b_Rasor
1.0
59a_Pinto
5a_CoyoteHoles
5b_CoyoteHoles
62a_Cronese
62b_Cronese
76a_Dumont
76b_Dumont
77a_Dumont
77b_Dumont
80a_Rice
81a_Rice
97a_Dale
118a_TheWhale
118b_TheWhale
124b_Palen
14a_WestRedPass
14b_WestRedPass
24b_CoyoteHoles
27b_CoyoteDryLake
36a_BitterSprings
36b_BitterSprings
37b_Kelso
43a_Amboy
43b_Amboy
45a_Afton
45b_Afton
0.81
47a_Lenwood
47b_Lenwood
59b_Pinto
60a_Pinto
60b_Pinto
80b_Rice
81b_Rice
84a_Bouse
84b_Bouse
85a_Bouse
85b_Bouse
96a_Dale
96b_Dale
97b_Dale
Locus RAG-1
Slatkin's s = 1
0.0040

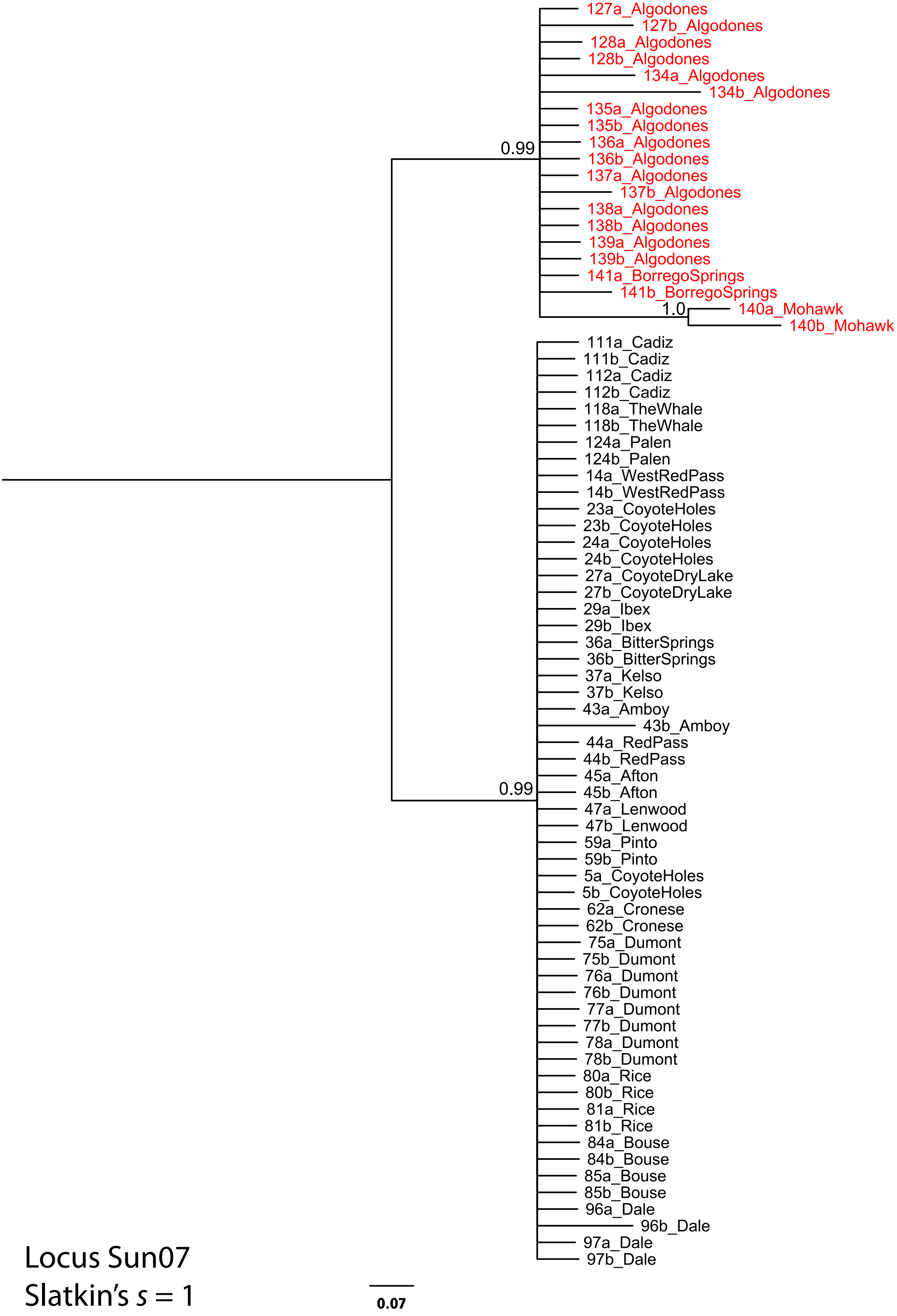

Locus Sun07
Slatkin's *s* = 1

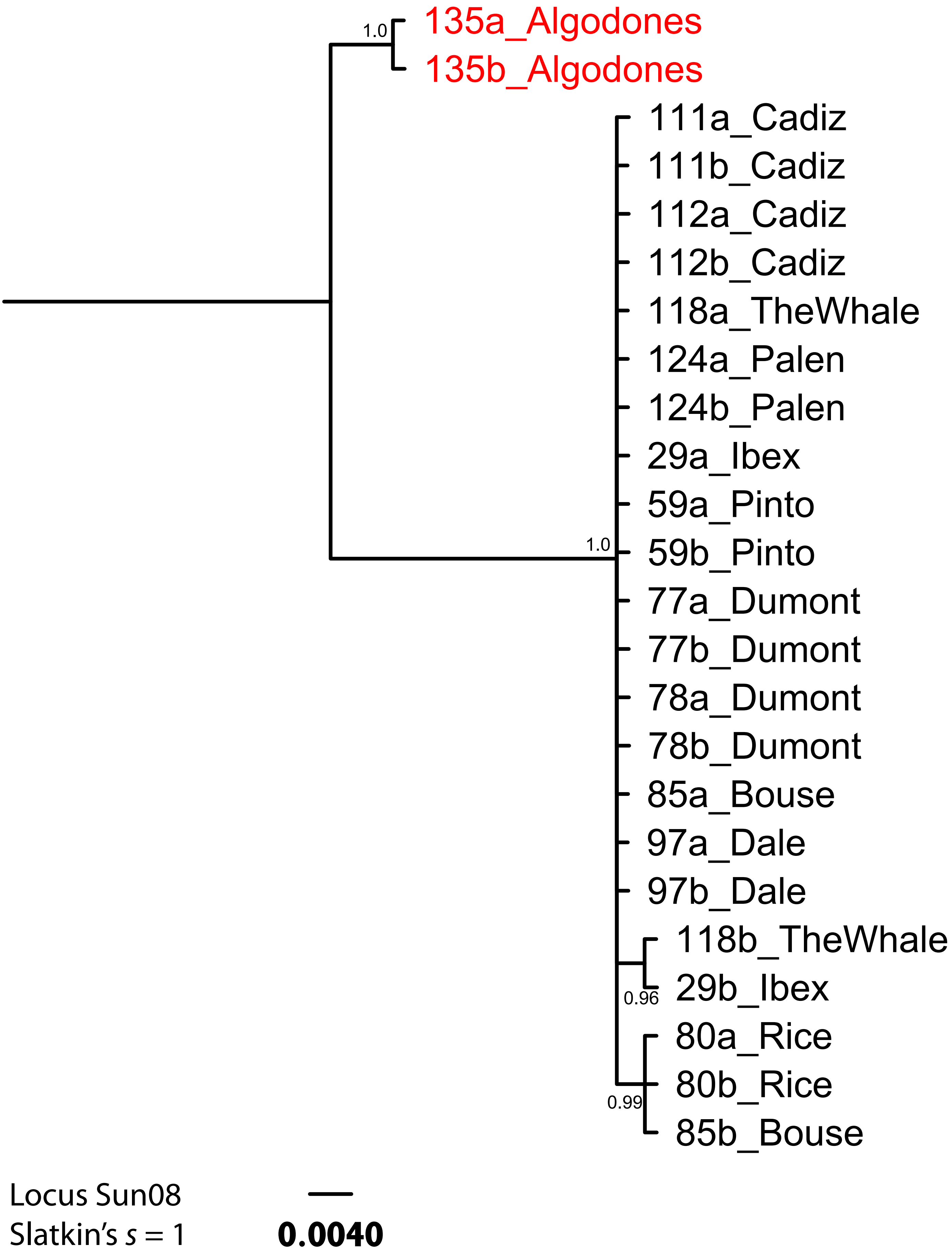
1.0
135a_Algodones
135b_Algodones
111a_Cadiz
111b_Cadiz
112a_Cadiz
112b_Cadiz
118a_TheWhale
124a_Palen
124b_Palen
29a_Ibex
59a_Pinto
1.0
59b_Pinto
77a_Dumont
77b_Dumont
78a_Dumont
78b_Dumont
85a_Bouse
97a_Dale
97b_Dale
118b_TheWhale
29b_Ibex
0.96
80a_Rice
80b_Rice
0.99
85b_Bouse
Locus Sun08
Slatkin's s = 1
0.0040

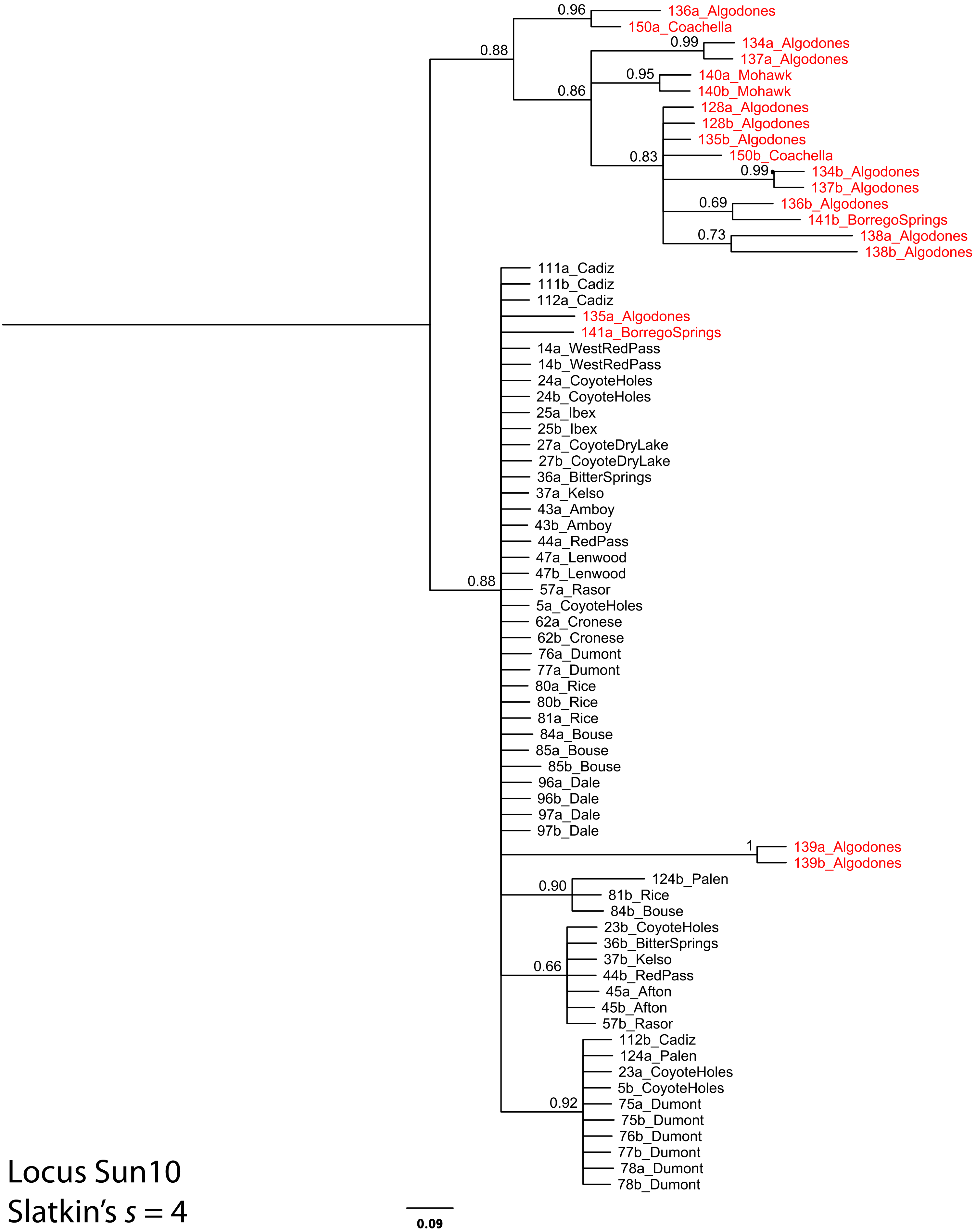

Locus Sun10

Slatkin's *s* = 4

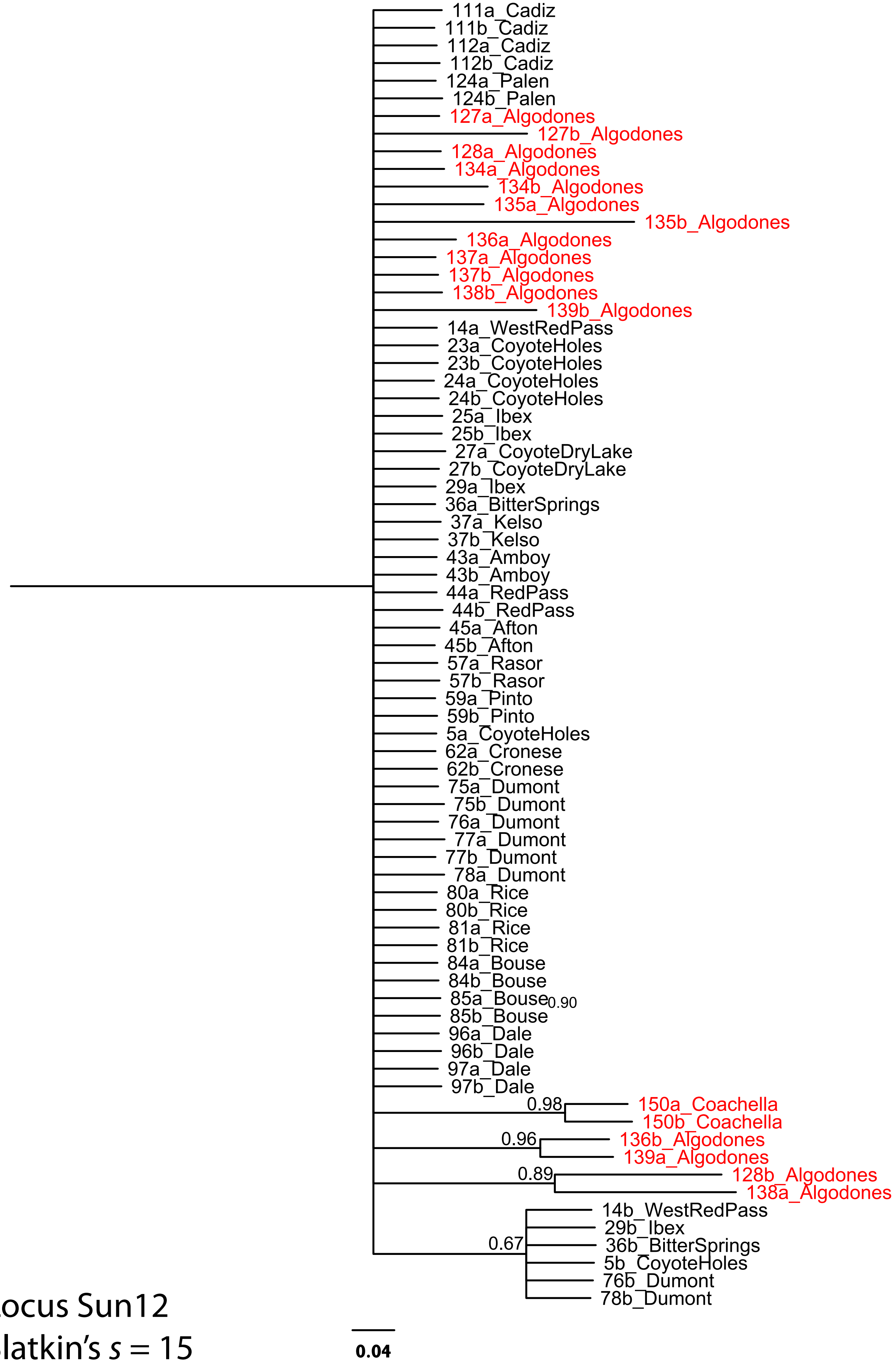

Locus Sun12

Slatkin's *s* = 15

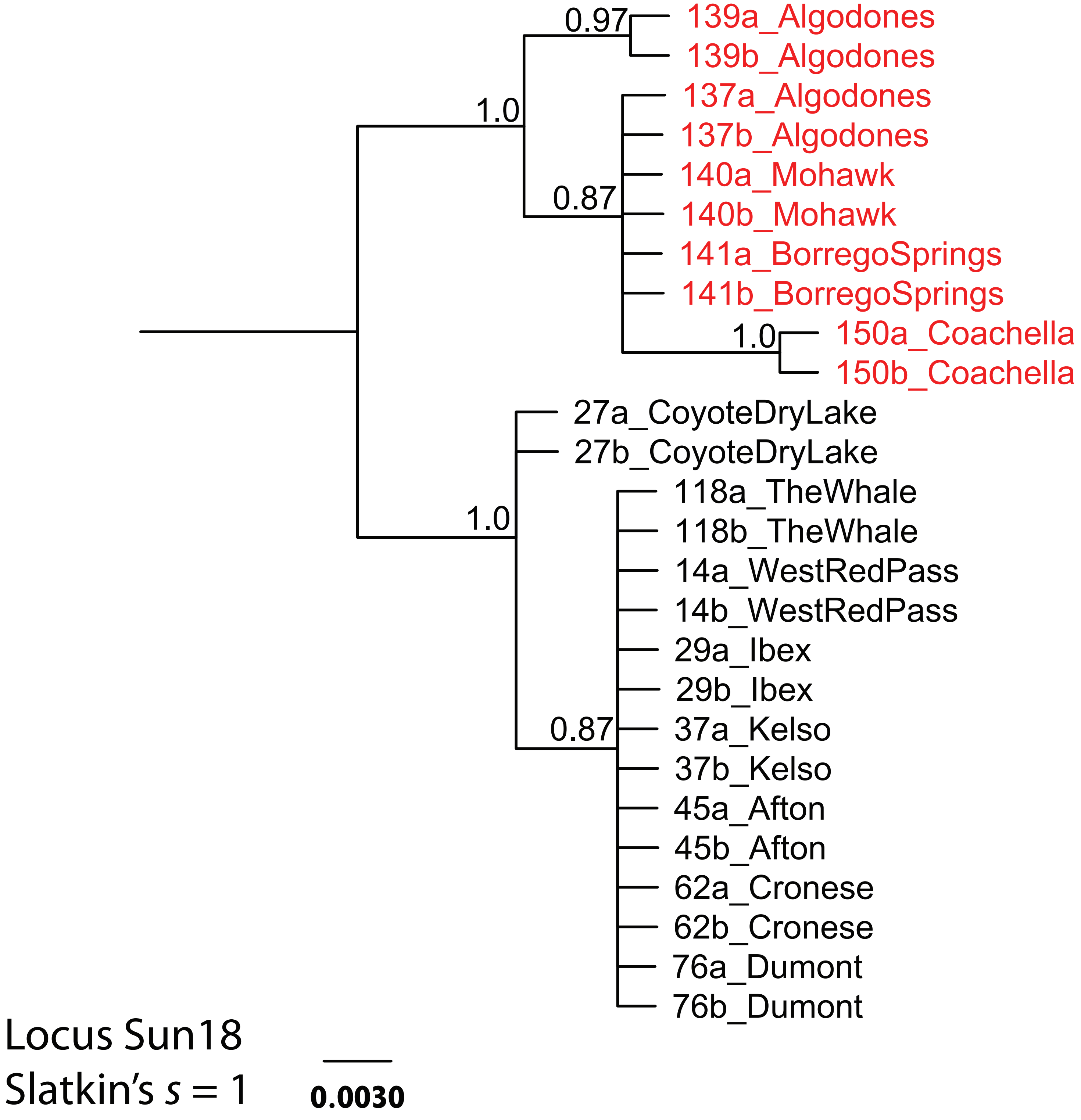

0.97
139a_Algodones
139b_Algodones
1.0
137a_Algodones
137b_Algodones
140a_Mohawk
0.87
140b_Mohawk
141a_BorregoSprings
141b_BorregoSprings
1.0
150a_Coachella
150b_Coachella
27a_CoyoteDryLake
27b_CoyoteDryLake
118a_TheWhale
118b_TheWhale
1.0
14a_WestRedPass
14b_WestRedPass
29a_Ibex
29b_Ibex
0.87
37a_Kelso
37b_Kelso
45a_Afton
45b_Afton
62a_Cronese
62b_Cronese
76a_Dumont
76b_Dumont
Locus Sun18
Slatkin's s = 1
0.0030

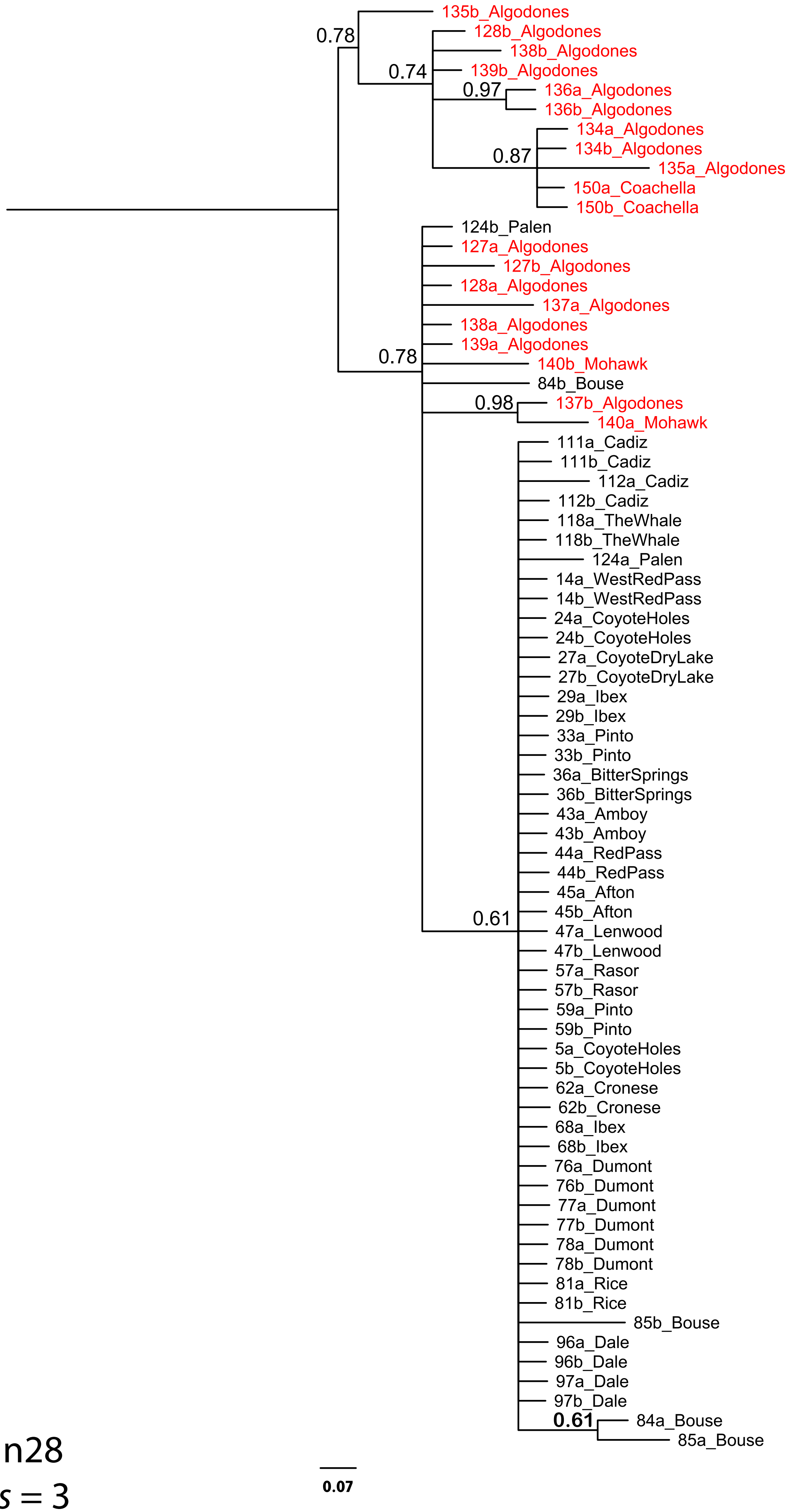

Locus Sun28

Slatkin's $s = 3$

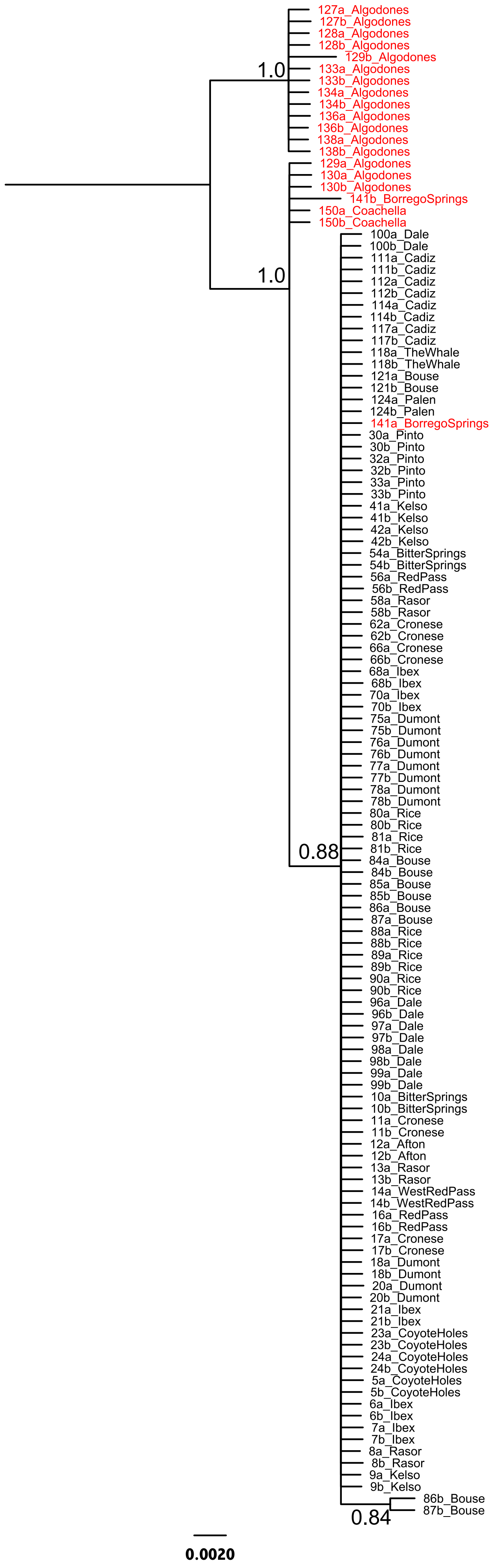

Locus Uma03

Slatkin's *s* = 2

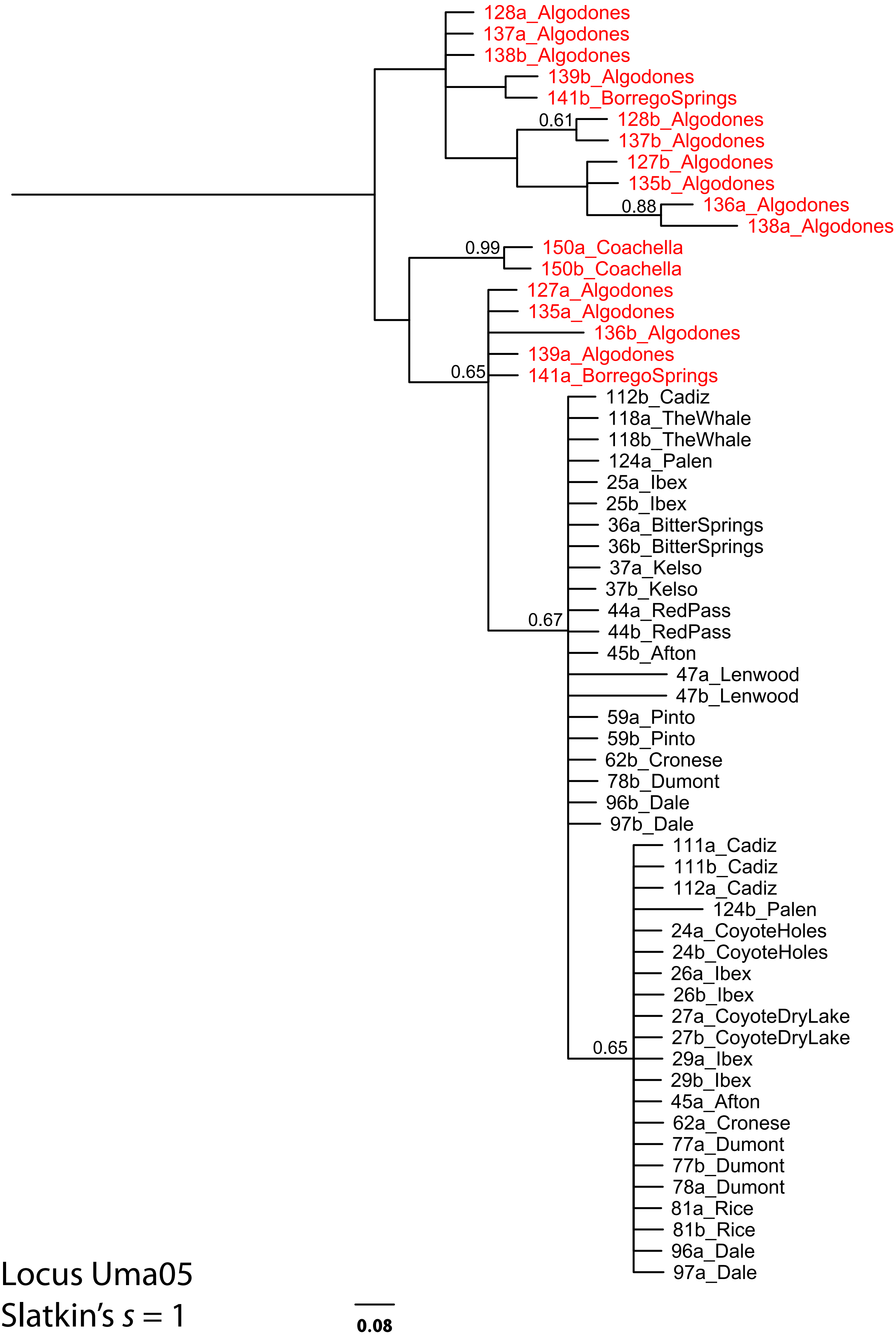

Locus Uma05

Slatkin's *s* = 1

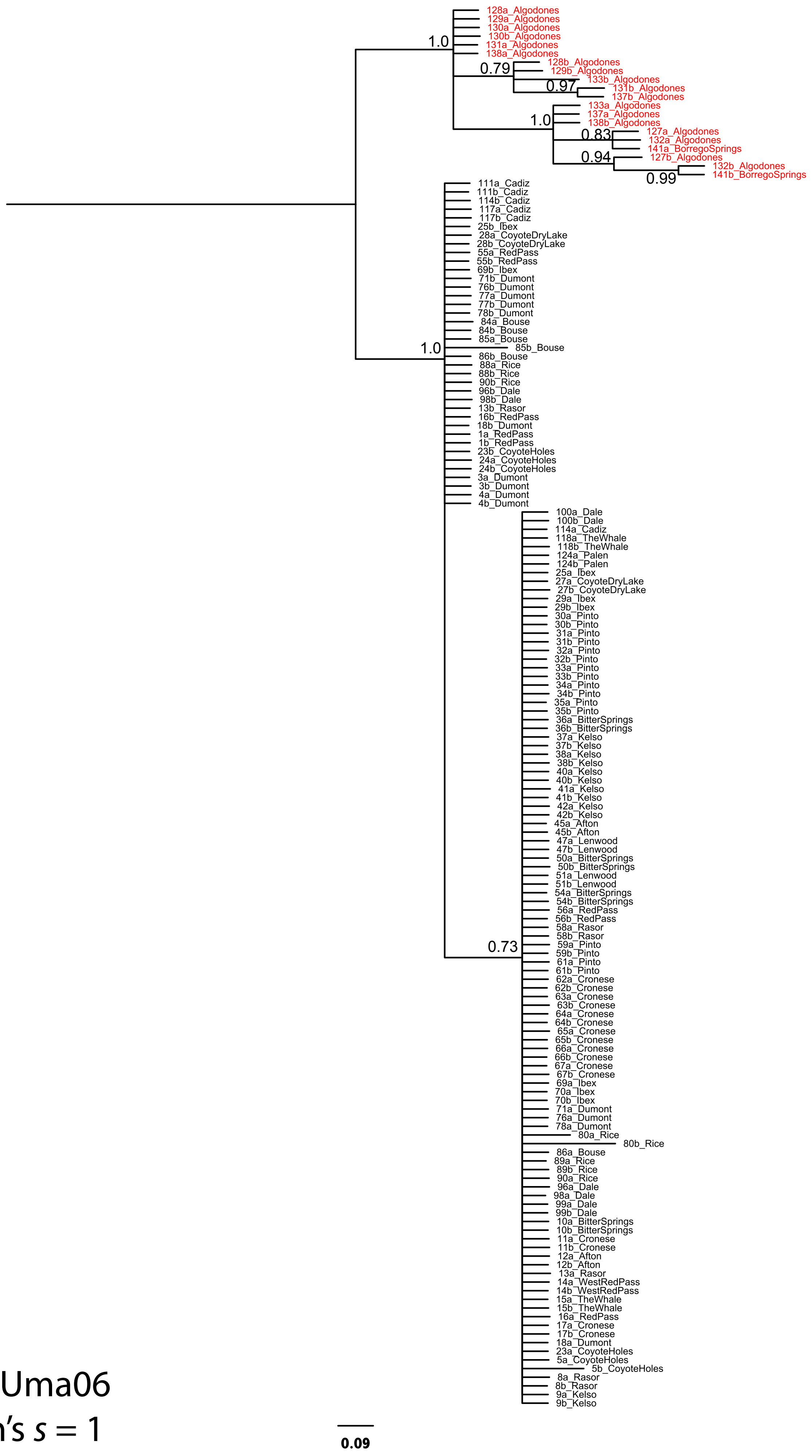

Locus Uma06

Slatkin's *s* = 1

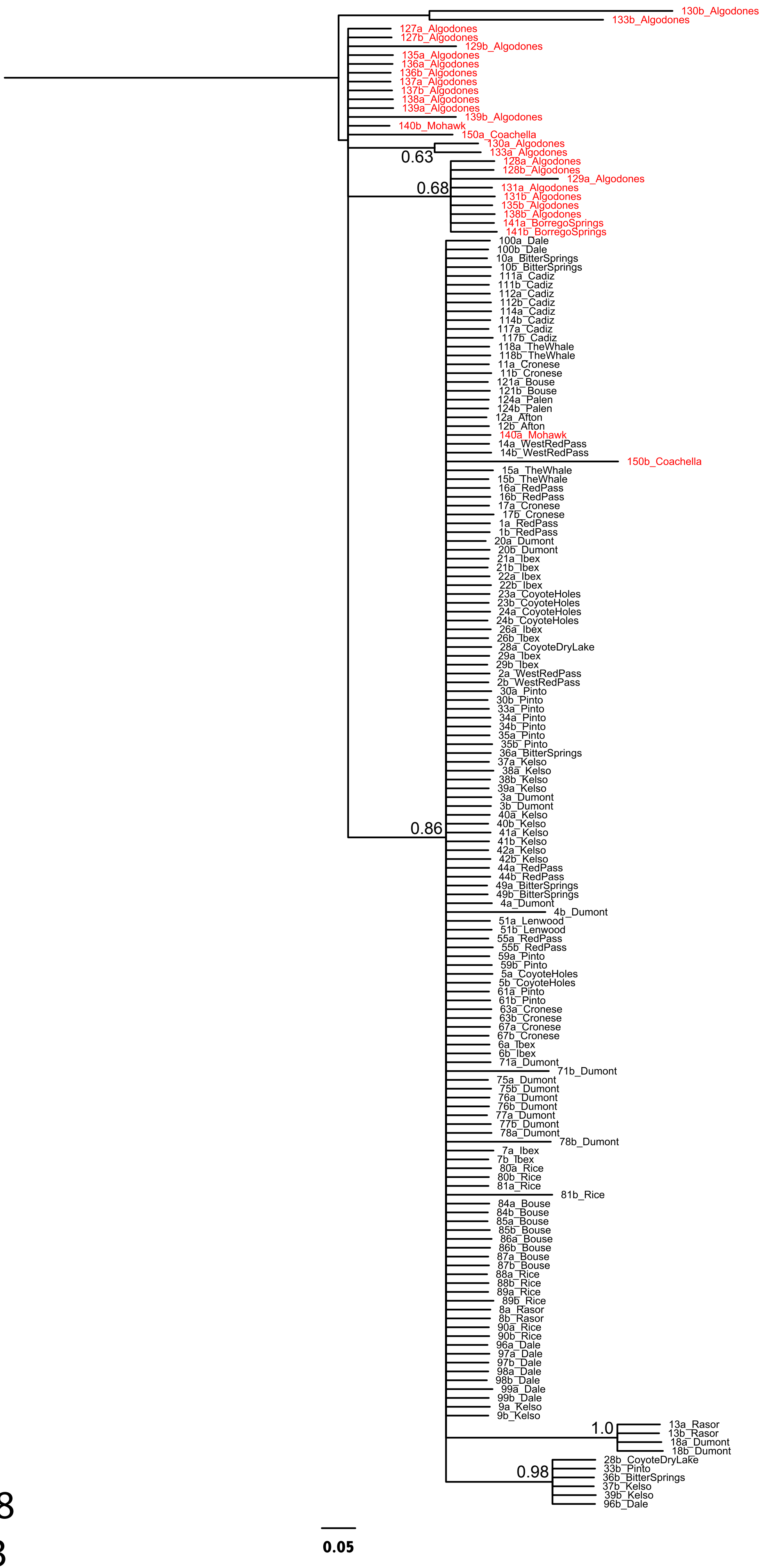

Locus Uma08

Slatkin's $s = 3$
