# Supplementary material for: Speciation, population structure, and demographic history of the Mojave Fringe-toed Lizard (Uma scoparia), a species of conservation concern
Source: Ecol Evol. 2014 May 24;4(12):2546–62. doi: 10.1002/ece3.1111 (PMC4203297; doi:10.1002/ece3.1111)
Supplement: Supplementary file 9 — Table S1. Sampling matrix with individual lizard information and GPS coordinates for all sequences used in this study. The numeral 1 denotes the presence of sequence data for that individual at that locus. [file ece30004-2546-sd9.pdf]

Table S1.

Specimen #	Species	Locality	Latitude	Longitude	Uma03	Uma06	Uma07	Uma08	Uma05	Sun07	Sun08	Sun10	Sun12	Sun18	Sun28	BDNF	RAG-1	PNN	R35
1	*U. scoparia*	Red Pass Dune	35.2483	-116.3629		1		1											
2	*U. scoparia*	West Red Pass	35.2577	-116.3766				1											
3	*U. scoparia*	Dumont Dunes	35.6697	-116.2387		1		1											
4	*U. scoparia*	Dumont Dunes	35.6962	-116.1878		1	1	1											
5	*U. scoparia*	Coyote Holes	35.6442	-115.9544	1	1	1	1		1		1	1		1	1	1	1	1
6	*U. scoparia*	Ibex Dunes	35.6841	-116.3681	1		1	1											
7	*U. scoparia*	Ibex Dunes	35.6877	-116.3704	1		1	1											
8	*U. scoparia*	Rasor Road	35.0828	-116.1584	1	1	1	1											
9	*U. scoparia*	Kelso Dunes	34.8912	-115.7203	1	1	1	1											
10	*U. scoparia*	Bitter Springs	35.2321	-116.4377	1	1		1											
11	*U. scoparia*	Cronese Lakes	35.1438	-116.2927	1	1	1	1											
12	*U. scoparia*	Afton Canyon	35.0433	-116.4159	1	1	1	1											
13	*U. scoparia*	Rasor Road	35.0829	-116.1589	1	1	1	1											
14	*U. scoparia*	West Red Pass	35.2547	-116.3778	1	1		1		1		1	1	1	1	1	1	1	1
15	*U. scoparia*	The Whale	35.2200	-116.4693		1	1	1											
16	*U. scoparia*	Red Pass Dune	35.2479	-116.3636	1	1		1											
17	*U. scoparia*	Cronese Lakes	35.1419	-116.2970	1	1	1	1											
18	*U. scoparia*	Dumont Dunes	35.6931	-116.1841	1	1		1											
20	*U. scoparia*	Dumont Dunes	35.6694	-116.2392	1			1											
21	*U. scoparia*	Ibex Dunes	35.6878	-116.3707	1		1	1											
22	*U. scoparia*	Ibex Dunes	35.6836	-116.3630			1	1											
23	*U. scoparia*	Coyote Holes	35.6442	-115.9544	1	1	1	1		1		1	1			1	1	1	1
24	*U. scoparia*	Coyote Holes	35.6425	-115.9545	1	1		1	1	1		1	1		1	1	1	1	1
25	*U. scoparia*	Ibex Dunes	35.6935	-116.3660		1	1		1			1	1			1		1	1
26	*U. scoparia*	Ibex Dunes	35.6787	-116.3666			1	1	1										
27	*U. scoparia*	Coyote Dry Lake	35.1023	-116.7571		1	1		1	1		1	1	1	1	1	1	1	1
28	*U. scoparia*	Coyote Dry Lake	35.1040	-116.7586		1	1	1											
29	*U. scoparia*	Ibex Dunes	35.6718	-116.3696		1	1	1	1	1	1		1	1	1	1	1	1	1
30	*U. scoparia*	Pinto Basin	33.9144	-115.8258	1	1	1	1											
31	*U. scoparia*	Pinto Basin	33.9144	-115.8258		1	1												
32	*U. scoparia*	Pinto Basin	33.9140	-115.8254	1	1	1												
33	*U. scoparia*	Pinto Basin	33.9126	-115.8213	1	1		1							1			1	1
34	*U. scoparia*	Pinto Basin	33.9123	-115.8208		1	1	1											
35	*U. scoparia*	Pinto Basin	33.9145	-115.8261		1	1	1											
36	*U. scoparia*	Bitter Springs	35.2323	-116.4418		1	1	1	1	1		1	1		1	1	1	1	1
37	*U. scoparia*	Kelso Dunes	34.8921	-115.7142		1		1	1	1		1	1	1		1	1	1	1
38	*U. scoparia*	Kelso Dunes	34.8914	-115.7147		1		1											
39	*U. scoparia*	Kelso Dunes	34.8915	-115.7156			1	1											
40	*U. scoparia*	Kelso Dunes	34.8919	-115.7143		1		1											
41	*U. scoparia*	Kelso Dunes	34.8908	-115.7237	1	1	1	1											
42	*U. scoparia*	Kelso Dunes	34.8858	-115.7236	1	1		1											
43	*U. scoparia*	Amboy Crater	34.5643	-115.8003			1			1		1	1		1	1	1	1	1
44	*U. scoparia*	Red Pass Dune	35.2483	-116.3643			1	1	1	1		1	1		1	1	1	1	1
45	*U. scoparia*	Afton Canyon	35.0435	-116.4181		1			1	1		1	1	1	1	1	1		1
47	*U. scoparia*	Lenwood Rd	34.8894	-117.1299		1			1	1		1			1		1	1	1
48	*U. scoparia*	Bitter Springs	35.2317	-116.4353			1												
49	*U. scoparia*	Bitter Springs	35.2299	-116.4326			1	1											
50	*U. scoparia*	Bitter Springs	35.2302	-116.4517		1	1												
51	*U. scoparia*	Lenwood Rd	34.8906	-117.1240		1	1	1											
52	*U. scoparia*	Afton Canyon	35.0434	-116.4185			1												
54	*U. scoparia*	Bitter Springs	35.2321	-116.4383	1	1	1												
55	*U. scoparia*	Red Pass Dune	35.2492	-116.3635		1	1	1											
56	*U. scoparia*	Red Pass Dune	35.2488	-116.3639	1	1	1												
57	*U. scoparia*	Rasor Road	35.0783	-116.1586								1	1		1	1	1		1
58	*U. scoparia*	Rasor Road	35.0786	-116.1604	1	1	1												
59	*U. scoparia*	Pinto Basin	33.9137	-115.8232		1	1	1	1	1	1		1		1	1	1	1	1
60	*U. scoparia*	Pinto Basin	33.9125	-115.8208													1		
61	*U. scoparia*	Pinto Basin	33.9138	-115.8242		1		1											
62	*U. scoparia*	Cronese Lakes	35.1441	-116.2946	1	1			1	1		1	1	1	1	1	1	1	1
63	*U. scoparia*	Cronese Lakes	35.1431	-116.2963		1	1	1											
64	*U. scoparia*	Cronese Lakes	35.1431	-116.2920		1													
65	*U. scoparia*	Cronese Lakes	35.1422	-116.2912		1	1												
66	*U. scoparia*	Cronese Lakes	35.1422	-116.2911	1	1	1												
67	*U. scoparia*	Cronese Lakes	35.1416	-116.2904		1	1	1											
68	*U. scoparia*	Ibex Dunes	35.7097	-116.3732	1		1								1			1	1
69	*U. scoparia*	Ibex Dunes	35.7032	-116.3657		1													
70	*U. scoparia*	Ibex Dunes	35.7029	-116.3655	1	1													
71	*U. scoparia*	Dumont Dunes	35.6685	-116.2418		1	1	1											
75	*U. scoparia*	Dumont Dunes	35.6678	-116.2382	1		1	1		1		1	1			1		1	1
76	*U. scoparia*	Dumont Dunes	35.6663	-116.2382	1	1	1	1		1		1	1	1	1	1	1	1	1
77	*U. scoparia*	Dumont Dunes	35.6687	-116.2364	1	1	1	1	1	1	1	1	1		1	1	1		1
78	*U. scoparia*	Dumont Dunes	35.6616	-116.2321	1	1	1	1	1	1	1	1	1		1	1		1	
80	*U. scoparia*	Rice Valley	34.0512	-114.8666	1	1	1	1		1	1	1	1			1	1	1	1
81	*U. scoparia*	Rice Valley	34.0544	-114.8652	1		1	1	1	1		1	1		1	1	1	1	1
84	*U. scoparia*	Bouse Wash	34.0326	-114.2516	1	1	1	1		1		1	1		1	1	1	1	1
85	*U. scoparia*	Bouse Wash	34.0742	-114.2673	1	1	1	1		1	1	1	1		1	1	1		1
86	*U. scoparia*	Bouse Wash	34.0743	-114.2677	1	1	1	1											
87	*U. scoparia*	Bouse Wash	34.0747	-114.2707	1		1	1											
88	*U. scoparia*	Rice Valley	34.0503	-114.8636	1	1	1	1											
89	*U. scoparia*	Rice Valley	34.0503	-114.8636	1	1	1	1											
90	*U. scoparia*	Rice Valley	34.0435	-114.8624	1	1	1	1											
96	*U. scoparia*	Dale Lake	34.1002	-115.6267	1	1	1	1	1	1		1	1		1		1	1	1
97	*U. scoparia*	Dale Lake	34.1012	-115.6253	1		1	1	1	1	1	1	1		1	1	1		1
98	*U. scoparia*	Dale Lake	34.1030	-115.6243	1	1	1	1											
99	*U. scoparia*	Dale Lake	34.1042	-115.6240	1	1	1	1											
100	*U. scoparia*	Dale Lake	34.1065	-115.6239	1	1	1	1											
111	*U. scoparia*	Cadiz Dunes	34.3932	-115.4260	1	1	1	1	1	1	1	1	1		1	1		1	1
112	*U. scoparia*	Cadiz Dunes	34.3935	-115.4281	1		1	1	1	1	1	1	1		1	1	1	1	1
114	*U. scoparia*	Cadiz Dunes	34.3953	-115.4297	1	1	1	1											
117	*U. scoparia*	Cadiz Dunes	34.3948	-115.4257	1	1		1											
118	*U. scoparia*	The Whale	35.2187	-116.4692	1	1	1	1	1	1	1			1	1	1	1	1	1
121	*U. scoparia*	Bouse Wash	34.0742	-114.2668	1		1	1											
124	*U. scoparia*	Palen Dunes	33.8023	-115.1849	1	1	1	1	1	1	1	1	1		1	1	1	1	
127	*U. notata*	Algodones	32.9859	-115.1325	1	1		1	1	1			1		1		1		
128	*U. notata*	Algodones	32.9876	-115.1314	1	1	1	1	1	1		1	1		1	1			
129	*U. notata*	Algodones	32.9879	-115.1312	1	1	1	1											
130	*U. notata*	Algodones	32.9885	-115.1311	1	1		1											
131	*U. notata*	Algodones	32.9885	-115.1311		1	1	1											
132	*U. notata*	Algodones	32.9904	-115.1337		1	1												
133	*U. notata*	Algodones	32.9906	-115.1336	1	1		1											
134	*U. notata*	Algodones	32.9923	-115.1329	1					1		1	1		1	1	1	1	1
135	*U. notata*	Algodones	32.9921	-115.1322			1	1	1	1	1	1	1		1	1	1		1
136	*U. notata*	Algodones	32.9918	-115.1318	1		1	1	1	1		1	1		1	1	1	1	1
137	*U. notata*	Algodones	32.9895	-115.1333		1	1	1	1	1		1	1	1	1	1	1	1	1
138	*U. notata*	Algodones	32.9900	-115.1307	1	1		1	1	1		1	1		1	1	1		1
139	*U. notata*	Algodones	32.9885	-115.1297				1	1	1		1	1	1	1	1	1	1	1
140	*U. rufopunctata*	Mohawk Dunes	32.6974	-113.8095				1		1		1		1	1	1	1		1
141	*U. notata*	Borrego Springs	33.2474	-116.2957	1	1	1	1	1	1		1		1		1	1	1	1
150	*U. inornata*	Coachella Valley	33.8470	-116.3919	1		1	1	1			1	1	1	1	1	1	1	1
