# Supplementary material for: Speciation, population structure, and demographic history of the Mojave Fringe-toed Lizard (Uma scoparia), a species of conservation concern
Source: Ecol Evol. 2014 May 24;4(12):2546–62. doi: 10.1002/ece3.1111 (PMC4203297; doi:10.1002/ece3.1111)
Supplement: Supplementary file 10 — Table S2. PCR primers, annealing temperatures, and literature sources used in this study. PCR reactions consisted of a denaturation step (94° C for 1 min), an annealing step (1 min), and an extension step (72° C for 1 min), repeated 35 times. [file ece30004-2546-sd10.pdf]

Table S2.

Locus	Forward Primer	Reverse Primer	Annealing Temp. (° C)	Source
BDNF	GAC CAT CCT TTT CCT KAC TAT GGT TAT TTC ATA CTT	CTA TCT TCC CCT TTT AAT GGT CAG TGT ACA AAC	61	Leaché 2009
RAG-1	CAA AGT RAG ATC ACT TGA GAA GC	ACT TGY AGC TTG AGT TCT CTT AG RCG	55	Leaché 2009
PNN	ACA GGT AAT CAG CAC AAT GAY GTA GA	TCT YYT GCC TGA YCG ACT ACT YTC TGA	57	Leaché 2009
R35	GAC TGT GGA YGA YCT GAT CAG TGT GGT GCC	GCC AAA ATG AGS GAG AAR CGC TTC TGA GC	66.5	Leaché 2009
Sun07	TTT CTG TCA CGA TGA AAA TTG TAA ACT A	TAA ACA CAA TGC TCA CAT TAG GAA AAA T	61	Rosenblum et al. 2007
Sun08	CTC TTG AAG TTC ACA GGG TTT TCT TAG	TAG CCT AGC TTC CTT ACA GTT TGA TAC	63	Rosenblum et al. 2007
Sun10	CAG AAA GTA AAT CCA CTG TAG CTA GGA	CTA ATA ATG GCA TAG CAA GGA GTG TAG	61	Rosenblum et al. 2007
Sun12	TAC AGA GTC TCC TCT TGA CTG GAT ATT	TTG GTA CAC TAA CTC AAG CAA ACC T	54	Rosenblum et al. 2007
Sun18	ATG ACA GAA GTT GTG GTT CAA CAG TAT	AGT GAG ATA GAA GTG GCT TTC TGA TTA C	61	Rosenblum et al. 2007
Sun28	AAT CTT ATT TCT GCA GTT GAT GTA CTT T	ATA AAT GCA ATG CCA CAA ATA TAA TAA G	57.5	Rosenblum et al. 2007
Uma03	CGC ATG AGA ATT CTG TGT TA	TGC TAA TGC TGA TGA AAA TG	59	This study
Uma05	TTG TTC CCA TAG CTG AAA CT	TTT GGT AAT GAA TCC CAC TC	50	This study
Uma06	GCT CTT ACC CTC TGT TTG AA	AGG TGA CAG ATG GAG CTA AA	50	This study
Uma08	AGG TGT TTT GAA CTG CAA CT	ACT TTC CCA GCA CAT AAA AA	59	This study
