# Supplementary material for: Speciation, population structure, and demographic history of the Mojave Fringe-toed Lizard (Uma scoparia), a species of conservation concern
Source: Ecol Evol. 2014 May 24;4(12):2546–62. doi: 10.1002/ece3.1111 (PMC4203297; doi:10.1002/ece3.1111)
Supplement: Supplementary file 11 — Table S3. Results of power analyses for each of the six a priori speciation models. The program Mesquite was used to generate 100 simulated 14-locus datasets with characteristics similar to the observed data including an U. scoparia/U. notata divergence time of 0.5 Ma. These “pseudo-observed” datasets were then individually compared to each null distribution via PST tests (see File S1, S2 for details). The percentage of tests yielding false negative results (Type II error) is shown in the first column, whereas the percentage of tests in which the null hypothesis was correctly rejected (statistical power) is shown in the second column. [file ece30004-2546-sd11.pdf]

Table S3.

| Speciation Model | % False Negative Tests | Power of Test |
|---|---|---|
| 1a | 0 | 100 |
| 1b | 36 | 64 |
| 3a | 53 | 47 |
| 3b | 53 | 47 |
| 4a | 53 | 47 |
| 4b | 53 | 47 |
